# Engineering the substrate scope of the thermostable phenolic acid decarboxylase N31 towards sterically hindered phenolic acids

**DOI:** 10.1002/pro.70692

**Published:** 2026-07-12

**Authors:** Kristin K. F. Bauer, Sonja Vaupel, Jacques Gay, Eva Vos, Aron Wanz, Daniel Kracher, Tobias Schöngassner, Lars‐Erik Meyer, Shina C. L. Kamerlin, Selin Kara, Robert Kourist

**Affiliations:** ^1^ Institute of Molecular Biotechnology, Graz University of Technology Graz Austria; ^2^ Institute of Technical Chemistry, Leibniz University Hannover Hannover Germany; ^3^ School of Chemistry and Biochemistry, Georgia Institute of Technology Atlanta Georgia USA; ^4^ BioTechMed‐Graz Graz Austria; ^5^ School of Chemical and Biomolecular Engineering, Georgia Institute of Technology Atlanta Georgia USA; ^6^ Department of Chemistry Lund University Lund Sweden; ^7^ Biocatalysis and Bioprocessing Group, Department of Biological and Chemical Engineering Aarhus University Aarhus Denmark

**Keywords:** ancestral sequence reconstruction, biobased polymers, biocatalysis, canolol, combinatorial library, directed enzyme evolution, enzymatic decarboxylation, enzyme engineering, molecular dynamics simulations, phenolic acid decarboxylase, renewable chemicals

## Abstract

Phenolic acid decarboxylases (PADs) convert bio‐based hydroxycinnamic acids into valuable hydroxystyrene monomers under mild reaction conditions. These compounds are in high demand in polymer production, cosmetics, and flavoring. Especially 4‐vinyl syringol, the decarboxylation product from sinapic acid, generates polymers with similar thermal stability and higher glass transition temperatures than vinyl guaiacol, the decarboxylation product from ferulic acid. However, natural PAD enzymes typically show slow turnover with sinapic acid. In addition, establishing a viable industrial process requires enzymes operating under elevated temperatures. To tackle these issues, we assessed five thermostable ancestral PADs towards their activity and stability for the conversion of ferulic acid and sinapic acid at different temperatures. A combinatorial active site library was prepared for the most thermostable ancestor. We expanded the substrate scope of a selected PAD ancestor to include sinapic acid through directed mutagenesis. A trade‐off between ferulic−/caffeic acid and sinapic acid was observed and investigated via molecular dynamics simulations. The most stable ancestor was identified with a half‐life of 3.65 days, analyzed at 50°C. We found the Ile29Ser‐Leu80Ser‐Ile93Ala triple mutation (SSA) to effectively expand the substrate scope with an 11‐fold increase in catalytic efficiency for sinapic acid, and a half‐life of 1.12 days at 50°C, being approximately 1.6‐fold higher than the frequently used PAD from *Bacillus subtilis*.

## 1 INTRODUCTION

**SCHEME 1:**
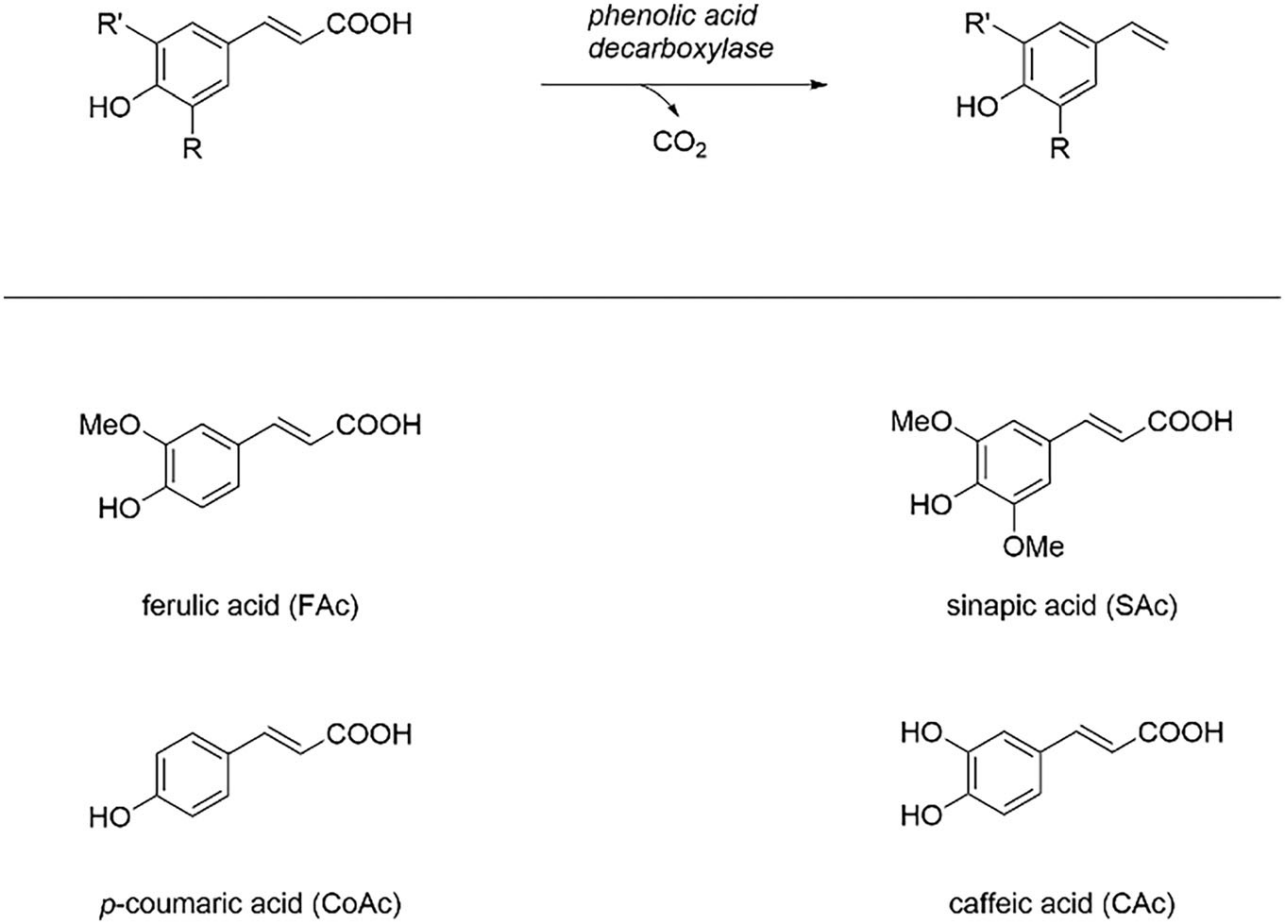
Phenolic acid decarboxylase‐catalyzed decarboxylation of different substrates.

The demand for polymers has constantly risen over the last decades. The plastics industry had an annual output of approximately 400 million tons in 2022, with over 90% being fossil‐based (Plastics Europe, 2023). New synthetic routes must be established to meet these current and future challenges. One strategy is to use bio‐based polymer additives built from hydroxystyrene monomers. These may be synthesized from the decarboxylated form of lignin‐based phenolic acids such as ferulic acid (FAc), sinapic acid (SAc), *p*‐coumaric acid (CoAc), and caffeic acid (CAc) (Petermeier et al., 2022). These can be found in many plants and can be obtained as agricultural residues or during pulp and paper production (Haile et al., 2021; Raj et al., 2022). The biotransformation approach is an excellent tool for producing bulk chemicals with high throughput in a sustainable manner under mild reaction conditions (Domínguez de María, 2021). Syringol‐based polymers produced through reversible addition‐fragmentation transfer (RAFT) exhibit comparable thermal stability and higher glass transition temperatures than their guaiacol‐based counterparts, highlighting the interest in using SAc as a substrate (Holmberg et al., 2019). The decarboxylation of hydroxycinnamic acids can be achieved by phenolic acid decarboxylases (PADs), primarily found in bacteria and fungi, and belonging to the class of cofactor‐free decarboxylases (Scheme 1) (Frank et al., 2012; Maeda et al., 2018).

Phenolic acid decarboxylase from *Bacillus subtilis* (*Bs*PAD) was first crystallized as a catalytically inactive T19A variant in conjugation with CoAc by Frank et al. (Frank et al., 2012). The decarboxylation mechanism is initiated by deprotonating the phenolic hydroxy, yielding a quinone methide intermediate that subsequently undergoes decarboxylation (Sheng et al., 2015). PADs can also be applied for reverse *β*‐carboxylation or hydration of vinyl derivatives (Sheng et al., 2015; Wuensch et al., 2015). While *Bs*PAD shows good activity towards FAc (80 ± 13 U/mg), CoAc (40 ± 4 U/mg) or CAc (18 ± 1 U/mg), it is not able to convert SAc efficiently (Baraibar et al., 2016; Petermeier et al., 2022; Schweiger et al., 2019). This limitation applies to most known PADs thus far, such as PAD from *B. licheniformis* CGMCC7172 with a specific activity of 0.3 U/mg for SAc. A PAD from the brown‐rot fungus *N. lepideus* (*Nl*PAD) was reported with a *V*_max_ for SAc of 10 U/mg, while possessing a half‐life time of 12.4 h at 55°C (Lomascolo et al., 2023; Odinot et al., 2024).

Several protein engineering campaigns have aimed to increase the SAc conversion of bacterial PADs (Li et al., 2021; Morley et al., 2013; Schweiger et al., 2019). For example, in 2013, Morley et al. engineered a wild‐type *Bacillus pumilus* PAD by site‐directed mutagenesis, with the best‐performing single mutant Ile85Ala showing a specific activity of 41.3 U/mg for SAc. However, the catalytic activity towards FAc decreased seven‐fold as a tradeoff (Morley et al., 2013). Following up, Schweiger et al. performed site‐directed mutagenesis at the equivalent position Ile85 to Ala, Val, and Leu on *Bs*PAD, yielding a SAc decarboxylation activity of 10 ± 2 U/mg, 3.0 ± 0.5 U/mg and 0 U/mg, respectively. Therefore, the Ile85Ala mutation was transferrable to *Bs*PAD, resulting in higher activities towards SAc (Schweiger et al., 2019).

A thermally stable enzyme is of great interest for process intensification, as (i) the conversion rate can be faster at higher temperatures and (ii) non‐aqueous conditions can be used, allowing better substrate solubility. In previous study, we generated a thermally stable ancestral PAD with an unfolding temperature of 78°C (24°C higher than *Bs*PAD) and a half‐life time of 45 h (2,700‐fold higher than *Bs*PAD) at 60°C via ancestral sequence reconstruction (ASR) (Myrtollari et al., 2024). In this study, we compared the kinetics and thermal stabilities of five previously identified PAD ancestors. Subsequently, we extended the substrate scope of the most thermally stable variant, PAD N31, through an engineering approach to include SAc and characterized the resulting mutant. We suggest that this thermally stable, SAc‐accepting mutant opens new opportunities for efficient substrate conversion under mild reaction conditions.

## 2 RESULTS AND DISCUSSION

### 2.1 Characterization of ancestral PAD

**FIGURE 1:**
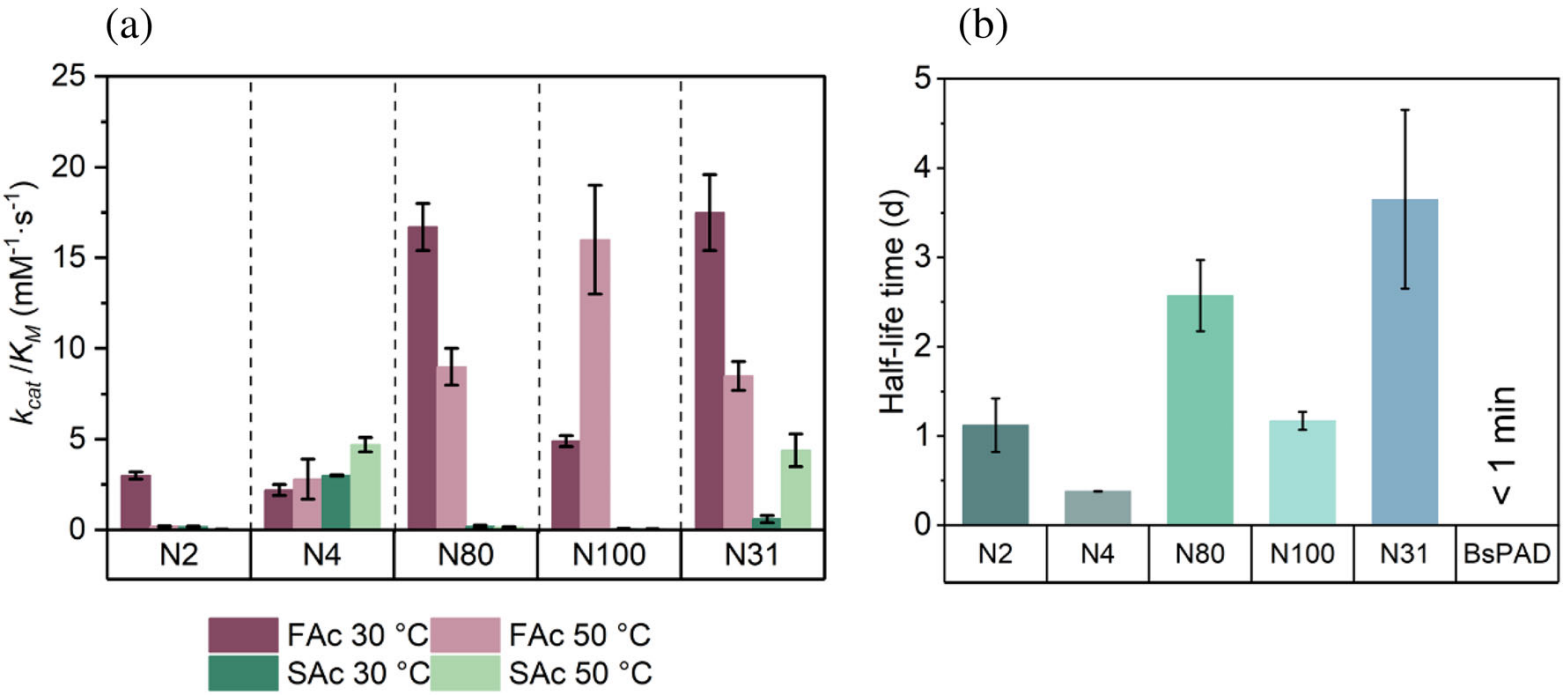
(a) Schematic representation of five PAD ancestors' catalytic efficiency (*k*_cat_/*K*_M_) values. (b) Half‐life of the five ancestors in buffer (50 mM KPi, pH 6) at 50°C. The error bars are calculated from duplicate experiments.

To identify potential ancestors for the synthesis of 4‐vinyl syringol (4‐VS) at elevated reaction temperatures, we screened a panel of previously identified PAD ancestors with melting temperatures higher than *Bs*PAD and good expression levels for their substrate scope (Myrtollari et al., 2024). For all kinetic measurements, a photometric assay that measures substrate depletion was used (Terholsen et al., 2023), avoiding any impact from the instability of the styrene products. This included (i) determining Michaelis–Menten steady‐state kinetics, (ii) analyzing product inhibition, and (iii) determining half‐life times. All catalytic efficiency (*k*_cat_/*K*_M_) values are summarized in Figure 1a, which shows that all investigated ancestors favored FAc as a substrate. The highest catalytic efficiency was observed for N31 with a *k*_cat_/*K*_M_ of 17.5 ± 2.1 s^−1^ mM^−1^. However, it should be noted that zero‐order kinetics were not achieved for N2 and N100 using FAc as a substrate at 30°C (Figure S2a,d and Table 1) due to the limited aqueous solubility of the substrate. Thus, we assumed a linear dependency of substrate conversion in the operational window with a *K*_*M*,FAc_ >>[FAc]. As a consequence, *V*_max_ */K*_*M*_ can be calculated from the linear slope. This technique was additionally applied to N2 with FAc as substrate at 50°C (Figure S3a) and N2 with SAc as substrate at 50°C (Figure S5a). The results show that the *k*_cat_/*K*_*M*_ values remained largely unchanged despite an increase in the reaction temperature. The catalytic efficiency dropped with rising temperature in the case of the ancestors N2 and for FAc for N31 (Figure S3 and Table 1). This non‐Arrhenius behavior might be explained by the deactivation of the enzyme by conformational changes occurring at elevated temperature. Higher temperatures may introduce too much flexibility and reduce substrate binding if the active site geometry in the artificial ancestral enzymes is suboptimal. Only N31 showed increased specific activity at higher temperatures (7.3 ± 2.0 U/mg, Table 1). When using SAc as a substrate, all ancestors showed very poor catalytic activities, with N31 having the highest specific activity of 2.0 **±** 0.1 U/mg. In this case, the *k*_cat_/*K*_*M*_ value is higher for 30°C (Figures S4 and S5 and Table 1).

**TABLE 1:** Summary of kinetic data for PAD ancestors for FAc and SAc at 30°C and 50°C.

Substrate		30°C				50°C			
PAD ancestor		*K*_*M*_ (mM)	*V*_max_ (U mg^−1^)	*V*_max_/*K*_*M*_ (U × mg^−1^× mM^−1^)^a^	*k*_cat_/*K*_*M*_ (s^−1^ × mM^−1^)	*K*_*M*_ (mM)	*V*_max_ (U mg^−1^)	*V*_max_/*K*_*M*_ (U × mg^−1^× mM^−1^)^a^	*k*_cat_/*K*_*M*_ (s^−1^ × mM^−1^)
N2	FAc	–	–	8.7 ± 0.6	3.0 ± 0.2	–	–	0.58 ± 0.04	0.2 ± 0.02
	SAc	0.4 ± 0.08	0.2 ± 0.01	–	0.18 ± 0.02	–	–	0.11 ± 0.004	0.04 ± 0.002
N4	FAc	1.9 ± 0.8	11 ± 2	–	2 ± 0.9	0.3 ± 0.3	2.4 ± 0.5	–	2.8 ± 1.1
	SAc	1.4 ± 0.3	0.1 ± 0.01	–	3 ± 0.04	2.7 ± 0.7	0.4 ± 0.05	–	4.7 ± 0.4
N80	FAc	4 ± 1	105 ± 18	–	9.2 ± 0.6	1.3 ± 0.3	33 ± 3	–	9 ± 1.0
	SAc	0.15 ± 0.12	0.08 ± 0.01	–	0.19 ± 0.07	4 ± 1	0.48 ± 0.07	–	0.15 ± 0.03
N100	FAc	–	–	13.9 ± 0.8	4.9 ± 0.3	3 ± 1	47 ± 13	–	5.5 ± 0.2
	SAc	1.8 ± 0.5	0.3 ± 0.04	–	0.058 ± 0.007	3.3 ± 0.8	0.5 ± 0.1	–	0.05 ± 0.01
N31	FAc	1 ± 0.4	6 ± 1	–	2.1 ± 0.9	1.5 ± 0.9	7 ± 2	–	1.6 ± 0.3
	SAc	1.6 ± 0.7	2 ± 0.4	–	0.6 ± 0.2	0.16 ± 0.05	2 ± 0.1	–	4.4 ± 0.9

*Note*: Fitting was performed using Origin. GRG Nonlinear Solving Method for nonlinear optimization was used.

^a^ The linear slope (= *V*_max_/K_M_) within the concentration range.

Additionally, the stability of these enzymes was evaluated regarding their half‐lives (*t*_1/2_) at 50°C (Figure 1b). All measured *t*_1/2_ values in the buffer were significantly higher than those of *Bs*PAD (around 1 min). The ancestors N2 and N100 performed similarly with a *t*_1/2_ value around one day. The ancestor N80 achieved a *t*_1/2_ value of around 2.6 days, whereas N4 was inactivated with a *t*_1/2_ of 0.4 days. N31 performed outstandingly with 3.65 days (Figure S6 and Table S5). The data are in excellent agreement with the melting temperatures measured by Myrtollari et al. (2024) using circular dichroism spectroscopy. Ancestors N2 and N4 had an unfolding temperature of approximately 55°C, whereas N80 and N100 denatured at around 65°C, and N31 outperformed the other ancestors at 78°C (Myrtollari et al., 2024).

**FIGURE 2:**
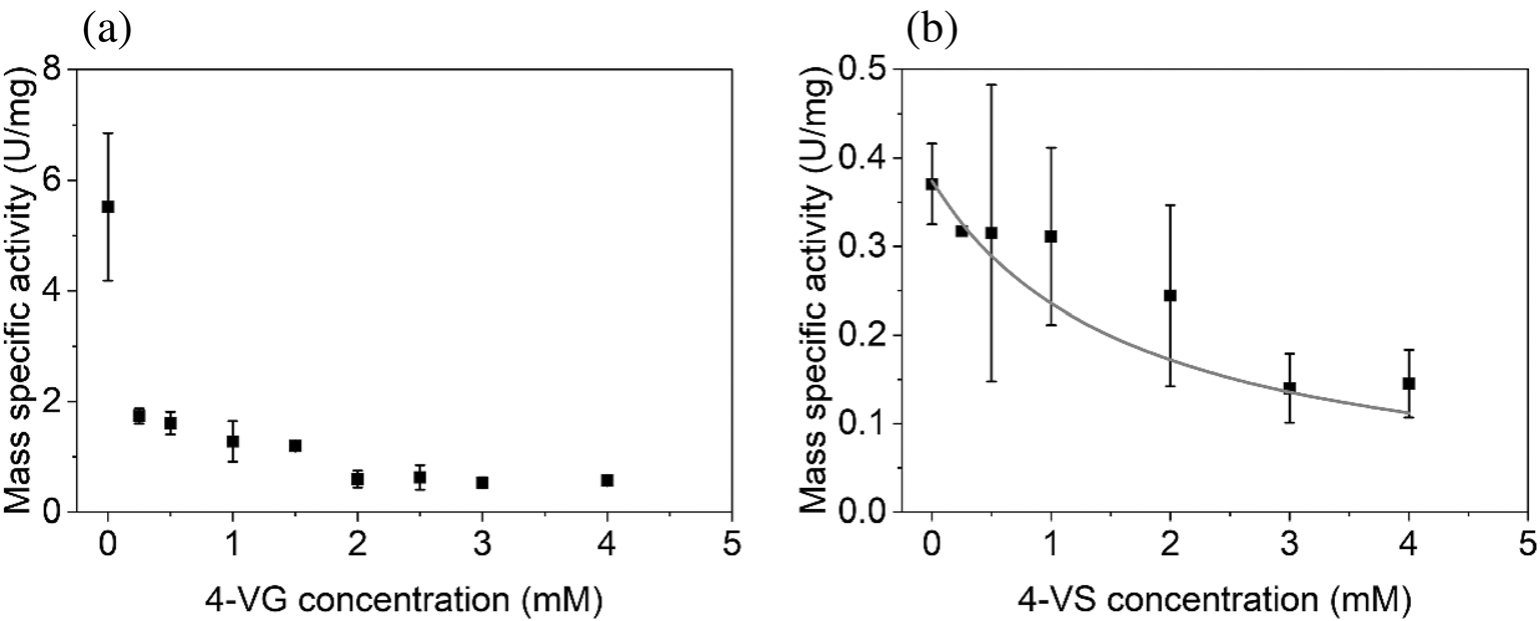
Specific activity for (a) FAc and (b) SAc of N31 in the presence of various concentrations of 4‐VG (product of FAc) and 4‐VS (product of SAc), respectively. (a) Product inhibition of 4‐VG for N31, (b) Product inhibition of 4‐VS for N31. The photometric assay was performed in potassium phosphate buffer (50 mM, pH 6) with 4 mM of FAc and SAc, respectively, with a final volume of 100 μL containing 25 μg enzyme and 0 to 4 mM 4‐VS and 4‐VG, respectively (SAc stock: 200 mM in DMSO). The reaction was conducted at 30°C and observed for 5 min at 344 nm for 4‐VG and 348 nm for 4‐VS. The fitting was performed after Equation 3) (see Section 4).

Investigation of the inhibition by the product 4‐vinyl guaiacol (4‐VG) showed that most ancestors suffer from strong product inhibition (Figure S7 and Table S6). In particular, N2 exhibited significant product inhibition, leading to a drop to 33% of the initial activity upon the addition of 0.25 mM product, corresponding to 5.9% conversion. While variant N4 demonstrated the lowest relative product inhibition, retaining 75% of its activity in the presence of 4 mM 4‐VG, its specific activity remained lower than all other variants under all tested conditions, even when those variants were fully inhibited. The activity of N31 decreased to 32% of its initial activity when adding 0.25 mM 4‐VG, indicating strong product inhibition (Figure 2a). The inhibition is less pronounced for 4‐VS, yielding a *K*_i_ */K*_M_ of 0.3 for N31. Overall, the product inhibition by 4‐VS appeared to be less pronounced, which can be attributed to the generally lower activities. Combining the obtained results from the biocatalytic and thermal stability characterization suggested PAD N31, with its outstanding thermal stability, as the best candidate for an engineering campaign to broaden the substrate scope towards SAc.

### 2.2 Engineering of PAD N31 towards SAc acceptance

To create a stable PAD variant that can convert SAc, we used the most thermally stable ancestor as a template and introduced mutations in the active site. After aligning the crystal structures of N31 (PDB: 8B30) and *B. pumilus* PAD (PDB: 3NAD), we introduced a mutation analogous to that identified by Morley et al. in *B. pumilus* PAD (Ile85Ala) into N31, resulting in the Ile93Ala variant. While this mutation led to a significant 50‐fold enhancement of the specific activity of wt.

*B. pumilus* PAD towards SAc, incorporation of the analog amino acid substitution to N31 rendered the ancestor catalytically inactive. (Morley et al., 2013). We were able to produce N31_Ile93Ala in soluble form in *E. coli* (Figure S1), but no activity towards SAc or FAc could be detected (data not shown). As the direct transfer of the mutation into the thermostable ancestor was not successful, we chose simultaneous saturation mutagenesis as a strategy for expanding the substrate specificity.

**FIGURE 3:**
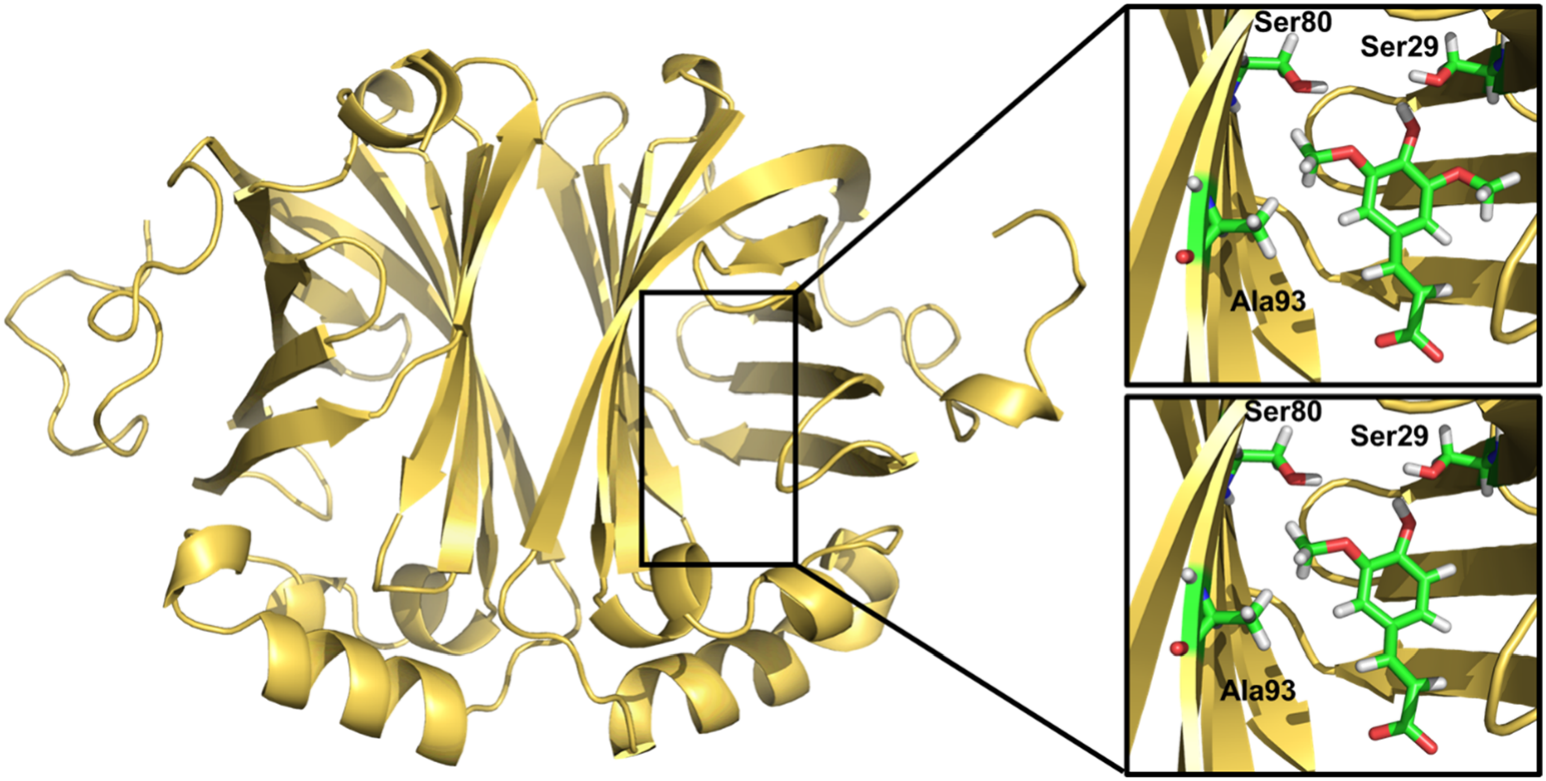
Combinatorial library positions for N31: Positions (29, 80, 93) were chosen for the combinatorial library and replaced by Ser, Ser, and Ala, respectively. Shown are the orientations of SAc and FAc in the mutant. Image created using PyMOL.

Three sites were chosen for the generation of a combinatorial library. The sites and substitutions were selected based on the previous engineering approach performed by Morley et al., as well as a mechanistic study by Sheng et al. (Morley et al., 2013; Sheng et al., 2015). The positions Ile29 and Leu80 were additionally selected due to their proximity towards the O‐methyl substituted ring positions in SAc (Figure 3). While N31 Ile93Ala is inactive, we reasoned that the detrimental effect of the single substitution might be compensated by other active site substitutions by epistatic effects (Alejaldre et al., 2021). Therefore, Ile93 was included in the library. For the site‐specific mutagenesis, primers encoding for Ala, Leu, Ile, Ser, Thr, Val, Gly and the wild type amino acid Ile were used, leading to the creation of single, double and triple mutants.

This strategy resulted in a library size of 7^3^ (= 343) variants for the combinatorial N31 library. The activity of the mutants was assessed with a real‐time spectrophotometric assay, which follows the substrate depletion upon decarboxylation (Terholsen et al., 2023). The screening of the three‐times oversampled combinatorial N31 library (1056 variants) indicated that there is a trade‐off between SAc and CAc/FAc activity (Figure S9). While 167 variants displayed at least a 1.7‐fold activity increase for SAc, approx. 20% (32 variants) of these showed a simultaneous 0.3‐ and 0.4‐fold decrease in activity for CAc and FAc, respectively. A similar trade‐off (gain in SAc activity, loss in CAc/FAc activity) was also observed by Morley et al. (2013). The results indicate that one or more selected mutation sites are crucial for substrate selectivity. Using CAc as substrate, 297 variants showed less than 0.3‐fold activity compared to N31. For FAc, a similar number of 366 variants were found with less than 0.4‐fold activity compared to the wild type, corresponding to 34.7% (FAc) and 28.1% (CAc) of all variants studied being less active with these two substrates (Figure S10). A total of 88 variants were selected for rescreening towards CAc, FAc, and SAc activity. Two single mutants (Ile29Ala and Ile29Ser), and two double mutants (Ile29Ser, Leu80Val and Ile29Ala, Leu80Ser) displayed high activity towards FAc. The 10 most active variants from the rescreening all contained different triple mutations, with 9 out of 10 containing an OH‐side chain containing amino acid (Ser/Thr) (Table S8). The most active variant identified by the rescreening was a triple mutant with the mutations Ile29Ser, Leu80Ser and Ile93Ala (in the following referred to as N31 SSA), showing a nearly 5‐fold activity increase for SAc in the screening. This, in turn, also resulted in a decrease of activity towards CAc and FAc (Table S9 and Figure S11).

### 2.3 Kinetic characterization of N31 SSA

To determine the effect of the mutations on activity, purified N31 SSA was characterized kinetically. The results clearly showed that this enzyme outperformed all other ancestors for SAc activity at 30°C, with a *V*_max_ of 13.6 ± 1.0 U/mg, whereas N31 only showed an activity of 2.0 ± 0.3 U/mg. Remarkably, the *K*_M_ for SAc decreased slightly in N31 SSA (0.7 ± 0.2 mM) compared to N31 (1.1 ± 0.5 mM). This was also reflected in the *k*_cat_/*K*_M_ of N31 SSA for SAc (6.8 ± 1.3 s^−1^ mM^−1^), which was 11‐times higher than that of N31 for the same substrate. The determined half‐life time of N31 SSA was 1.5 days at 50°C, with the enzyme precipitating after approximately 3 days of incubation (Table S10), indicating that the variant largely maintains the operational stability of N31. The enhanced activity towards SAc (13.6 ± 1.0 U/mg) is at the expense of activity towards FAc (1.6 ± 0.08 U/mg). The N31 SSA mutant also exhibits strong product inhibition towards 4‐VS, with a *K*_*i*_/*K*_*M*_ value of 0.9, and for FAc (*K*_*i*_/*K*_*M*_ of 1.0) (Figure S13). The enhanced activity towards SAc (13.6 ± 1.0 U/mg) is at the expense of activity towards FAc (1.6 ± 0.08 U/mg).

### 2.4 Molecular dynamics (MD) simulations

The experimental results within this study suggest a trade‐off relationship between the activities for FAc and SAc. To fully understand the experimental trade‐off, MD simulations for N31 (PDB ID: 8B30) and N31 SSA were performed, in complex with each of the two substrates, FAc and SAc. Although the orientation that the substrate (independently FAc or SAc) can adopt in the PAD active site has been controversial, the latest accepted mechanism with the lowest energetic barrier (Sheng et al., 2015) proposes the phenolic hydroxy group being placed in an oxyanion hole created by the “twin tyrosines”, Tyr19 and Tyr21, and with Glu72 acting as a general acid–base catalyst (Figure S14), jointly inducing deprotonation of the *p‐hydroxyl* group and formation of the high energy quinone methide intermediate (Rodríguez et al., 2010; Sheng et al., 2015).

**FIGURE 4:**
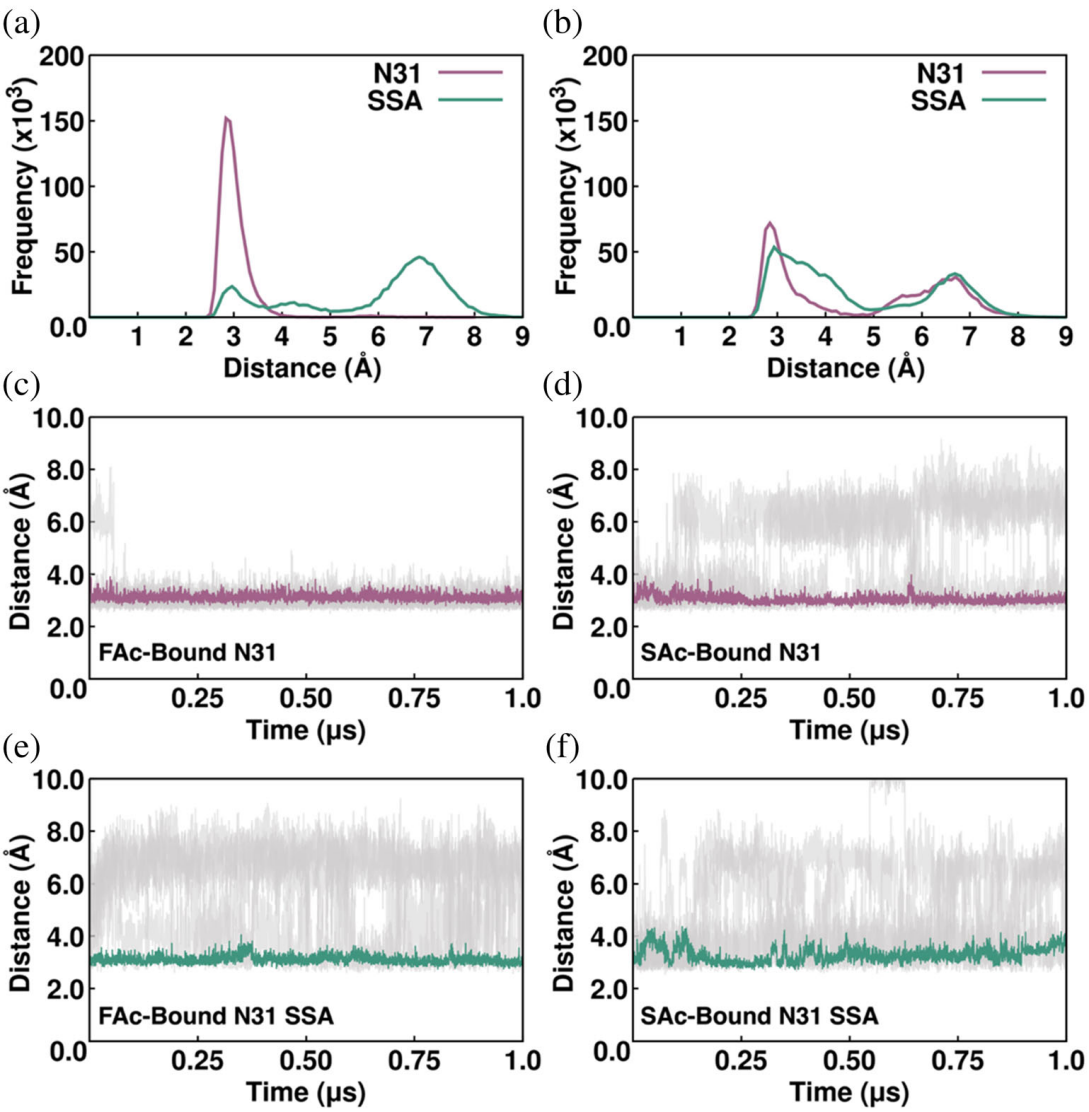
Histograms and time evolution of the distance between Tyr19 and the phenolic oxygen (FAc‐Tyr19/SAc‐Tyr19, Å) during 5 × 1 μs MD simulations of FAc‐ and SAc‐bound N31 and N31 SSA PAD. Shown here is a comparison of histograms of (a) FAc‐bound N31 and N31 SSA, (b) SAc‐bound N31 and N31 SSA, (c, e) time evolution of the FAc‐Tyr19 distance in (c) N31 and (e) N31 SSA, and (d, f) time evolution of the SAc‐Tyr19 distance in (d) N31 and (f) N31 SSA PAD, respectively. In panels (c–f), the shaded region shows the distances sampled by the individual trajectories, and the solid region shows the running average over all replicas. The corresponding data for the phenolic oxygen–Tyr21 distance is shown in Figure S15, and the averages and standard deviations for all catalytic distances to both substrates are shown in Table S7.

Classical MD was performed for the 19 different systems as described in the Section 4. Specifically, 5 × 1 μs simulations were performed on each of unliganded, FAc‐bound and SAc‐bound N31, N31 SSA, as well as of N31 and N31 SSA PAD in complex with the FAc and SAc bound products 4VG and 4VS, respectively, to a total of 95 μs of cumulative simulation time. According to previous mutagenesis studies, the residues Tyr19, Tyr21, Arg49 and Glu72 have been identified as essential for the catalytic activity of PADs (Frank et al., 2012; Gu et al., 2011). Thus, the distances between the ligand (FAc or SAc) and the side chains of these four residues were analyzed during the MD simulations of the N31 and SSA systems (Figures 4, S15 and Table S7), focusing in particular on the twin tyrosine side chains forming the oxyanion hole, which play a crucial role in stabilizing the deprotonated phenolic oxygen of both substrates. (Payer et al., 2017; Sheng & Himo, 2017).

As shown in Figures 4 and S15, and Table S7, the distances between the phenolic oxygen and Tyr21 are focused on a narrow peak on the histogram at ~3 Å, with generally stable time evolution of this distance, and average values ranging from 3.05 to 3.3 Å with overlapping standard deviations, with the least stable interaction being observed in SAc‐bound SSA. In contrast, while a similar peak and stable distribution is observed for the Tyr19‐FAc interaction in FAc‐bound N31, this interaction becomes unstable in SAc N31 and with both substrates in the SSA variant, with two peaks on the histograms, sampling largely unproductive conformations of this hydrogen bond (this situation is slightly better with SAc than in FAc‐bound SSA, with slightly more sampling of productive than nonproductive conformations). In contrast, the interactions between the substrates and Arg49 and Glu72, respectively, are weaker, as shown by larger average distances with smaller standard deviations on those distances in each of these cases (Table S7).

We note that the instability of the Tyr19‐phenolic oxygen interactions is observed due to the reorientation of the substrate into an alternate non‐reactive orientation in the active site stabilized by Ser80 and Thr92, an example of which, in the case of FAc‐bound SSA, is shown in Figure S16. We observe non‐reactive substrate conformations for 55%, 49%, and 5.6% of the simulation time in the FAc‐bound N31 SSA, SAc‐bound N31, and SAc‐bound N31 SSA complexes, based on clustering analysis, performed as described in the Supporting Information. As illustrated by the time evolution of the Tyr19‐ligand distances in Figure 4, this interaction (and thus by extension ligand orientation) is generally stable in FAc‐bound N31, highly unstable in FAc‐bound SSA in all replicates, and marginally less unstable in SAc‐bound SSA N31 than SAc‐bound N31 (see Table S7). Thus, the ability of the substrate to form a stable reactive orientation in the active site and maintain the Tyr19‐phenolic oxygen hydrogen bond appears to be qualitatively related to changes in observed activity and substrate selectivity in these variants. Further, analysis of the conformational space of the Tyr19 and Tyr21 side chains (Figure S17) demonstrates that the conformational space sampled by these two side chains is nearly identical across all four systems, indicating that substrate reorientation is not majorly caused by Tyr sidechain flexibility. We note, however, that SAc‐bound N31 samples an unproductive conformation of Tyr19 as a minor conformer due to a steric clash between this side chain and the SAc OMe group, which is largely absent in the SSA variant, contributing to increased activity in this variant towards SAc. Based on these observations, the ~5 kcal/mol strength of an OH:O hydrogen bond can, to a large extent, account for the observed differences in selectivity between the variants (David et al., 2017).

**FIGURE 5:**
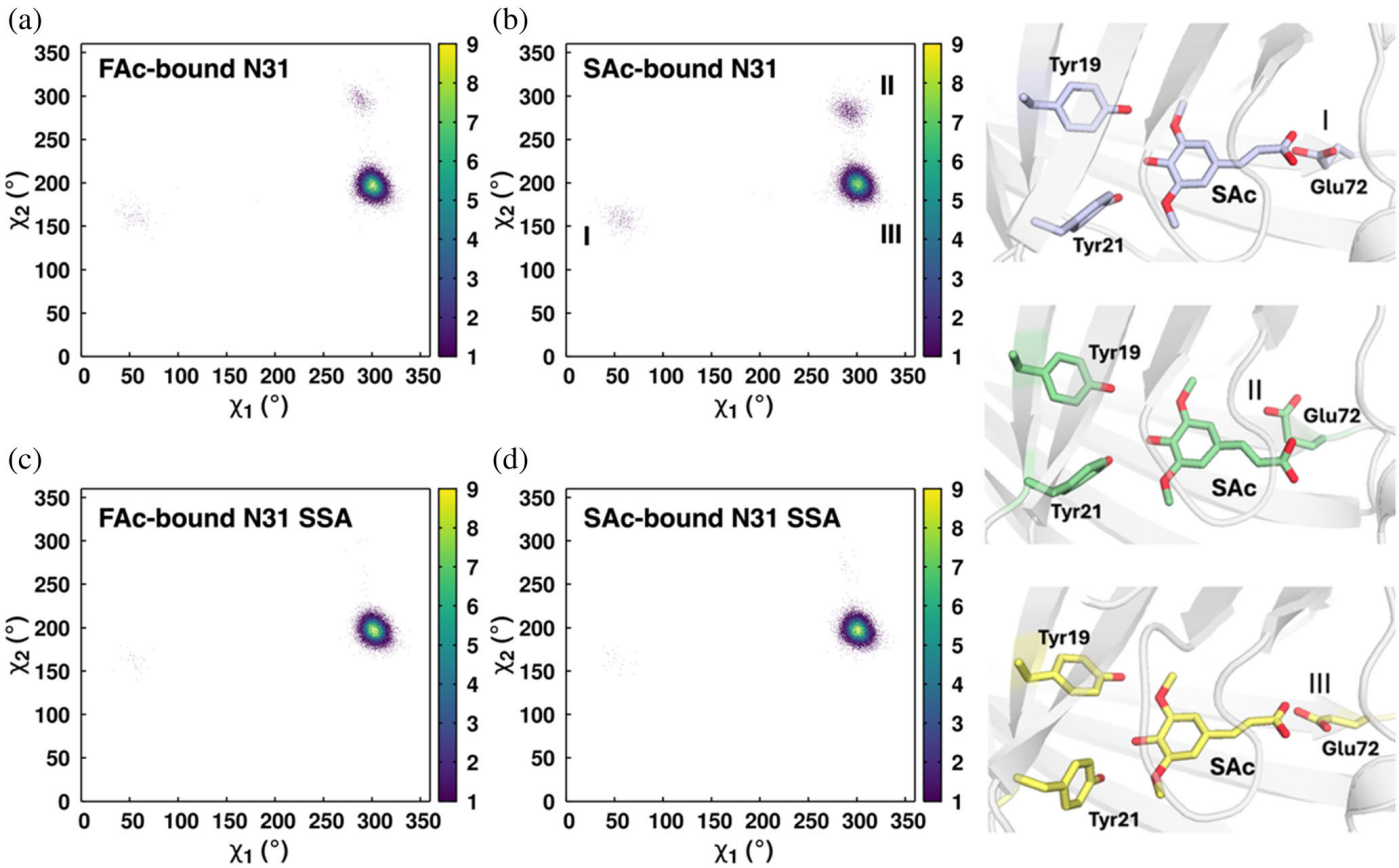
Joint distribution of the conformational space sampled by the χ_1_ and χ_2_ dihedral angles of the Glu72 side chain. Data is based on analysis of 5 × 1 μs simulations of each of the (a) FAc‐bound N31, (b) SAc‐bound N31, (c) FAc‐bound N31 SSA and (d) SAc‐bound N31 SSA, with data collected every 200 ps. The population density is represented by colors scaled in the color bars. The right panel shows representative conformations of each of the three Glu72 conformations, based on clustering analysis of Glu72 in the SAc‐bound N31 system performed as described in the Supplementary Methods, which results in three distinct metastable conformations.

Beyond the oxyanion hole, our simulations additionally show flexibility in the side chain of Glu72, which can further account for the improvement in activity of N31 SSA over N31 towards SAc. Specifically, as shown in Figure 5, in our simulations of both FAc and SAc‐bound N31 (panels A and B), Glu72 samples a range of metastable side‐chain conformations, of which two (Conformations I and III) are very similar and both plausibly catalytic, and one (Conformation II), is not catalytic, with the Glu72 side chain pointing out of the active site. The respective populations of each conformation in simulations of FAc‐bound N31 are Conformation I (0.5%), Conformation II (1.8%) and Conformation III (97.7%) of simulation time, and in SAc‐bound N31 are Conformation I (1.2%), Conformation II (5.4%) and Conformation III (93.4%) of simulation time. The mutations introduced in the N31 SSA variant lead to conformational enrichment of the catalytically acceptable conformation III (Glu72 pointing into the active site), and the other conformations observed in simulations of both FAc and SAc‐bound N31 are largely not sampled (Conformation I is sampled for at least 0.1% for both FAc and SAc of the simulation time, and the non‐catalytic Conformation II is only sampled in 0.1% of the simulation time for SAc and less than 0.1% for FAc). Visual analysis of our simulation trajectories indicates that the non‐reactive conformation of Glu72 is generated by a conformational change of Ile41, which allows the substrate (both FAc and SAc) to move in the active site, in turn displacing the side chain of Glu72 from a catalytically favorable conformation. The Ile93Ala mutation in the SSA variant creates space in the active site pocket, which reduces the need for substrate reorientation in the case of SAc, accommodating the additional OMe on this substrate, and likely contributing to the enhanced activity of this variant towards substrate SAc.

From a general perspective, the substrate conformational changes detected along the MD simulations are a direct consequence of the differences in pocket volume between N31 and the engineered N31 SSA. Analysis of the unliganded active sites of N31 and N31 SSA during our MD simulations (snapshots taken every 200 ps of 5 × 1 μs trajectories) using MDPocket (Schmidtke et al., 2011) indicated pocket volumes of 224.5 ± 80.6 Å^3^ for N31 PAD, and 304.3 ± 96.9 Å^3^ for the engineered SSA variant, compared to volumes of 170.9 and 194.4 Å^3^ for FAc and SAc, respectively calculated using RDKit (https://www.rdkit.org). While both active sites are large enough to accommodate both substrates, the larger and (based on standard deviation on the pocket volume) more flexible SSA active site can more easily accommodate the larger SAc substrate with its additional OMe group, while simultaneously rendering the smaller FAc substrate less stable in the binding pocket. Similar analysis of active site volumes in the unliganded Ile29Ser, Leu80Ser and Ile93Ala variants yield volumes of 210.1 ± 100.0, 176.2 ± 95.6 and 327.1 ± 84.5 Å^3^, respectively, indicating that the Ile93Ala substitution alone is sufficient to create most of this additional active site space observed in the N31 SSA variant, despite this Ile93Ala single variant being inactive towards both FAc and SAc (Figure S1).

In order to better understand why the Ile93Ala variant is inactive towards both FAc and SAc, despite this substitution playing an important role in increasing the activity of N31 SSA towards SAc, we performed an analysis of hydrogen bonding networks to the ligand, as well as water networks. Table S8 shows an overview of the average hydrogen bonds from solvent to substrates FAc and SAc, and to Glu72, an increase of either of which would be expected to be detrimental to catalytic activity. Overall, the active site of PAD is buried, hydrophobic, and shielded from solvent, and the limited solvent‐ligand interactions observed are with the carboxylate group of the substrate. Unsurprisingly, the Ile93Ala substitution increases active site volume, adding additional water to the active site, and increasing the solvent exposure of the substrate CO_2_ group (thus stabilizing it and making it less likely to cleave) and/or the glutamic acid side chain (making it less likely to abstract a proton).

Analysis of the occupancy of protein‐ligand hydrogen bonding interactions (Figures S18 and S19) indicates that the overall protein‐ligand interaction networks are similar between systems and ligands. However, in the SSA variant (Figure S18), Ser80 is able to form an additional stabilizing hydrogen bond with the phenolic oxygen of the substrate, with this hydrogen bond being formed 25.8 and 55.4% of simulation time in simulations of SSA‐bound FAc and SAc, respectively, likely offsetting the detrimental impact of the space created by the Ile93Ala substitution on the solvent exposure of the ligand and Glu72 side chains by stabilizing the phenolic oxygen. Interestingly, this interaction is not observed in the single Leu80Ser variant (this side chain does not interact with FAc and interacts with the OMe group oxygen of SAc rather than the phenolic oxygen), likely due to the steric bulk of Ile93 blocking this interaction in the single variant.

**FIGURE 6:**
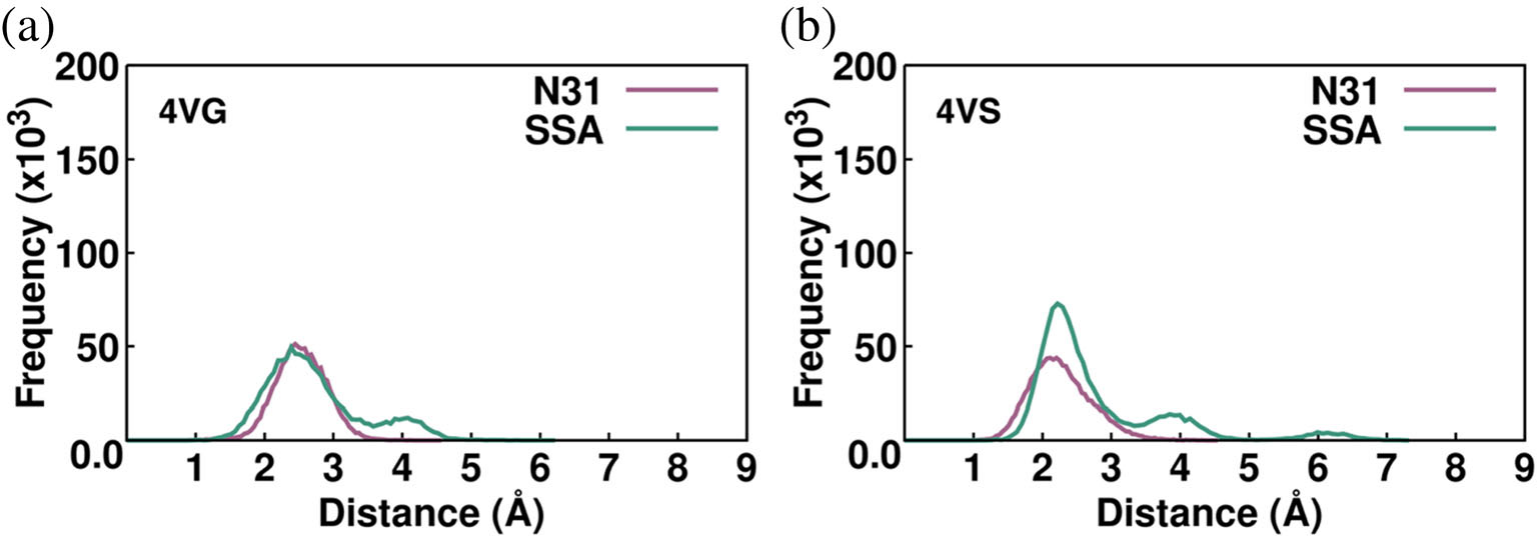
Histogram of the distance between the ligand COM and active site COM during 5 × 1 μs MD simulations of 4VG‐ and 4VS‐bound N31 and N31 SSA PAD. (a) 4VG‐bound N31 and N31 SSA, (b) 4VS‐bound N31 and N31 SSA.

Finally, we performed simulations of N31 and N31 SSA in complex with the FAc and SAc products 4‐VG and 4‐VS, respectively, to probe the origins of the product inhibition shown towards this variant (Figure 2 and Table S9). Docking revealed no other binding sites for the products other than the active site. The stability of the product systems was determined by distance analysis of the center of mass (COM) for the ligand and active site pocket (Figure 6) and the distance of the ligand to the previously discussed catalytic residues.

In particular, we focused on the interaction between the oxyanion hole forming tyrosine side chains Tyr19 and Tyr21, and the deprotonated phenolic oxygen on each product (Figures S20 and S21). While oxyanion holes are important for transition state stabilization, they can cause product trapping when the product has a similar charge distribution and shape to the transition state (see e.g., Presnell et al., 1998 for an example of oxyanion‐mediated inhibition of serine proteases). Our analysis shows that, in contrast to the substrate (Figures 4 and S15), both products are held in a very stable oxyanion hole formed by these two residues, effectively trapping the product in the active site (Figures S20 and S21).

Overall, our simulations indicate an interplay between the shape of the binding pocket, the resulting substrate and active site plasticity, the formation of key interactions (in particular with the oxyanion hole), and conformational enrichment of productive conformations of Glu72 in driving the selectivity and product inhibition of the N31 ancestral PAD and its engineered variants.

## 3 CONCLUSIONS

In this study, we described the engineering and characterization of a thermally stable SAc‐accepting PAD variant. Starting from four thermally stable ancestors, the most stable ancestor was identified to be PAD N31 with a half‐life of 3.65 days (at 50°C), while the half‐life of *Bs*PAD under the same conditions is >1 min. Since no ancestor was found to convert SAc sufficiently, PAD N31 was engineered to broaden the substrate scope of this enzyme. The screening of the combinatorial library based on N31 identified an Ile29Ser, Leu80Ser, Ile93Ala triple mutation (N31 SSA) that expanded the substrate scope with an almost 11‐fold increase in the catalytic efficiency for SAc, while the specific SAc activity increased from 2 to 13.6 U/mg. A comparison between PAD N31 and N31 SSA shows a trade‐off relationship of the substrates. This also reflects in the opposite behavior of N31 and N31 SSA for FAc and SAc, as illustrated by their kinetic behavior and product inhibition. The experimental results were corroborated by MD simulations, which showed that the Ile93Ala mutation is crucial in increasing the active site cavity, due to the small size of Ala compared to the original Ile. We plan to intensify the process using the thermally stable, SAc‐accepting PAD variant in deep eutectic solvents. Product inhibition by styrene may pose a major challenge for process scale‐up. To overcome this limitation, future work will focus on the implementation of continuous in situ product removal systems or on targeted engineering approaches aimed at enhancing product tolerance and process robustness (Meyer et al., 2022; Pesci et al., 2017).

## 4 EXPERIMENTAL SECTION

### 4.1 Combinatorial library preparation

Fragments were created via PCR using mutagenic or Gibson assembly. Afterwards, the library was screened with a photometric assay (SI).

### 4.2 Cultivation of *E. coli* BL21 containing pET28a_PAD plasmids for purification

For the overnight culture (ONC) 4 mL lysogeny broth medium (10 g/L tryptone, 10 g/L NaCl, 5 g/L yeast extract containing 40 μg/μL kanamycin) was inoculated with a single colony and incubated overnight at 37°C and 130 rpm (orbit diameter 25 mm) for 16 h. Afterwards, the entire ONC was transferred into 200 mL terrific broth (TB) medium (12 g/L tryptone, 24 g/L yeast extract, 2.31 g/L KH_2_PO_4_, 12.54 g/L K_2_HPO_4_, 40 μg/μL kanamycin) with autoinduction medium (0.5 g glucose, 2 g/L α‐lactose) in a 1 L baffled flask. The main culture was incubated at 37°C and 120 rpm (orbit diameter 25 mm) until an optical density at 600 nm (OD_600_) of about 0.5–0.6 was reached (after ~3 h). The culture was incubated at 20°C and 120 rpm (orbit diameter 25 mm) for about 24 h. Final OD_600_ of around 20–30 was obtained. The cells were harvested by centrifugation at 2400 × g at 4°C for 15 min and washed twice with 20 mL potassium phosphate buffer (pH 6, 50 mM) at 2400×*g* and 15 min. After discarding the supernatant, the cell pellet was stored at −20°C until further use.

### 4.3 Purification of PAD

The cell pellet was resuspended in His‐tag purification buffer (20 mM Tris–HCl, 300 mM NaCl, 30 mM imidazole, pH 7.4) and the cells were disrupted by sonication (output control: 50% duty cycle: 5). After centrifugation at 71200 × g for 45 min, the supernatant was sterile filtered. The protein was then purified by Ni‐affinity chromatography with a Ni Sepharose 6 Fast Flow column with a bed volume of 10 mL (GE Healthcare; Germany) attached to a fast protein liquid chromatography system (ÄKTAprime plus, Cytiva, Germany). After elution, the buffer was exchanged to potassium phosphate buffer (pH 6, 50 mM) using PD‐10 desalting columns (GE Healthcare, Germany). The purified enzymes were then stored in small aliquots with 20% (v/v) glycerol at −20°C. The protein concentration was determined by measuring the absorbance at 280 on a NanoDrop spectrophotometer (Thermo Fisher Scientific, USA). Protein purity was confirmed with SDS‐PAGE (Figure S1).

### 4.4 Activity measurement via photometric assay

The photometric assay was performed in potassium phosphate buffer (pH 6, 50 mM) with a final volume of 100 μL containing ca. 1 μg enzyme and 0 to 4 mM FAc (FAc stock: 200 mM in DMSO). The reaction was carried out at 30 and 50°C and followed for 5 min at 344 nm on a FLUOstar Omega multidetection microplate reader (BMG LABTECH GmbH, Germany). For fitting the data, the standard Michaelis–Menten model was used:

(1) $$y=\frac{V_{\max}\cdot \left[S\right]}{K_{M}+\left[S\right]}$$

Standard deviations were excluded from the model. Similarly, the reaction was carried out with SAc as the substrate by adding ca. 100 μg enzyme and 0 to 4 mM SAc (SAc stock: 200 mM in DMSO). Substrate depletion was followed via absorption at 348 nm for 5 min. The *k*_cat_ was calculated with the following Equation (2):

(2) $$k_{\mathrm{cat}}=\frac{\text{spec}.\;\text{activity}\;\left(\frac{U}{\mathrm{mg}}\right)\cdot \mathrm{MW}\;\left(\frac{g}{\mathrm{mol}}\right)}{60\;000}$$

Time conversion: 1 Unit (U) of enzyme activity is defined as the amount of enzyme that catalyzes the conversion of 1 μmol of substrate per minute. To obtain the catalytic constant in the standard unit of seconds (s^−1^), a factor of 60 is required. Mass conversion: The specific activity is given in U per milligram (U/mg), while the molecular weight (MW) is provided in grams per mole (g/mol). To convert milligrams to grams, a factor of 1000 is used. This equals a conversion factor of 1/60,000.

### 4.5 Product inhibition measurement via photometric assay

To evaluate product inhibition, 100 μg of enzyme was incubated in 50 mM phosphate buffer (pH 6) with varying initial concentrations of FAc (0–4 mM) in a volume of 100 μL at 30°C. The reaction was carried out until the initial FAc was fully converted. Subsequently, 4 mM of fresh FAc was added, and the degradation was monitored photometrically for 5 min at 344 nm. An identical, independent assay was performed for SAc (using a 200 mM stock in DMSO), where varying initial concentrations of SAc were fully converted to 4‐VS prior to the addition of 4 mM fresh SAc, with absorption measured at 348 nm. Fitting to the below‐displayed equation was performed without standard deviations. The GRG Nonlinear Solving Method for nonlinear optimization was used (Equation 3).

(3) $$v=\frac{v_{\max}\cdot \left[S\right]}{K_{M,S}\left(1+\frac{\left[P\right]}{K_{i,P}}\right)+\left[S\right]}$$

### 4.6 Half‐life measurements

Aliquots of 50 μL were incubated at 50°C. Samples were withdrawn periodically and activity measurements were carried out at 30°C with 4 mM FAc (FAc stock: 200 mM in DMSO) for the ancestors and SAc (SAc stock: 200 mM in DMSO) for SSA in a photometer (FLUOstar Omega multidetection microplate reader, BMG LABTECH GmbH, Germany). Absorption was followed at 344 nm for the ancestors and 348 nm for SSA.

### 4.7 Molecular dynamics simulations and analysis

Molecular dynamics simulations for N31, SSA, and each of the Ile29Ser, Leu80Ser, and Ile93Ala single variants were performed of each system in complex with substrates FAc and SAc, with additional simulations of N31 and SSA in complex with the 4‐VG and 4‐VS products, respectively. In the case of N31, the available crystallographic structure was used as a starting point for the simulation (PDB ID: 8B30) (Myrtollari et al., 2024). The structure of the SSA variant and single variants were generated by inserting the corresponding mutations (Ile29Ser, Leu80Ser, and Ile93Ala) into the N31 crystal structure, using the PyMOL “Mutagenesis” function (DeLano, 2021). Rotamers were selected from the Backbone‐Dependent Rotamer library, such as to eliminate structural clashes (Dunbrack Jr & Cohen, 1997). The resulting structures were then prepared for simulations and equilibrated following a standard equilibration procedure, as described in detail in the Data S1 (Supporting Methods). Once equilibrated, five 1 μs production runs were simulated for each system in an NPT ensemble (1 atm pressure and 300 K). In total, we obtained 5 μs cumulative simulation time per system. The convergence of heavy atom root mean square deviations (RMSD) in all simulations is shown in Figures S22 and S23. For all the simulations, SHAKE (Kräutler et al., 2001) was applied to constrain all bonds containing hydrogen atoms, and a 4 fs time step was used in all simulations (Hopkins et al., 2015). Langevin temperature control (collision frequency of 1 ps^−1^) and a Berendsen barostat (Berendsen et al., 1984) (pressure relaxation time of 1 ps) were employed along the dynamics. All MD simulations in this study were performed using the HIP‐accelerated version of Amber24 (Case et al., 2023) using the ff19SB force field (Tian et al., 2020) and the OPC water model (Izadi et al., 2014) further details of simulation setup, equilibration and analysis are provided as Data S1 (Supporting Methods), and a data package containing simulation starting structures, restart files from equilibration, snapshots from trajectories, representative input files and any nonstandard simulation parameters have been uploaded to Zenodo, DOI: https://doi.org/10.5281/zenodo.15638241.

## AUTHOR CONTRIBUTIONS

**Robert Kourist:** Investigation; writing – review and editing; supervision; conceptualization; project administration. **Aron Wanz:** Investigation. **Daniel Kracher:** Writing – review and editing; writing – original draft; validation. **Selin Kara:** Investigation; writing – review and editing; writing – original draft; supervision. **Kristin K. F. Bauer:** Conceptualization; writing – original draft; methodology; investigation. **Shina C. L. Kamerlin:** Investigation; supervision; writing – review and editing. **Tobias Schöngassner:** Investigation. **Lars‐Erik Meyer:** Supervision. **Eva Vos:** Investigation; writing – original draft. **Sonja Vaupel:** Investigation; writing – original draft; data curation. **Jacques Gay:** Investigation.

## FUNDING INFORMATION

This research was funded in whole or in part by the Austrian Science Fund (FWF) [10.55776/doc.46, 10.55776/P34280]. Selin Kara thanks the Ministry for Science and Culture for Lower Saxony for the Holen & Halten starting grant (grant no 12.5‐76251‐17‐9/20). Shina C. L. Kamerlin thanks the Georgia Research Alliance, the Vasser Wooley Foundation, and Georgia Tech for support. Jacques Gay thanks the U.S. Department of Education for their generous support of this research via the Georgia Institute of Technology Biochemistry and Biophysics Graduate Assistance in Areas of National Need (GAANN) Grant, Award #P200A210014. Computational resources were provided by the National Academic Infrastructure for Supercomputing in Sweden (NAISS), funded by the Swedish Research Council. We further thank UPPMAX, Uppsala University, for access to the Pelle cluster. Daniel Kracher thanks the BiotechMed‐Graz Young Researcher Group program.

## CONFLICT OF INTEREST STATEMENT

The authors declare no conflict of interest.

## Supporting information


**FIGURE S1.** Purification of PAD variants. (a) wild‐type PAD N31. (b) PAD N31 S–. (c) PAD N31 ‐S‐. (d) PAD N31 –A. (e) PAD N31 SS‐. (f) PAD N31 S‐A. (g) PAD N31 SSA. (h) PAD N2. (i) PAD N4. (j) PAD N80. (k) PAD N100. L: PageRuler Prestained Protein Ladder, 10 to 180 kDa (ThermoFisher Scientific) for (a–g), PL00001 Prestained Protein Marker (10–180 kDa), Proteintech, D: debris, CFE: soluble CFE fraction, FT: flowthrough, W: wash, E: elution, WE: wash after elution and RE: Rebuffered elution fraction. Expected size: 21.9 kDa.
**FIGURE S2.** Specific activity of PAD ancestors towards FAc at 30°C with various concentrations—Michaelis–Menten kinetics analysis. The photometric assay was performed in potassium phosphate buffer (50 mM, pH 6) with a final volume of 100 μL containing 12.5 μg enzyme and 0 to 4 mM FAc (FAc stock: 200 mM in DMSO). The reaction was carried out at 30°C and observed for 5 min at 344 nm. Fitting was performed in Origin with the standard Michaelis–Menten fit (see also Material and Methods part for further details). GRG Nonlinear Solving Method for nonlinear optimization was used.
**FIGURE S3.** Specific activity of PAD ancestors towards FAc at 50°C with various concentrations—Michaelis–Menten kinetics analysis. (a) N2, (b) N4, (c) N80, (d) N100, (e) N31. The photometric assay was performed in potassium phosphate buffer (50 mM, pH 6) with a final volume of 100 μL containing 12.5 μg enzyme and 0 to 4 mM FAc (FAc stock: 200 mM in DMSO). The reaction was carried out at 50°C and observed for 5 min at 344 nm. Fitting was performed in Origin with the standard Michaelis Menten fit (see also Materials and Methods part for further details). GRG Nonlinear Solving Method for nonlinear optimization was used.
**FIGURE S4.** Specific activity of PAD ancestors towards SAc at 30°C with various concentrations—Michaelis–Menten kinetics analysis. (a) N2, (b) N4, (c) N80, (d) N100, (e) N31. The photometric assay was performed in potassium phosphate buffer (50 mM, pH 6) with a final volume of 100 μL containing 25 μg enzyme and 0 to 4 mM SAc (SAc stock: 200 mM in DMSO). The reaction was carried out at 30°C and observed for 5 min at 348 nm. Fitting was performed in Origin with the standard Michaelis–Menten fit (see also Materials and Methods part for further details). GRG Nonlinear Solving Method for nonlinear optimization was used.
**FIGURE S5.** Specific activity of PAD ancestors towards SAc at 50°C with various concentrations—Michaelis–Menten kinetics analysis. (a) N2, (b) N4, (c) N80, (d) N100, (e) N31. The photometric assay was performed in potassium phosphate buffer (50 mM, pH 6) with a final volume of 100 μL containing 25 μg enzyme and 0 to 4 mM SAc (SAc stock: 200 mM in DMSO). The reaction was carried out at 50°C and observed for 5 min at 348 nm. Fitting was performed in Origin with the standard Michaelis–Menten fit (see also Materials and Methods part for further details). GRG Nonlinear Solving Method for nonlinear optimization was used.
**FIGURE S6.** Half‐life time measurements for five PAD ancestors and SSA mutant. Aliquots of 50 μL were incubated at 50°C. Activity measurements were carried out at 30°C with 4 mM FAc, except SSA was measured with SAc in a photometer. Absorption was followed at 344 and 348 nm, respectively.
**TABLE S1.** Summary of stability data of the ancestors N2, N4, M80, N100 and N31.
**FIGURE S7.** Specific activity of PAD ancestors towards FAc with various concentrations of 4‐VG—Product inhibition analysis. The photometric assay was performed in potassium phosphate buffer (50 mM, pH 6) with a final volume of 100 μL containing 12.5 μg enzyme and 0 to 4 mM 4‐VG (FAc stock: 200 mM in DMSO). The reaction was carried out at 30°C and observed for 5 min at 344 nm (see also Materials and Methods part for further details).
**TABLE S2.** Summary of kinetic data for product inhibition FAc at 30°C. GRG Nonlinear Solving Method for nonlinear optimization was used.
**FIGURE S8.** Specific activity of PAD ancestors towards SAc with various concentrations of 4‐VS—Product inhibition analysis. The photometric assay was performed in potassium phosphate buffer (50 mM, pH 6) with a final volume of 100 μL containing ca. 25 μg enzyme and 0 to 4 mM 4‐VS (SAc stock: 200 mM in DMSO). The reaction was carried out at 30°C and observed for 5 min at 348 nm.
**TABLE S3.** Summary of kinetic data for product inhibition SAc at 30°C. GRG Nonlinear Solving Method for nonlinear optimization was used.
**FIGURE S9.** Absolute activities (as Δ A.U. min‐1) of the rescreening library towards (a) 1.5 mM CAc, (b) 1.5 mM FAc and (c) 1.5 mM SAc. Reaction conditions: 30°C, 100 μL total volume, 1.5 mM substrate in potassium phosphate buffer (50 mM, pH 6). Reactions were performed using a cell‐free extract (CFE) total protein concentration of 10 μg/mL for CAc and FAc, and 5500 μg/mL for SAc. *N* = 3. The red lines indicate ±3× standard deviation of the mean pET28a (empty vector) control to indicate the noise range for each substrate.
**FIGURE S10.** Histogram of the fold change activity (determined as the Δabsorbance over time) of the combinatorial PAD N31 library (1056 colonies) towards three different substrates. The fold changes were calculated plate‐wise to the wt controls present on each plate. The red lines indicate the mean ± standard deviation for the wt controls. The number of colonies within and outside of these ranges is displayed at the right‐hand corner of each histogram. (a) 1.5 mM CAc, (b) 1.5 mM FAc and (c) 1.5 mM SAc. Reaction conditions: 30°C, 100 μL total volume, 1.5 mM substrate in potassium phosphate buffer (50 mM, pH 6). CFE protein concentrations used: 10 μg/mL for CAc & FAc, 5500 μg/mL for SAc.
**TABLE S4.** Activities and sequences (via Sanger sequencing) of the 10 most active colonies from the rescreening process for each substrate tested.
**TABLE S5.** Absolute activities (as Δ A.U.min‐1) of selected variants from the rescreening of the combinatorial PAD N31 library towards three tested substrates (CAc, FAc and SAc). Reaction conditions: 30°C, 100 μL total volume, 1.5 mM substrate in potassium phosphate buffer (50 mM, pH 6). Reactions were performed using a cell‐free extract (CFE) total protein concentration of 10 μg/mL for CAc and FAc, and 5500 μg/mL for SAc. *N* = 3.
**FIGURE S11.** Comparison of selected PAD N31 variants from the rescreening on their absolute activity towards different substrates. *N* = 12 for PAD N31, *N* = 3 for mutants and *N* = 6 for pET28a. Reaction conditions: 30°C, 100 μL total volume, 1.5 mM substrate in potassium phosphate buffer (50 mM, pH 6). CFE protein concentrations: 10 μg/mL for CAc & FAc, 5500 μg/mL for SAc. Abbreviations: N31 (N31 wild type), N31 SSA (N31 Ile29Ser, Leu80Ser, Ile93Ala); N31 SS‐ (N31 Ile29Ser, Leu80Ser); N31 S– (N31 Ile29Ser).
**FIGURE S12.** Specific activity of PAD ancestors towards FAc and SAc at 30°C with various concentrations. The reactions were carried out for (a) FAc at 30°C, (b) SAc at 30°C. The photometric assay was performed in potassium phosphate buffer (50 mM, pH 6) with a final volume of 100 μL containing ca.12.5 and 25 μg enzyme and 0 to 4 mM SAc (SAc stock: 200 mM in DMSO). The reaction was carried out at 30°C and observed for 5 min at 348 nm. Fitting was performed in Origin with the standard Michaelis–Menten fit (see also Materials and Methods part for further details).
**FIGURE S13.** Specific activity of N31 SSA towards FAc and SAc with various concentrations of the respective product. The photometric assay was performed in potassium phosphate buffer (pH 6, 50 mM) with a final volume of 100 μL containing ca. 25 μg enzyme and 0 to 4 mM 4‐VG/4‐VS (FAc: 200 mM in DMSO; SAc stock: 200 mM in DMSO). The reaction was carried out at 30°C and observed for 5 min at 344 and 348 nm, respectively.
**TABLE S6.** Summary of kinetic and stability data for N31 SSA at 30°C obtained with competitive inhibition curve fit in origin.
**FIGURE S14.** Most likely binding orientation of the substrate FAc in the phenolic acid decarboxylase enzyme (Payer et al., 2017; Sheng et al., 2015). The phenolic hydroxy group is assumed to be deprotonated upon binding to the PAD active site, and stabilized by an oxyanion hole formed by the side chains of Tyr19 and Tyr21, with Glu72 acting as a general acid–base catalyst.
**FIGURE S15.** Histograms and time evolution of the distance between Tyr21 and the phenolic oxygen (FAc‐Tyr19/SAc‐Tyr19, Å) during 5 × 500 ns MD simulations of FAc‐ and SAc‐bound N31 and N31 SSA PAD. Shown here are a comparison of histograms of (a) FAc‐bound N31 and N31 SSA, (b) SAc bound N31 and N31 SSA, (c, e) time evolution of the FAc‐Tyr19 distance in (c) N31 and (e) N31 SSA, and (d, f) time evolution of the SAc‐Tyr19 distance in (d) N31 and (f) N31 SSA PAD, respectively. In panels (c–f), the shaded region shows the distances sampled by the individual trajectories, and the solid region shows the running average over all replicas. The corresponding data for the phenolic oxygen–Tyr21 distance is shown in Figure 4, and the averages and standard deviations for all catalytic distances to both substrates are shown in Table S7.
**FIGURE S16.** Illustration of the interactions between the ligand and Ser80 and Thr92 in FAc‐bound SSA system. The interaction allows the stabilization of a non‐reactive conformation of the substrate in the active site, which we observe for 55% of simulation time, based on conformational clustering analysis performed as described in the Supplementary Methods. The cyan dotted lines represent the polar interactions. The yellow dotted lines represent the distances in Å, taken from a representative snapshot collected from ligand clustering analysis.
**FIGURE S17.** Joint distribution of the conformational space sampled by the χ^1^ and χ^2^ dihedral angles of the Tyr19 (a–d) and Tyr21 (e–h) side chains, during 5 × 100 μs simulations of the (a, e) FAc‐bound N31, (b, f) SAc‐bound N31, (c, g) FAc‐bound N31 SSA and (d, h) SAc‐bound N31 SSA. Data was collected every 200 ps.
**FIGURE S18.** Protein‐ligand hydrogen bonding interactions in FAc‐ and SAc‐bound N31 and N31 SSA. Shown here are hydrogen bonds between key active site residues and the respective ligand that are present at ^3^20% simulation time over 5 × 1 μs simulations of each system, calculated using CPPTRAJ (Roe and Cheatham 2013) and visualized with PyMOL (DeLano, 2021). Shown here are data for simulations of (a) FAc‐bound N31, (b) SAc‐bound N31, (c) FAc‐bound SSA and (d) SAc‐bound SSA, where line weight and color (yellow: lowest; red: strongest) are indicative of hydrogen bond persistence.
**FIGURE S19.** Protein‐ligand hydrogen bonding interactions in FAc‐ and SAc‐bound N31 single variants. Shown here are hydrogen bonds between key active site residues and the respective ligand that are present at ^3^20% simulation time over 5 × 1 μs simulations of each system, calculated using CPPTRAJ (Roe and Cheatham 2013) and visualized with PyMOL (DeLano, 2021). Shown here are data for simulations of (a) FAc‐bound Ile29Ser, (b) SAc‐bound Ile29Ser, (c) FAc‐bound Leu80Ser, (d) SAc bound Leu80Ser, (e) FAc‐bound Ile93Ala and (f) SAc‐bound Ile93Ala, where line weight and color (yellow: lowest; red: strongest) are indicative of hydrogen bond persistence.
**FIGURE S20.** Histograms and time evolution of the distance between Tyr19 and the phenolic oxygen (4VG‐Tyr19/4VS‐Tyr19, Å) during 5 × 1 μs MD simulations of 4VG‐ and 4VS‐bound N31 and N31 SSA PAD. Shown here are a comparison of histograms of (a) 4VG‐bound N31 and N31 SSA, (b) 4VS bound N31 and N31 SSA, (c, e) time evolution of the 4VG‐Tyr19 distance in (c) N31 and (e) N31 SSA, and (d, f) time evolution of the 4VS‐Tyr19 distance in (d) N31 and (f) N31 SSA PAD, respectively. In panels (c–f), the shaded region shows the distances sampled by the individual trajectories, and the solid region shows the running average over all replicas. The corresponding data for the phenolic oxygen.
**FIGURE S21.** Histograms and time evolution of the distance between Tyr21 and the phenolic oxygen (4VG‐Tyr21/4VS‐Tyr21, Å) during 5 × 1 μs MD simulations of 4VG‐ and 4VS‐bound N31 and N31 SSA PAD. Shown here are a comparison of histograms of (a) 4VG‐bound N31 and N31 SSA, (b) 4VS bound N31 and N31 SSA, (c, e) time evolution of the 4VG‐Tyr21 distance in (c) N31 and (e) N31 SSA, and (d, f) time evolution of the 4VS‐Tyr21 distance in (d) N31 and (f) N31 SSA PAD, respectively. In panels (c–f), the shaded region shows the distances sampled by the individual trajectories, and the solid region shows the running average over all replicas. The corresponding data for the phenolic oxygen.
**FIGURE S22.** Root mean square deviations (RMSD, Å) of the Cα‐atoms during MD simulations of wild type (N31) and mutated (SSA) phenolic acid decarboxylases. (a) FAc‐bound N31, (b) SAc‐bound N31, (c) FAc‐bound SSA, (d) SAc‐bound SSA, (e) FAc‐bound N31 I29S, (f) SAc‐bound N31 I29S, (g) FAc bound N31 L80S, (h) SAc‐bound N31 L80S, (i) FAc‐bound N31 I93A and (j) SAc‐bound N31 I93A. Data was collected every 200 ps for 500 ns from 5 replicas of 1 μs length each to test convergence. The gray lines show the 5 individual runs, whilst the color solid line shows a rolling average RMSD from all 5 replicas for each system. Tail residues of the monomers (residues 151–157 and 309–317) were removed due to the high flexibility of those regions.
**FIGURE S23.** Root mean square deviations (RMSD, Å) of the Cα‐atoms during MD simulations of wild type (N31) and mutated (SSA) phenolic acid decarboxylases. (a) 4VG‐bound N31, (b) 4VS‐bound N31, (c) 4VG‐bound SSA, (d) 4VS‐bound SSA, (e) Unliganded N31, (f) Unliganded N31 SSA, (g) Unliganded N31 I29S, (h) Unliganded N31 L80S and (i) Unliganded N31 I93A. Data was collected every 200 ps for 500 ns from 5 replicas of 1 μs length each to test convergence. The gray lines show the 5 individual runs, whilst the color solid line shows a rolling average RMSD from all 5 replicas for each system. Tail residues of the monomers (residues 151–157 and 309–317) were removed due to the high flexibility of those regions.
**TABLE S7.** Average values and standard deviations of the key catalytic distances between the ligands FAc and SAc, and N31 and N31 SSA PAD.^a^
**TABLE S8.** Number of solvent‐ligand and solvent‐Glu72 hydrogen bonds in simulations of N31, N31 SSA, and the N31 single variants.^a^
**TABLE S9.** Average values and standard deviations of the center‐of‐mass (COM) of the ligands 4‐VG and 4‐VS distances to the center‐of‐mass N31 and N31 SSA PAD as well as twin‐tyrosine distances between 4‐VG and 4‐VS, and N31 and N31 SSA PAD. a
**TABLE S10.** Non‐standard force field parameters used to describe the substrate ferulic acid (FAc) in our conventional molecular dynamics simulations.^a^
**TABLE S11.** Non‐standard force field parameters used to describe the substrate synaptic acid (SAc) in our conventional molecular dynamics simulations.^a^
**TABLE S12.** Non‐standard force field parameters used to describe the substrate synaptic acid (4‐VG) in our conventional molecular dynamics simulations.^a^
**TABLE S13.** Non‐standard force field parameters used to describe the substrate synaptic acid (4‐VS) in our conventional molecular dynamics simulations.^a^.
**DATA S1.** The supplementary information associated with this article is provided as a single PDF file. It includes detailed descriptions of experimental procedures, supporting results, figures, and tables that complement the main text. It contains enzyme preparation methods, Michaelis–Menten kinetics data, stability and inhibition analyses, combinatorial library screening results, molecular dynamics (MD) simulation protocols, and additional references. Simulation starting structures, snapshots from trajectories, representative input files and any nonstandard simulation parameters have been uploaded to Zenodo, DOI: https://doi.org/10.5281/zenodo.15638241.

## Data Availability

The data that supports the findings of this study are available in the supplementary material of this article and on Zenodo at https://doi.org/10.5281/zenodo.15638241.
