# Supplementary material for: Engineering the substrate scope of the thermostable phenolic acid decarboxylase N31 towards sterically hindered phenolic acids
Source: Protein Sci. 2026 Jul 12;35(8):e70692. doi: 10.1002/pro.70692 (PMC13358373; doi:10.1002/pro.70692)
Supplement: Supplementary file 1 — FIGURE S1. Purification of PAD variants. (a) wild‐type PAD N31. (b) PAD N31 S–. (c) PAD N31 ‐S‐. (d) PAD N31 –A. (e) PAD N31 SS‐. (f) PAD N31 S‐A. (g) PAD N31 SSA. (h) PAD N2. (i) PAD N4. (j) PAD N80. (k) PAD N100. L: PageRuler Prestained Protein Ladder, 10 to 180 kDa (ThermoFisher Scientific) for (a–g), PL00001 Prestained Protein Marker (10–180 kDa), Proteintech, D: debris, CFE: soluble CFE fraction, FT: flowthrough, W: wash, E: elution, WE: wash after elution and RE: Rebuffered elution fraction. Expected size: 21.9 kDa. FIGURE S2. Specific activity of PAD ancestors towards FAc at 30°C with various concentrations—Michaelis–Menten kinetics analysis. The photometric assay was performed in potassium phosphate buffer (50 mM, pH 6) with a final volume of 100 μL containing 12.5 μg enzyme and 0 to 4 mM FAc (FAc stock: 200 mM in DMSO). The reaction was carried out at 30°C and observed for 5 min at 344 nm. Fitting was performed in Origin with the standard Michaelis–Menten fit (see also Material and Methods part for further details). GRG Nonlinear Solving Method for nonlinear optimization was used. FIGURE S3. Specific activity of PAD ancestors towards FAc at 50°C with various concentrations—Michaelis–Menten kinetics analysis. (a) N2, (b) N4, (c) N80, (d) N100, (e) N31. The photometric assay was performed in potassium phosphate buffer (50 mM, pH 6) with a final volume of 100 μL containing 12.5 μg enzyme and 0 to 4 mM FAc (FAc stock: 200 mM in DMSO). The reaction was carried out at 50°C and observed for 5 min at 344 nm. Fitting was performed in Origin with the standard Michaelis Menten fit (see also Materials and Methods part for further details). GRG Nonlinear Solving Method for nonlinear optimization was used. FIGURE S4. Specific activity of PAD ancestors towards SAc at 30°C with various concentrations—Michaelis–Menten kinetics analysis. (a) N2, (b) N4, (c) N80, (d) N100, (e) N31. The photometric assay was performed in potassium phosphate buffer (50 mM, pH 6) with a final vo [file PRO-35-e70692-s001.pdf]

SUPPORTING INFORMATION

Engineering the substrate scope of the thermostable phenolic acid decarboxylase N31 towards sterically hindered phenolic acids

Kristin K. F. Bauer[1,#], Sonja Vaupel[2,#] Jacques Gay[3,#], Eva Vos[3], Aron Wanz[1], Daniel Kracher[1,4], Tobias Schöngassner[1], Lars-Erik Meyer[2], Shina C. L. Kamerlin[3,5,6], Selin Kara[2,6] and Robert Kourist[1,4]**

[#]equal contribution

1. Institute of Molecular Biotechnology, Graz University of Technology, Petersgasse 14, 8010 Graz, Austria

2. Institute of Technical Chemistry, Leibniz University Hannover, Callinstr. 5, 30167 Hannover, Germany

3. School of Chemistry and Biochemistry, Georgia Institute of Technology, Atlanta, Georgia, USA

4. BioTechMed-Graz, Mozartgasse 12, 8010 Graz, Austria

5. School of Chemical and Biomolecular Engineering, Georgia Institute of Technology, Atlanta, Georgia, USA

6. Department of Chemistry, Lund University, Lund, Sweden

7. Biocatalysis and Bioprocessing Group, Department of Biological and Chemical Engineering, Aarhus University, Gustav Wieds Vej 10, 8000 Aarhus, Denmark

E-mail: kourist@tugraz.at; selin.kara@bce.au.dk; selin.kara@iftc.uni-hannover.de

1. EXPERIMENTAL PROCEDURES S3
2. SUPPORTING RESULTS S11
Preparation of the enzyme S11
Michaelis-Menten kinetics analysis for FAc at 30 °C S12
Michaelis-Menten kinetics analysis for FAc at 50 °C S13
Michaelis-Menten kinetics analysis for SAc at 30 °C S14
Michaelis-Menten kinetics analysis for SAc at 50 °C S15
Half-life time analysis S16
Product inhibition analysis of 4-VG with FAc as substrate S17
Product inhibition analysis of 4-VS with SAc as substrate S19
Screening of the combinatorial library S21
Michaelis-Menten kinetics analysis for N31 SSA at 30 °C S26
Product inhibition analysis of PAD N31 of 4-VG with FAc as substrate and S27
4-VS with SAc as substrate S27
Molecular Dynamics (MD) Simulations S28
References S44

1. EXPERIMENTAL PROCEDURES

Materials: Chemicals, cultivation media components, and reagents were sourced from MERCK (Darmstadt, Germany), VWR (Radnor, PA), and Enamine (Kyiv, Ukraine). Ferulic acid was utilized as supplied. The BCA protein quantification kit and molecular biology reagents were obtained from Thermo Scientific (Waltham, MA). Choline chloride and glycerol were procured from Sigma Aldrich. Protein and DNA concentrations were determined using a Nanodrop 2000 UV-Vis spectrophotometer (Thermo Scientific).

Mutagenesis & cloning of the PAD N31 combinatorial library: Firstly, fragments were created via PCR using mutagenic or Gibson assembly. The reaction mixture for each fragment consisted of 5 µL 10 µM fw primers, 5 µL 10 µM rv primers, 20 µL 5× HF buffer (ThermoFisher Scientific), 2 µL 10 mM dNTPs, 20 ng pET28a_PAD_N31, 1 µL Phusion polymerase (2 U/µL) and nuclease-free water to 100 µL. The PCR was conducted by one cycle at 98 °C for 30 s, followed by cycling 30 times a program consisting of 10 s at 98 °C, a temperature gradient going from 58 °C to 72 °C for 20 s, 15 s at 72 °C; a final extension for 5 min was performed at 72 °C. The fragments were checked on a 3 % (w/v) agarose gel (containing 1x Atlas ClearSight DNA stain, LabConsulting), which ran at 85 V, 400 mA for 40 min using TAE buffer (40 mM Tris pH 7.6, 20 mM acetic acid, 1 mM EDTA). The gel was assessed using UV light. The correctly sized fragments were gel-purified using the Promega SV gel and PCR clean-up system according to the manufacturer's instructions.

After successful gel purification, equimolar quantities (187 fmol) of adjacent fragments were mixed with 4 µL 5x HF buffer (ThermoFisher Scientific), 0.4 µL 10 mM dNTPs, 0.2 µL Phusion polymerase (2 U/µL), and nuclease-free water to 20 µL. The mixture was incubated for 15 cycles at 98 °C for 10 s, 61.2 °C/63.2 °C/64.7 °C (fragment 1+2/fragment 2+3/fragment 3+4) and 10 s at 72 °C. All three reactions were then mixed and incubated for 20 cycles of 98 °C for 10 s and 30 s at 72 °C. Afterwards, 2.4 µL of each fw and rv Gibson primer (10 mM) was added, and the mixture was heated to 98 °C for 30 s, followed by a cycling 30 times through 10 s at 98 °C, 30 s at 60.2 °C and 20 s

at 72 °C. An additional 20 times cycle of 30 s at 98 °C and 20 s at 72 °C was performed afterwards.

The successful generation of the full insert was checked again on a 3 % (w/v) agarose gel (containing 1x Atlas ClearSight DNA stain, LabConsulting) and gel-purified as mentioned above. To verify that a combinatorial library was created, the insert was subcloned into the pJET vector (ThermoFisher Scientific) according to the manufacturer's instructions, and 8 clones were picked, plasmid purified using the Promega Plus SV Minipreps DNA purification system and sent for Sanger sequencing (Microsynth, Austria). For the backbone linearization, a mixture consisting of 20 µL 5× HF buffer (ThermoFisher Scientific), 2 µL 10 mM dNTPs, 5 µL of each 10 µM primer, 20 ng of pET28a_PAD_N31, 1 µL Phusion (2 U/µL) and nuclease-free water to 100 µL. The temperature profile was set to one cycle at 98 °C for 30 s, followed by 30 cycles consisting of 10 s at 98 °C, 15 s at 63.6 °C and 2.5 min at 72 °C. A final extension was carried out at 72 °C for 5 min. The fragment was analyzed on a 1 % agarose gel (containing 1x Atlas ClearSight DNA stain, LabConsulting), which ran at 120 V, 400 mA for 35 min. To the PCR reaction, 2 µL of DpnI (NEB, 20 U/µL) was added and incubated for 2 ½ h at 37 °C, followed by an inactivation for 20 min at 80 °C.

The Gibson assembly was performed according to the published protocol. The Gibson assembly was desalted for 1 h using a Millipore-membrane filter (Merck, 0.22 µm). Two aliquots of electro-competent *E. coli* BL21(DE3) were transformed with 7 µL each of the desalted Gibson assembly. The electroporation was performed using a BioRad electroporator (EC2 setting). The cells were regenerated with 1 mL LB-SOC media (20 g/L tryptone, 5 g/L yeast extract, 0.584 g/L NaCl, 2.5 mL/L 1 M KCl, 10 mL/L 1 M $MgSO_4$, 10 mL/L 1 M $MgCl_2$, 20 mL/L 1 M glucose) for 2 h at 37 °C and 600 rpm on a thermoblock. Afterwards, the cells were plated on large squared LB agar plates (10 g/L tryptone, 10 g/L NaCl, 5 g/L yeast extract, 15 g/L agar) containing 40 µg/µL kanamycin (32cmx32cm).

Spectrophotometric screening of PAD activity in a 96-well format: Firstly, a 400 µL lysogeny broth (LB) (10 g/L tryptone, 10 g/L NaCl, 5 g/L yeast extract) ONC, containing 40 µg/µL kanamycin was inoculated with a colony (either from the library or with a *E. coli* BL21(DE3) pET28a control) in a 96-deep well plate (DWP) at 37 °C

and 320 rpm on an Infors HT shaker with a 25 mm throw on a slanted fixture for 96-DWPs. Two sterile controls were added at A1 & A2, the two empty vector controls (*E. coli* BL21 (DE3) pET28a) were always cultivated in C1 & D1 and *E. coli* BL21 (DE3) pET28a_PAD_N31(wt) was cultivated in E1, F1, G1 & H1. The following day, a main culture was inoculated using 6 µL of the ONC and 594 µL of terrific broth auto induction medium (TB-AIM; 12 g/L tryptone, 24 g/L yeast extract, 4 mL/L glycerol, 2.31 g/L KH_2PO_4, 12.54 g/L K_2HPO_4, 0.5 g glucose, 2 g/L α-lactose, 5 g/L glycerol) containing 40 µg/µL kanamycin in a sterile 96-DWP. The main culture was first cultivated for 4 h at 37 °C, followed by 18 h incubation at 28 °C. For the lysis, the cultures were centrifuged in an Eppendorf 5810R centrifuge (4 °C, 3,220 × g, 20 min), and the supernatant was discarded. 300 µL of BugBuster (1x, Millipore) was used to resuspend each cell pellet, and the mixture was incubated at room temperature for 20 min on a Heidolph Titramax 1000 at 900 rpm.

The suspension was centrifuged again for 20 min using an Eppendorf 5810R centrifuge (4 °C, 3,220 × g). The assay was performed using a BMG Labtech Fluostar Omega photometer (rapid scanning mode with three measurements per scan, 29 s/plate with the wavelengths set at 335 nm for SAc/CAc and 333 nm for FAc, for a total of 20 min, 30 °C) in a 96-microtiter plate (Greiner) to monitor the substrate decrease. 50 µL of substrate stock (in 50 mM KPi, pH 6, final concentration in the assay: 1.5 mM) was dispensed using the Gilson Platemaster, followed by 50 µL of the supernatant from the lysed cells. The dilutions for the supernatant were chosen according to the linearity of the decreasing substrate using lysed *E. coli* BL21(DE3) cells harbouring pET28a_PAD_N31 wt as a reference, which corresponded to 10 µg/mL CFE for FAc & CAc and 5,500 µg/mL for Sac.

Plasmid isolation: Cells were grown overnight at 37 °C in 5 mL of LB medium (10 g/L tryptone, 10 g/L NaCl, 5 g/L yeast extract), supplemented with the appropriate antibiotic for the plasmid. The cells were pelleted, and the plasmid isolation was performed either with the GeneJET plasmid miniprep Kit (ThermoFisher Scientific) or Wizard Plus SV Minipreps DNA purification systems (Promega) according to the manufacturer's instructions.

DNA concentration determination: The DNA concentration was mostly determined using a NanoDrop 2000c spectrophotometer (Peqlab), unless stated otherwise. The concentration was determined at 260 nm. To evaluate impurities, additional measurements were taken at 280 and 230 nm.

Protein analysis via SDS-PAGE: The protein analysis was done via sodium dodecyl sulphate polyacrylamide gel electrophoresis (SDS-PAGE). Unless stated otherwise, 1 mL of a culture was withdrawn, and the OD_{600} was measured. The aliquot was pelleted for 2 min at maximum speed using a microcentrifuge (centrifuge 5425, Eppendorf) and stored at -20 °C until usage. The pellet was resuspended with BugBuster (Merck) according to the calculation below.

$$\mu L\ (BugBuster) = \frac{OD_{600} * volume\ pelleted\ (\mu L)}{30}$$

After an incubation period of 20 min at 22 °C, with 300 rpm shaking on a thermoblock, the mixture was centrifuged again for 20 min, 4 °C at max speed using a microcentrifuge. The supernatant was removed into a new micro reaction tube (= soluble phase) and the pellet was resuspended in 50 mM KPi (pH 6) (for PADs) with the same amount which was used for the BugBuster (= insoluble phase). 13 µL of the insoluble/soluble phase was mixed with 5 µL of 4x NuPAGE LDS sample buffer (Invitrogen) and 2 µL of NuPAGE reducing agent (Invitrogen). The mixed sample was heated for 10 min at 70 °C. The samples were loaded on a 4-12 % NuPAGE Bis-Tris gel (Invitrogen) and run with a NuPAGE MES SDS running buffer (Invitrogen). The gel was typically run at 200 V, 60 mA for 45-60 min.
Once the electrophoresis was completed, the gel was stained overnight using a staining solution consisting of 110 mg Coomassie Blue G-250, 50 g ethanol, 80 g phosphoric acid, 850 mL ddH_2O and 10 g β-cyclodextrin.

Protein concentration determination via bicinchoninic acid (BCA) assay: The protein concentration was determined by the Pierce BCA Protein Assay Kit (ThermoFisher Scientific) according to the manufacturer's instructions.

Simulation Setup and Structure Preparation for Molecular Dynamics Simulations: Molecular dynamics (MD) simulations were performed to study the conformational dynamics of the wild-type (N31), triple-mutant (N31 SSA), and single mutants (I29S, L80S, I93A) phenolic acid decarboxylases unliganded and in complex with two substrates, ferulic acid (FAc) and synaptic acid (SAc). Product substrates were performed on N31 and SSA with FAc product (4-VG) and SAc product (4-VS). For the wild-type system, the available crystallographic structure was used as a starting point for the simulation (PDB ID: 8B30, Myrtollari et al. 2024). The variant structures were generated by inserting the corresponding substitutions (Ile29Ser, Leu80Ser and Ile93Ala) into the wild-type crystal structure, using the PyMOL (DeLano 2021) "Mutagenesis" function. Rotamers were selected from the Backbone-Dependent Rotamer library (Dunbrack, JR. und Cohen 1997) such as to eliminate structural clashes.

The liganded systems were prepared by incorporating the protonated form of FAc or SAc into the active site of the respective decarboxylases. To achieve this, we first performed molecular docking using the DiffDock Web server (Corso et al. 2022), arXiv:2210.01776 [q-bio.BM]). The best-ranked (with a confidence value of -1.12) obtained by the docking prediction was then manually modified using PyMOL (DeLano, 2021) to orient the substrate into a good reactive position according to the QM optimized structure reported by Sheng *et al.* (Sheng et al. 2015). 4-VG and 4-VS were manually docked into the corresponding PAD variants through alignment with their reactant counterparts. The partial charges for the FAc, SAc, 4-VG, and 4-VS substrates were computed based on Restrained Electrostatic Potential (RESP) (Woods and Chappelle R. 2000) fitting using Antechamber (Wang et al. 2006). The electrostatic potential of the two substrates was calculated at the HF/6-31G(d) level of theory, after optimizing the geometries of each substrate at the B3LYP/6-31G(d) level of theory, using Gaussian 16 Rev. B.01 (Frisch et al. 2016). All other parameters to describe the substrates were obtained using the General AMBER Force Field 2 (GAFF2) (Wang et al. 2004). All non-standard parameters to describe FAc, SAc, 4-VG, and 4-VS substrates are provided in Table S10 – S13, respectively and in the Zenodo data package available at https://doi.org/10.5281/zenodo.15638241.

The protonation states of ionizable residues in all systems were determined using PROPKA 3.0 (Olsson et al. 2011). Based on these results, and on visual inspection of the local environments of amino acids with ionizable side-chains, all ionizable residues were kept in their standard protonation state at physiologic pH, with the exception of Glu72, which was modelled in its neutral form as it is involved in the catalytic reaction (Sheng et al. 2015; Rodríguez et al. 2010). However, to mimic the protein state after catalysis, all product simulations were done with Glu72 in its deprotonated form. Finally, based on visual analysis, all histidine residues were modelled as being protonated at N_{ε} (His-ε).

The resulting nineteen systems (unliganded N31, FAc bound-N31, SAc bound-N31, 4-VG bound-N31, 4-VS bound-N31, unliganded N31 SSA, FAc bound-N31 SSA, SAc bound-N31 SSA, 4-VG bound-N31 SSA, 4-VS bound-N31 SSA, unliganded N31 I29S, FAc bound-N31 I29S, SAc bound-N31 I29s, unliganded N31 L80S, FAc bound-N31 L80S, SAc bound-N31 L80S, unliganded N31 I93A, FAc bound-N31 I93A, SAc bound-N31 I93A) were solvated in a truncated octahedral water box of OPC water molecules, extending 11.0Å from the protein in all directions (Izadi et al. 2014). To ensure the charge neutrality of the systems, 20 Na^+ counterions were added to all simulations of systems containing FAc or SAc, 19 Na^+ to all simulations of systems containing 4-VG, 4-VS, or unliganded. Additionally, the hydrogen atoms were scaled using hydrogen mass repartitioning (Hopkins et al. 2015).

Molecular Dynamics Equilibration and Production Procedure: All MD simulations in this work were performed using the HIP-accelerated version of Amber24 using the ff19SB force field and the OPC water model (Case et al. 2025; Case et al. 2023; Tian et al. 2020; Izadi et al. 2014).

All systems were equilibrated for MD simulations using the same protocol. First, energy minimization was performed using the steepest descent algorithm for 100 steps, and then 900 steps of conjugate gradient minimization were performed, with a 100 kcal mol^{-1} $Å^{-2}$ restraint applied to all solute (protein and substrate) atoms. The minimized system was then heated for 1 ns from 50 to 300K in an NVT ensemble, using a simulated annealing protocol, in which the system reached 300K in the first 100 ps of heating. Next, we continued with a 1 ns long NPT simulation and Langevin temperature

control, with a collision frequency of 1 ps^{-1} and keeping the 100 kcal mol^{-1} $Å^{-2}$ restraint to the solute. From this point, the applied restraints were reduced from 100 to 10 kcal mol^{-1} $Å^{-2}$ in subsequent equilibration steps. A second energy minimization was performed using the prior protocol, with positional restraints applied to just solute-heavy atoms. During the subsequent equilibration steps, the restraints were progressively reduced from 10 to 1 to 0.1 kcal mol^{-1} $Å^{-2}$, until the heavy atom restraints were removed in the final step. The described equilibration protocol was performed for five different replicas per system. Finally, production MD runs were performed using an 8 Å direct space non-bonded cutoff, Langevin temperature control (collision frequency of 1 ps^{-1}), and a Berendsen barostat (pressure relaxation time of 1 ps). The total production trajectories for all systems were 1 μs in length each, and each system was simulated in 5 different replicas, leading to a cumulative 5 □s of simulation time per system and 95 □s of simulation time across all systems. The equilibration of the trajectories is shown in Figure S22 for the reactant liganded system and S23 for unliganded and product liganded systems. For all the MD simulations, we have set up a 4 fs time step and facilitated the SHAKE algorithm to constrain all bonds containing hydrogen atoms (Ryckaert et al. 1977).

Simulation Analysis: All analysis of MD simulations were performed using the CPPTRAJ module of AmberTools23, unless otherwise indicated (Roe and Cheatham 2013; Case et al. 2023). Simulation snapshots were extracted for analysis every 200 ps of each trajectory (5 trajectories per system), and the analysis presented in this work is the average values and standard deviations over 5 x 1 μs trajectories per system.

The distance of the substrates and relevant catalytic residues (Tyr19, Tyr21, Arg49 and Glu72) was computed using CPPTRAJ (Roe and Cheatham 2013). For substrate-Arg49 and substrate-Glu72 distances, we considered the distance between the carbon atom of the substrate carboxylic group and the CZ and CD atoms of Arg49 and Glu72, respectively. For substrate-tyrosine distances, the value was calculated by attending to the distance between the oxygen atom of the substrate phenolic hydroxyl and the oxygen atom of the phenolic hydroxyl of residues Tyr19 and Tyr21.

Clustering analysis was performed using hierarchical agglomerative clustering as implemented into CPPTRAJ. Root mean square deviation (RMSD) clustering of the substrate used five distinct clusters. Additionally, Glu72 clustering was performed on the χ_1 and χ_2 dihedrals with 3 distinct clusters supported *via* manual binning.

Active site volumes for the different variants were calculated using MDPocket (Schmidtke *et al.* 2011) with snapshots taken every 200 ps of the simulations. Analyzed pockets were selected by filtering out isosurface values ≥ 3.5, and further trimming out density grids not falling within the pocket of interest.

Hydrogen bond analysis between residues and ligands was performed using the “hbond” CPPTRAJ analysis command between heavy atom H-bond donors and acceptors within the ligand and nearby residues, with a cutoff value of 3.5 Å, with snapshots taken every 1 ns of the simulations (Roe and Cheatham 2013).

Finally, the PyMOL software was employed for the visualization of the systems (DeLano, 2021), and VMD 2.0 software was used to analyze potential water networks (Humphrey, W., Dalke, A. and Schulten, K. 1996).

2. SUPPORTING RESULTS

Preparation of the enzyme

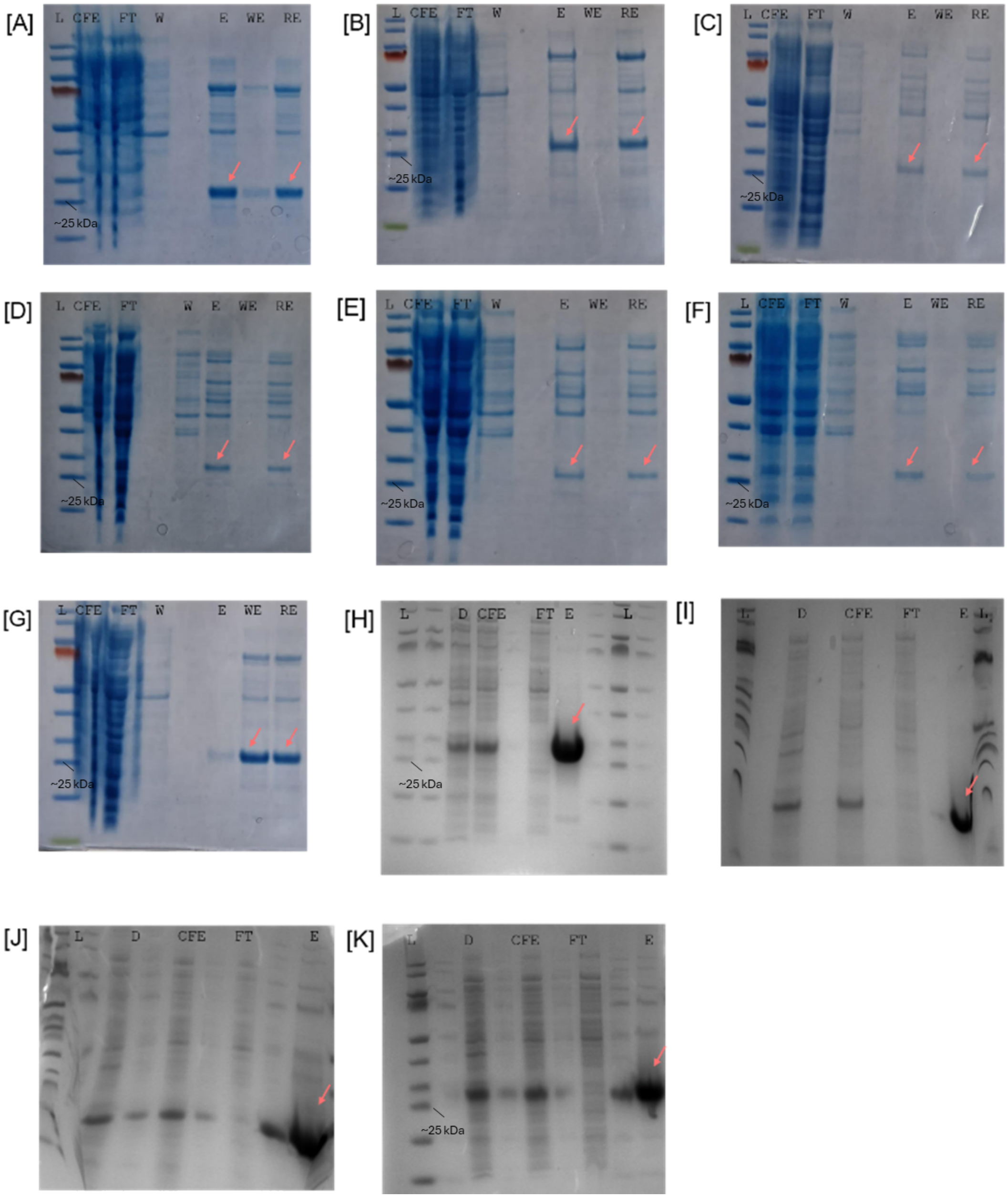

Figure S1: Purification of PAD variants. [A]: wild-type PAD N31. [B]: PAD N31 S--. [C]: PAD N31 -S-. [D]: PAD N31 --A. [E]: PAD N31 SS-. [F]: PAD N31 S-A. [G]: PAD N31 SSA [H] PAD N2. [I] PAD N4. [J] PAD N80. [K] PAD N100. L: PageRuler Prestained Protein Ladder, 10 to 180 kDa (ThermoFisher Scientific) for [A] - [G], PL00001 Prestained Protein Marker (10-180 kDa), Proteintech, D: debris, CFE: soluble CFE fraction, FT: flowthrough, W: wash, E: elution, WE: wash after elution and RE: Rebuffered elution fraction. Expected size: 21.9 kDa.

Michaelis-Menten kinetics analysis for FAc at 30 °C

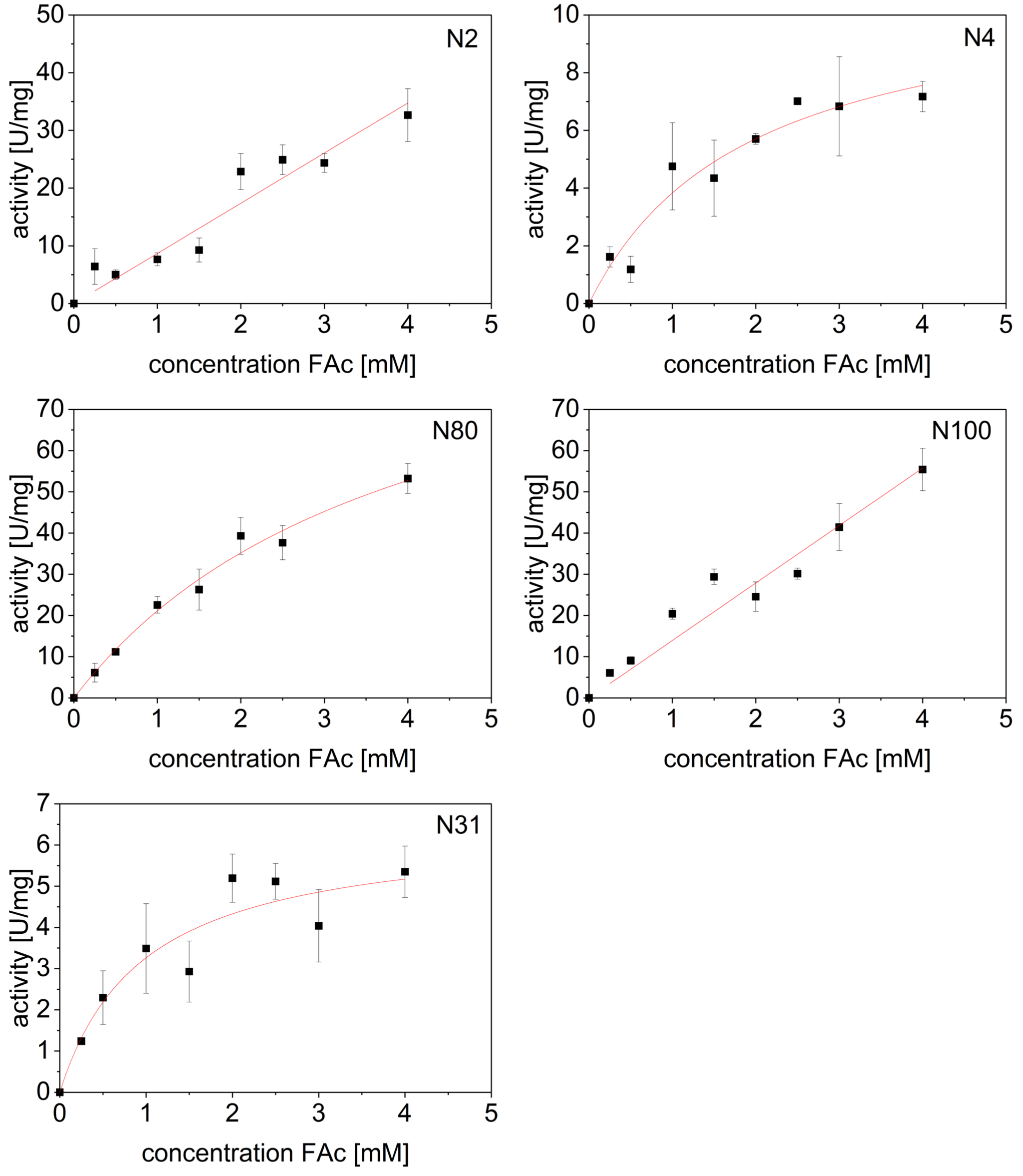

Figure S2: Specific activity of PAD ancestors toward FAc at 30 °C with various concentrations – Michaelis-Menten kinetics analysis. The photometric assay was performed in potassium phosphate buffer (50 mM, pH 6) with a final volume of 100 µL containing 12.5 µg enzyme and 0 to 4 mM FAc (FAc stock: 200 mM in DMSO). The reaction was carried out at 30 °C and observed for 5 min at 344 nm. Fitting was performed in Origin with the standard Michaelis-Menten fit (see also Material and Methods part for further details). GRG Nonlinear Solving Method for nonlinear optimization was used.

Michaelis-Menten kinetics analysis for FAc at 50 °C

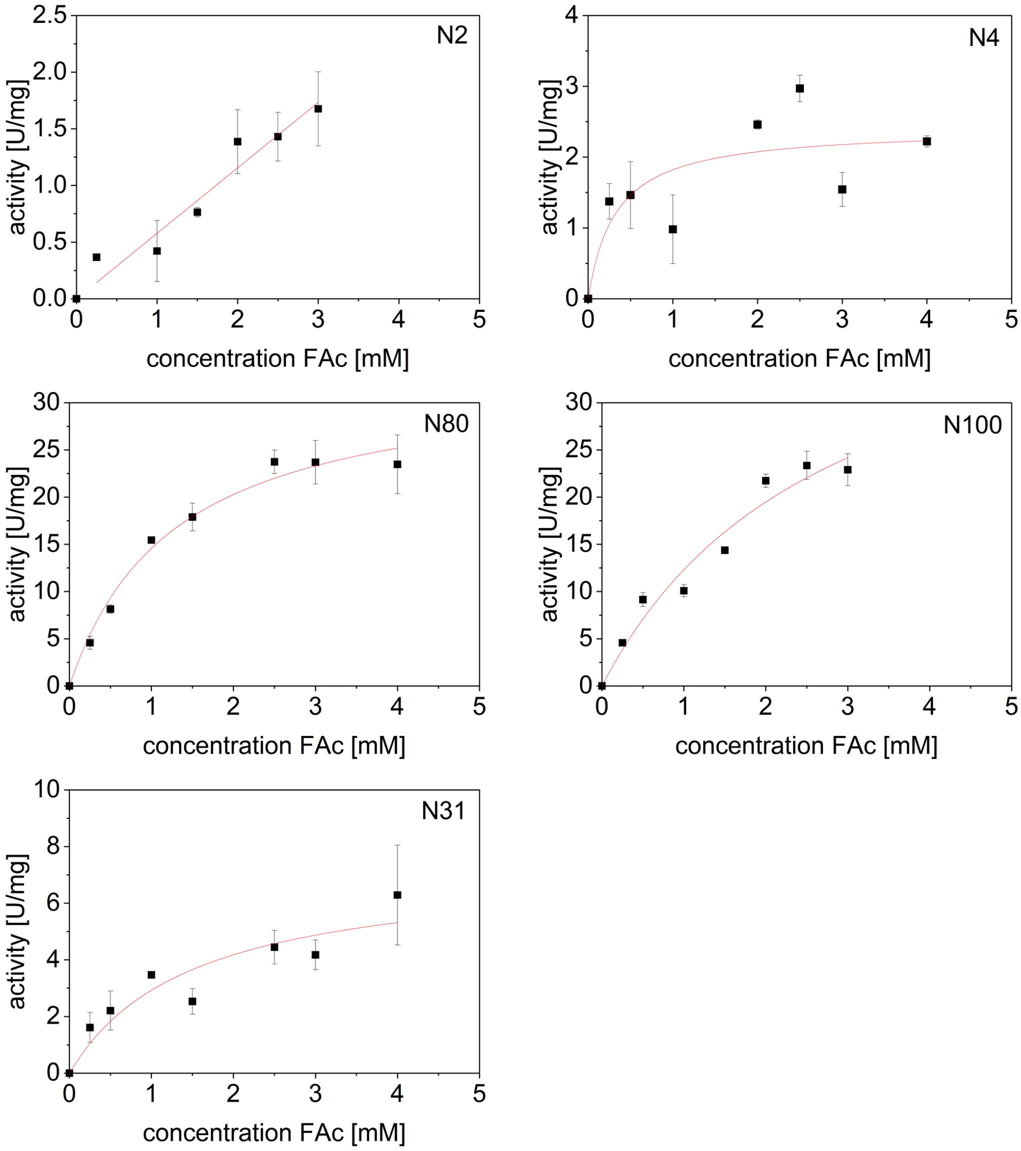

Figure S3: Specific activity of PAD ancestors toward FAc at 50 °C with various concentrations – Michaelis-Menten kinetics analysis. A. N2, B. N4, C. N80, D. N100, E. N31. The photometric assay was performed in potassium phosphate buffer (50 mM, pH 6) with a final volume of 100 µL containing 12.5 µg enzyme and 0 to 4 mM FAc (FAc stock: 200 mM in DMSO). The reaction was carried out at 50 °C and observed for 5 min at 344 nm. Fitting was performed in Origin with the standard Michaelis-Menten fit (see also Materials and Methods part for further details). GRG Nonlinear Solving Method for nonlinear optimization was used.

Michaelis-Menten kinetics analysis for SAc at 30 °C

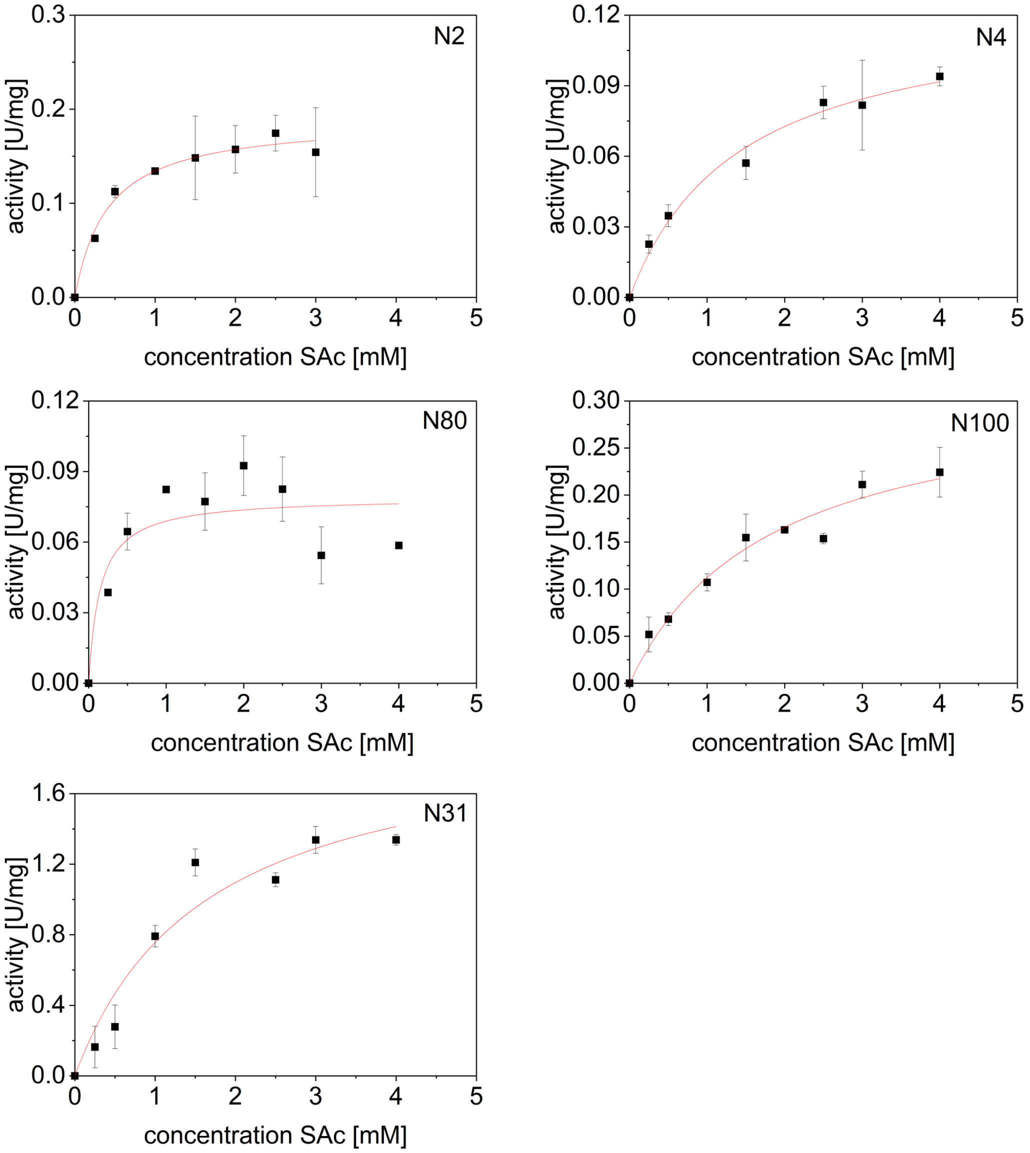

Figure S4: Specific activity of PAD ancestors toward SAc at 30 °C with various concentrations – Michaelis-Menten kinetics analysis. A. N2, B. N4, C. N80, D. N100, E. N31. The photometric assay was performed in potassium phosphate buffer (50 mM, pH 6) with a final volume of 100 µL containing 25 µg enzyme and 0 to 4 mM SAc (SAc stock: 200 mM in DMSO). The reaction was carried out at 30 °C and observed for 5 min at 348 nm. Fitting was performed in Origin with the standard Michaelis-Menten fit (see also Materials and Methods part for further details). GRG Nonlinear Solving Method for nonlinear optimization was used.

Michaelis-Menten kinetics analysis for SAc at 50 °C

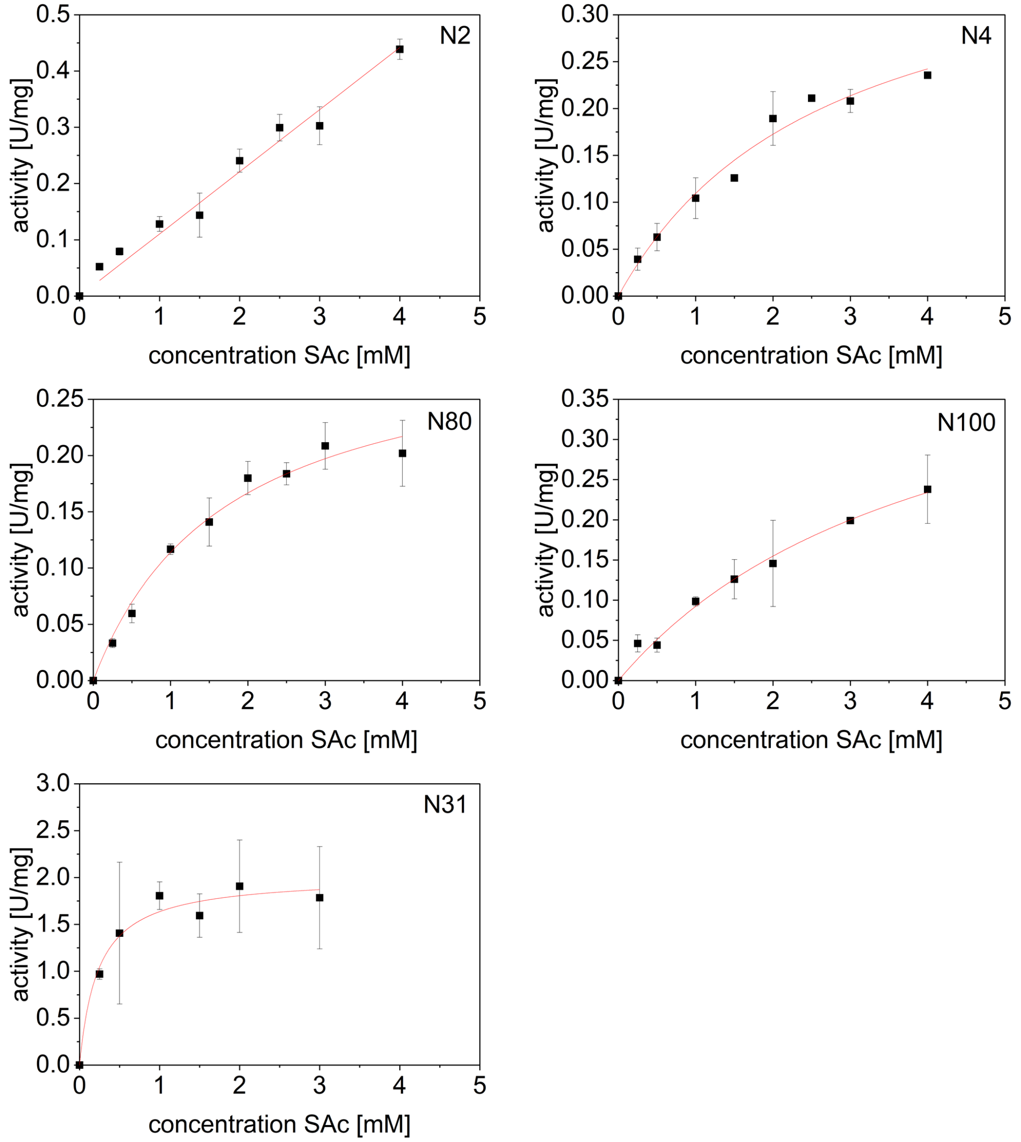

Figure S5: Specific activity of PAD ancestors toward SAc at 50 °C with various concentrations – Michaelis-Menten kinetics analysis. A. N2, B. N4, C. N80, D. N100, E. N31. The photometric assay was performed in potassium phosphate buffer (50 mM, pH 6) with a final volume of 100 µL containing 25 µg enzyme and 0 to 4 mM SAc (SAc stock: 200 mM in DMSO). The reaction was carried out at 50 °C and observed for 5 min at 348 nm. Fitting was performed in Origin with the standard Michaelis-Menten fit (see also Materials and Methods part for further details). GRG Nonlinear Solving Method for nonlinear optimization was used.

Half-life time analysis

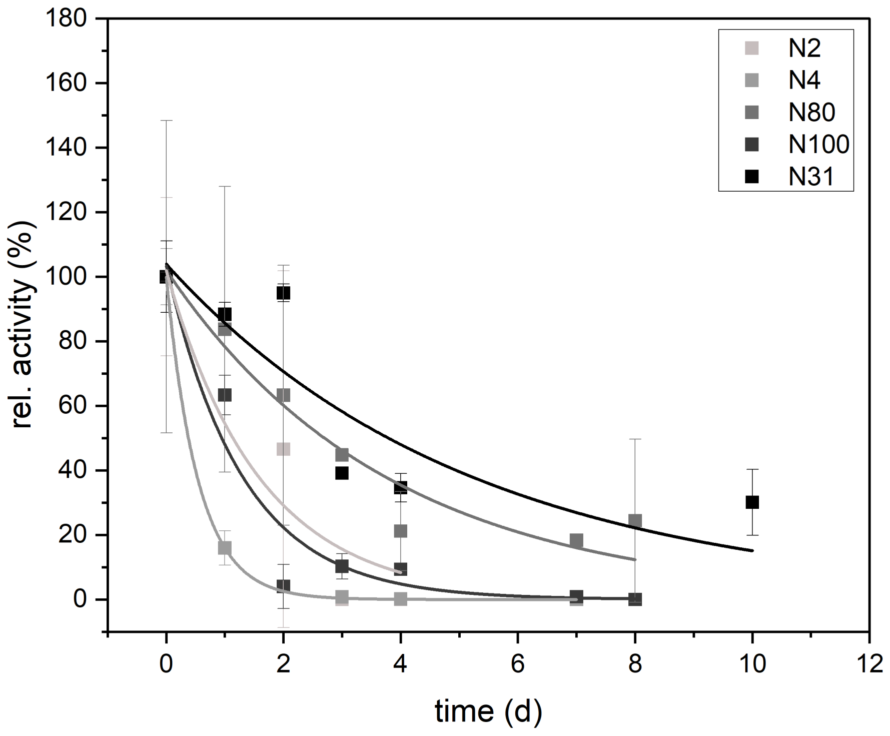

Figure S6: Half-life time measurements for five PAD ancestors and SSA mutant. Aliquots of 50 µL were incubated at 50 °C. Activity measurements were carried out at 30 °C with 4 mM FAc, except SSA was measured with SAc in a photometer. Absorption was followed at 344 nm and 348 nm, respectively.

Table S1: Summary of stability data of the ancestors N2, N4, M80, N100 and N31.

PAD Ancestor	k_{des} (d^{-1})	$t_{1/2}$ (d)
**N2**	0.62 ± 0.22	1.10 ± 0.30
**N4**	1.83 ± 0.02	0.38 ± 0.01
**N80**	0.27 ± 0.04	2.57 ± 0.33
**N100**	0.59 ± 0.06	1.17 ± 0.10
**N31**	0.19 ± 0.07	3.65 ± 0.99

Product inhibition analysis of 4-VG with FAc as substrate

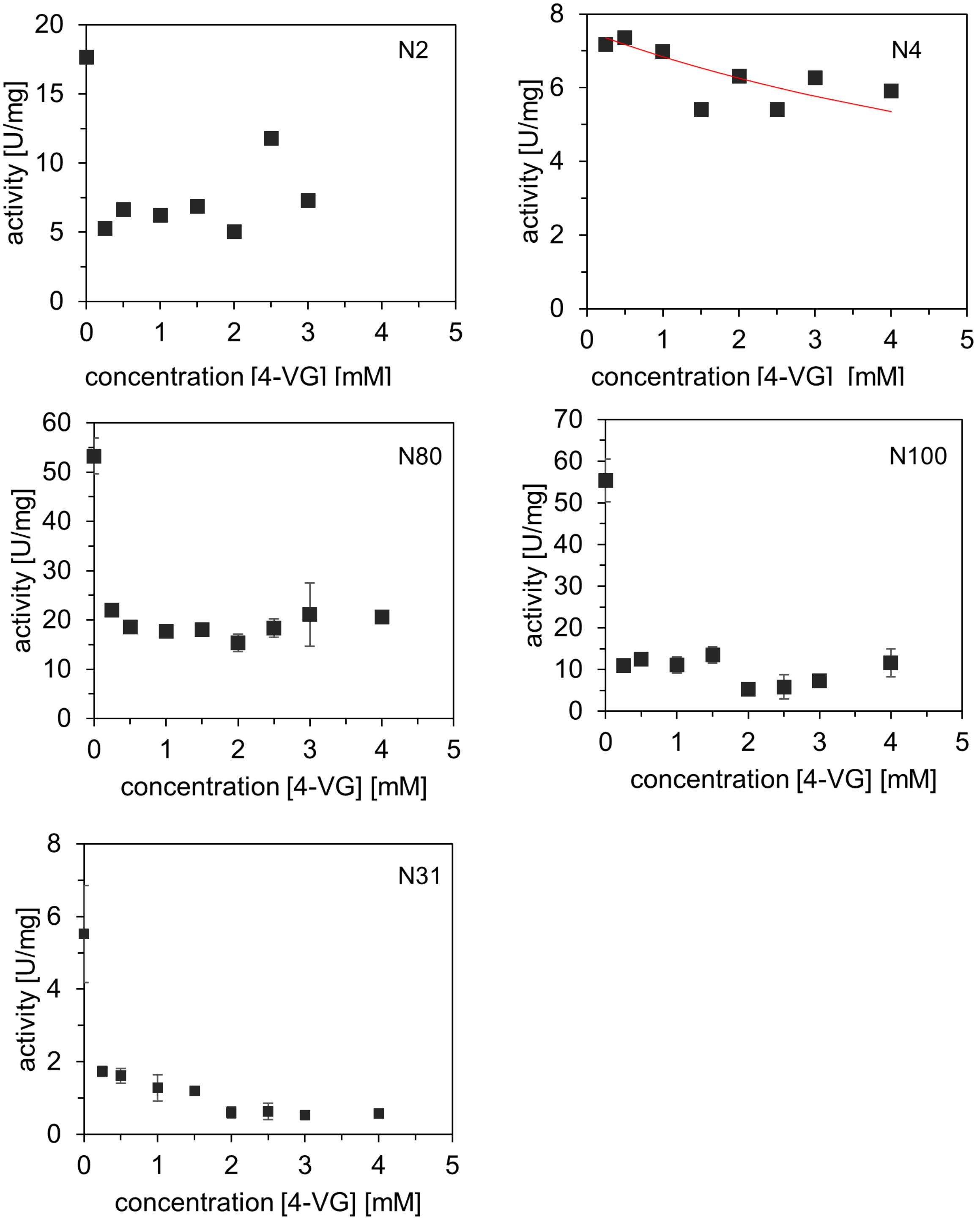

Figure S7: Specific activity of PAD ancestors toward FAc with various concentrations of 4-VG – Product inhibition analysis. The photometric assay was performed in potassium phosphate buffer (50 mM, pH 6) with a final volume of 100 µL containing 12.5 µg enzyme and 0 to 4 mM 4-VG (FAc stock: 200 mM in DMSO). The reaction was carried out at 30 °C and observed for 5 min at 344 nm (see also Materials and Methods part for further details).

Table S2: Summary of kinetic data for product inhibition FAc at 30 °C. GRG Nonlinear Solving Method for nonlinear optimization was used.

PAD Ancestor	K_M (mM)	V_{max} (U/mg)	K_i (mM)	K_i/K_M (-)
**N2**	-	-	-	-
**N4**	1.9 ± 0.8	11 ± 2	3.1	1.63
**N80**	4 ± 1	105 ± 18	-	-
**N100**	-	-	-	-
**N31**	1 ± 0.4	6 ± 1	-	-

Product inhibition analysis of 4-VS with SAc as substrate

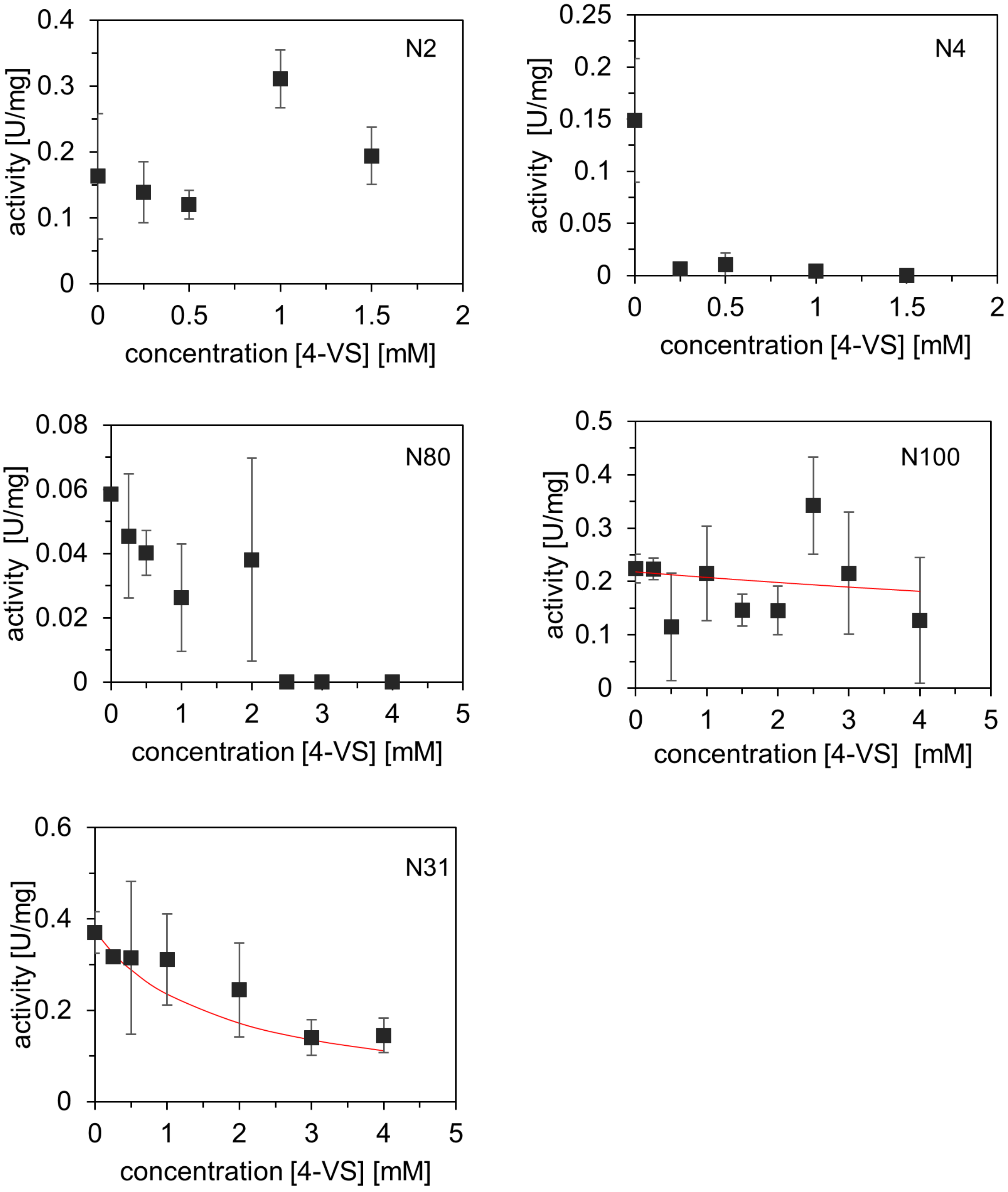

Figure S8: Specific activity of PAD ancestors toward SAc with various concentrations of 4-VS – Product inhibition analysis. The photometric assay was performed in potassium phosphate buffer (50 mM, pH 6) with a final volume of 100 µL containing ca. 25 µg enzyme and 0 to 4 mM 4-VS (SAc stock: 200 mM in DMSO). The reaction was carried out at 30 °C and observed for 5 min at 348 nm.

Table S3: Summary of kinetic data for product inhibition SAc at 30 °C. GRG Nonlinear Solving Method for nonlinear optimization was used.

PAD ancestor	K_M (mM)	V_{max} (U/mg)	K_i (mM)	K_i/K_M (-)
**N2**	0.4 ± 0.08	0.2 ± 0.01	-	-
**N4**	1.4 ± 0.3	0.1 ± 0.01	-	-
**N80**	0.15 ± 0.12	0.08 ± 0.01	-	-
**N100**	1.8 ± 0.5	0.3 ± 0.04	6.3	3.5
**N31**	1.6 ± 0.7	2 ± 0.4	0.47	0.3

Screening of the combinatorial library

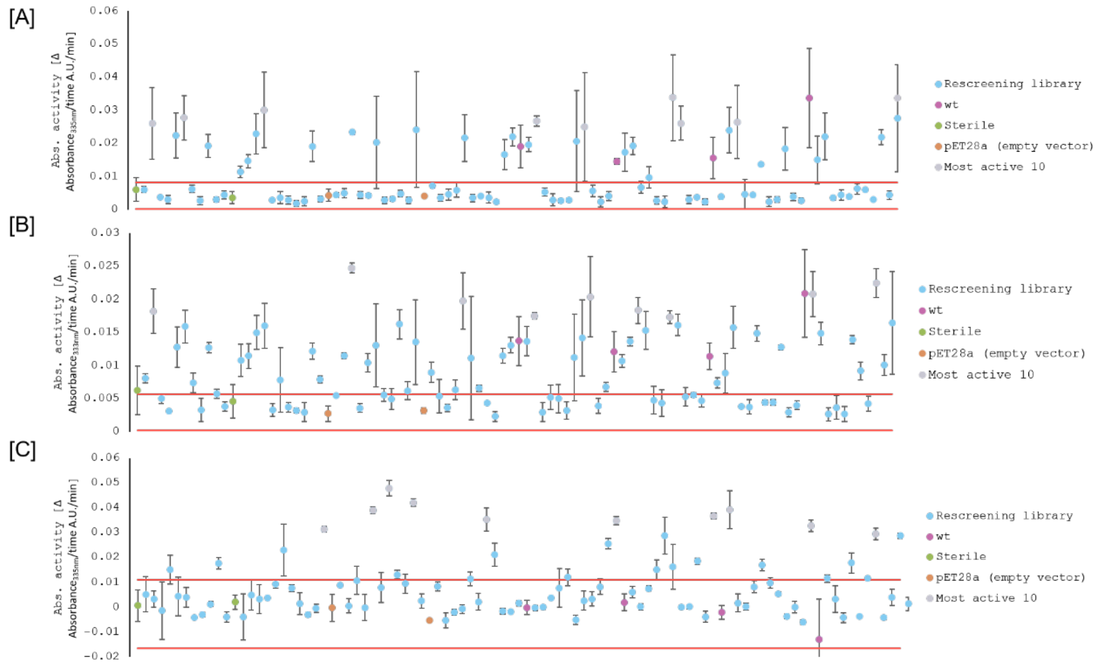

Figure S9: Absolute activities (as Δ A.U. min^{-1}) of the rescreening library towards [A] 1.5 mM CAc, [B] 1.5 mM FAc and [C] 1.5 mM SAc. Reaction conditions: 30 °C, 100 μL total volume, 1.5 mM substrate in potassium phosphate buffer (50 mM, pH 6). Reactions were performed using a cell-free extract (CFE) total protein concentration of 10 μg/mL for CAc and FAc, and 5,500 μg/mL for SAc. N = 3. The red lines indicate ± 3x standard deviation of the mean pET28a (empty vector) control to indicate the noise range for each substrate.

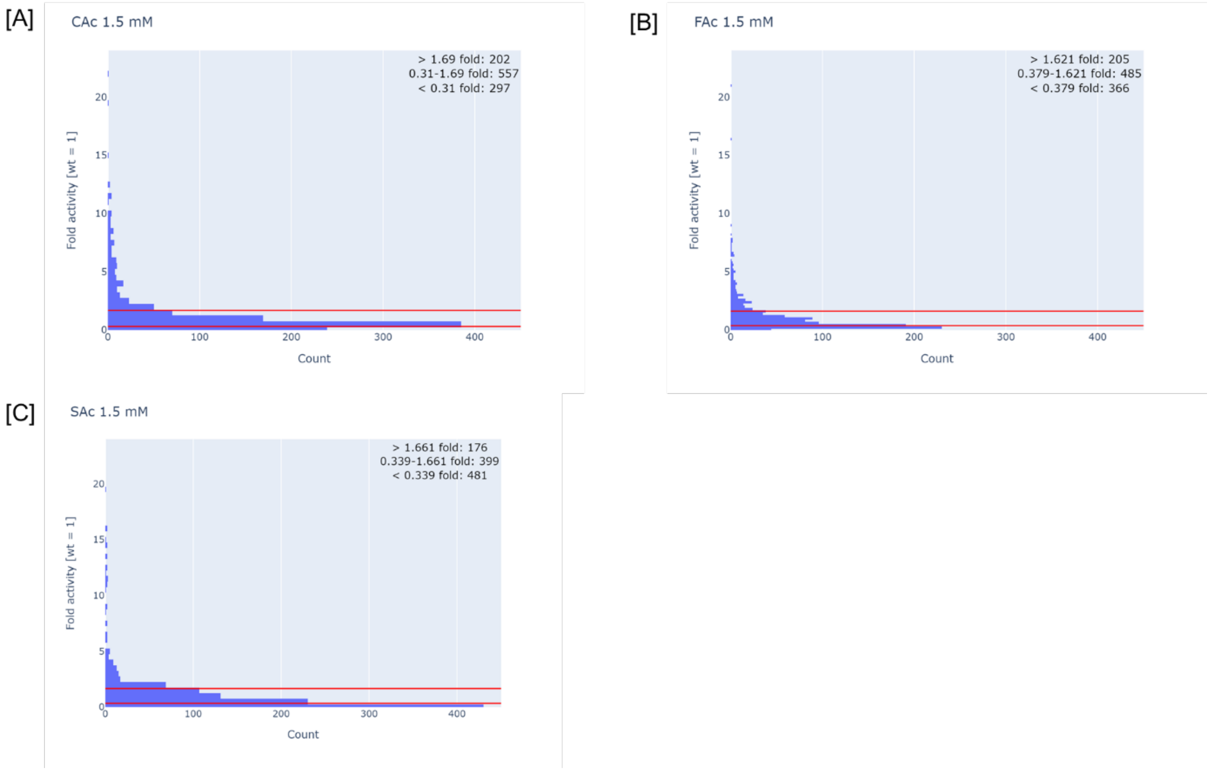

Figure S10: Histogram of the fold change activity (determined as the Δabsorbance over time) of the combinatorial PAD N31 library (1,056 colonies) towards three different substrates. The fold changes were calculated plate-wise to the wt controls present on each plate. The red lines indicate the mean ± standard deviation for the wt controls. The number of colonies within and outside of these ranges is displayed at the right-hand corner of each histogram. [A] 1.5 mM CAc, [B] 1.5 mM FAc and [C] 1.5 mM SAc. Reaction conditions: 30 °C, 100 µL total volume, 1.5 mM substrate in potassium phosphate buffer (50 mM, pH 6). CFE protein concentrations used: 10 µg/mL for CAc & FAc, 5,500 µg/mL for SAc.

Table S4: Activities and sequences (via Sanger sequencing) of the 10 most active colonies from the rescreening process for each substrate tested.

Substrate	Mutation	Abs. activity [ΔAbsorbance/time, A.U./min]
1.5 mM CAc	wt	0.034 ± 0.013
	wt (control)	0.034 ± 0.015
	wt	0.030 ± 0.011
	wt	0.028 ± 0.007
	wt	0.028 ± 0.016
	wt	0.027 ± 0.002
	wt	0.026 ± 0.011
	wt	0.026 ± 0.011
	wt	0.026 ± 0.005
	wt	0.025 ± 0.016
1.5 mM FAc	Ile29Ala	0.025 ± 0.001
	Ile29Ala	0.0225 ± 0.002
	wt (control)	0.0209 ± 0.007
	Ile29Ser, Leu80Val	0.0208 ± 0.003
	Ile29Ala, Leu80Ser	0.0204 ± 0.006
	Ile29Ser	0.0197 ± 0.004
	Ile29Ala, Leu80Ser	0.0183 ± 0.002
	wt	0.0182 ± 0.003
	wt	0.0175 ± 0.000
	wt	0.0173 ± 0.001
1.5 mM SAc	Ile29Ser, Leu80Ser, Ile93Ala	0.048 ± 0.003
	I2le9Ala, Leu80Thr, Ile93Ala	0.042 ± 0.002
	Ile29S, Leu80Val, Ile93Ala	0.039 ± 0.008
	Ile29Ser, Leu80Ser, Ile93Val	0.039 ± 0.001
	Ile29Thr, Leu80Ser, Ile93Ala	0.037 ± 0.001
	Ile29Ala, Leu80Val, Ile93Ala	0.035 ± 0.005
	Ile29Ala, Leu80Thr, Ile93Ala	0.035 ± 0.002
	Ile29Ser, Leu80Val, Ile93Gly	0.033 ± 0.002
	Ile29Ser, Leu80Ser, Ile93Val	0.031 ± 0.001
	Ile29Ser, Leu80?, Ile93?	0.029 ± 0.002

Table S5: Absolute activities (as Δ A.U.min^{-1}) of selected variants from the rescreening of the combinatorial PAD N31 library towards three tested substrates (CAc, FAc and SAc). Reaction conditions: 30 °C, 100 µL total volume, 1.5 mM substrate in potassium phosphate buffer (50 mM, pH 6). Reactions were performed using a cell-free extract (CFE) total protein concentration of 10 µg/mL for CAc and FAc, and 5,500 µg/mL for SAc. N = 3.

	**pET28a**	**PAD N31**	**PAD N31 Ile29Ala**	**PAD N31 Ile29Ser**	**PAD N31 Ile29Ser,Leu80Val**	**PAD N31 Ile29Ala,Leu80Ser**	**PAD N31 Ile29Ser,Leu80Ser,Ile93Ala (SSA)**
**CAc**	0.004 ± 0.001	0.021 ± 0.012	0.022 ± 0.002	0.022 ± 0.007	0.010 ± 0.003	0.007 ± 0.002	0.003 ± 0.001
**FAc**	0.003 ± 0.001	0.015 ± 0.006	0.022 ± 0.002	0.020 ± 0.004	0.015 ± 0.003	0.018 ± 0.002	0.006 ± 0.001
**SAc**	-0.003 ± 0.005	-0.003 ± 0.010	0.004 ± 0.003	0.011 ± 0.003	0.015 ± 0.004	0.008 ± 0.001	0.048 ± 0.003

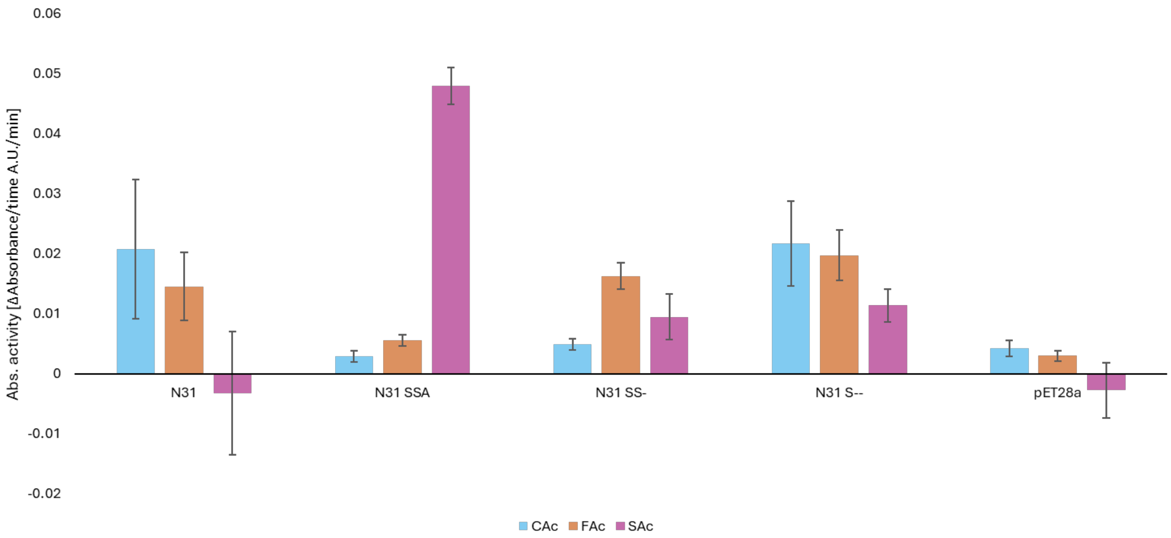

Figure S11: Comparison of selected PAD N31 variants from the rescreening on their absolute activity towards different substrates. N= 12 for PAD N31, N = 3 for mutants and N = 6 for pET28a. Reaction conditions: 30 °C, 100 µL total volume, 1.5 mM substrate in potassium phosphate buffer (50 mM, pH 6). CFE protein concentrations: 10 µg/mL for CAc & FAc, 5,500 µg/mL for SAc. Abbreviations: N31 (N31 wild type), N31 SSA (N31 Ile29Ser,Leu80Ser,Ile93Ala); N31 SS- (N31 Ile29Ser,Leu80Ser); N31 S-- (N31 Ile29Ser).

Michaelis-Menten kinetics analysis for N31 SSA at 30 °C

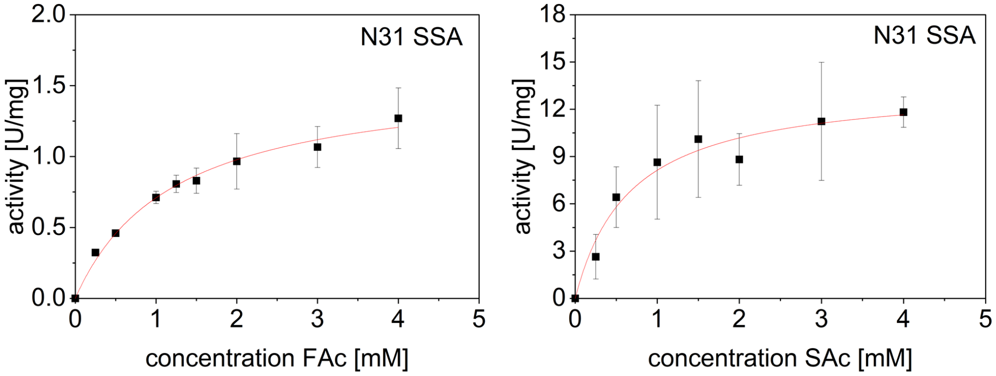

Figure S12: Specific activity of PAD ancestors toward FAc and SAc at 30 °C with various concentrations. The reactions were carried out for [A] FAc at 30 °C, [B] SAc at 30 °C. The photometric assay was performed in potassium phosphate buffer (50 mM, pH 6) with a final volume of 100 µL containing ca.12.5 and 25 µg enzyme and 0 to 4 mM SAc (SAc stock: 200 mM in DMSO). The reaction was carried out at 30 °C and observed for 5 min at 348 nm. Fitting was performed in Origin with the standard Michaelis-Menten fit (see also Materials and Methods part for further details).

Product inhibition analysis of PAD N31 of 4-VG with FAc as substrate and 4-VS with SAc as substrate

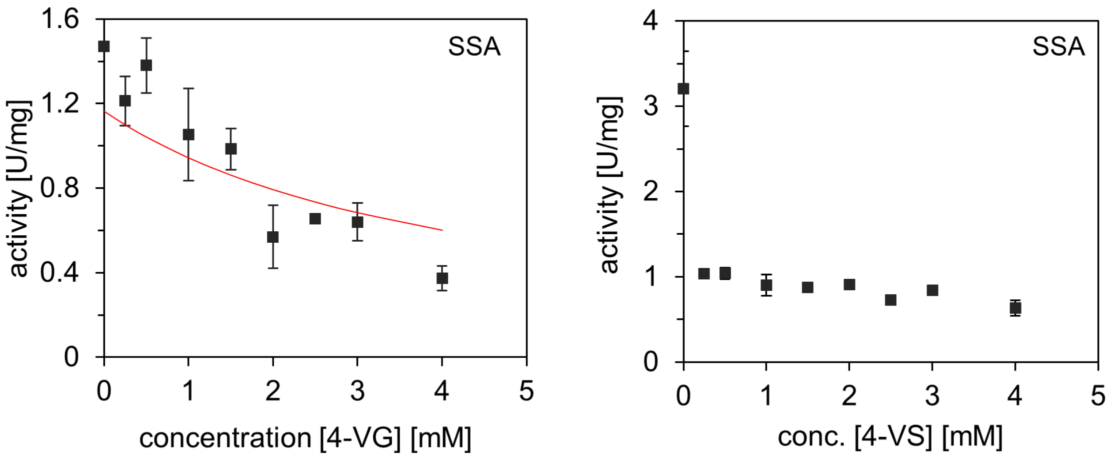

Figure S13: Specific activity of N31 SSA toward FAc and SAc with various concentrations of the respective product. The photometric assay was performed in potassium phosphate buffer (pH 6, 50 mM) with a final volume of 100 µL containing ca. 25 µg enzyme and 0 to 4 mM 4-VG/4-VS (FAc: 200 mM in DMSO; SAc stock: 200 mM in DMSO). The reaction was carried out at 30 °C and observed for 5 min at 344 nm and 348 nm, respectively.

Table S6: Summary of kinetic and stability data for N31 SSA at 30 °C obtained with competitive inhibition curve fit in Origin.

**Michaelis-Menten**	K_M **(mM)**	V_{max} **(U/mg)**	k_{cat}/K_M **(s^{-1}□mM^{-1})**	
**FAc 30 °C**	1.2 ± 0.2	1.6 ± 0.08	0.5 ± 0.2	
**SAc 30 °C**	0.7 ± 0.2	14 ± 1	6.8 ± 1.3	
**Product inhibition**	K_m **(mM)**	V_{max} **(U/mg)**	K_i **(mM)**	K_i/K_m **(-)**
**4-[VG]**	0.29 ± 0.09	1.93 ± 0.09	0.09	0.31
**4-[VS]**	0.83 ± 0.3	6.5 ± 0.78	-	-
**Half-life time**	k_{des} **(d^{-1})**	$t_{1/2}$ **(d)**		
**N31 SSA**	0.62 ± 0.13	1.12 (precipitated on day 3)		

Molecular Dynamics (MD) Simulations

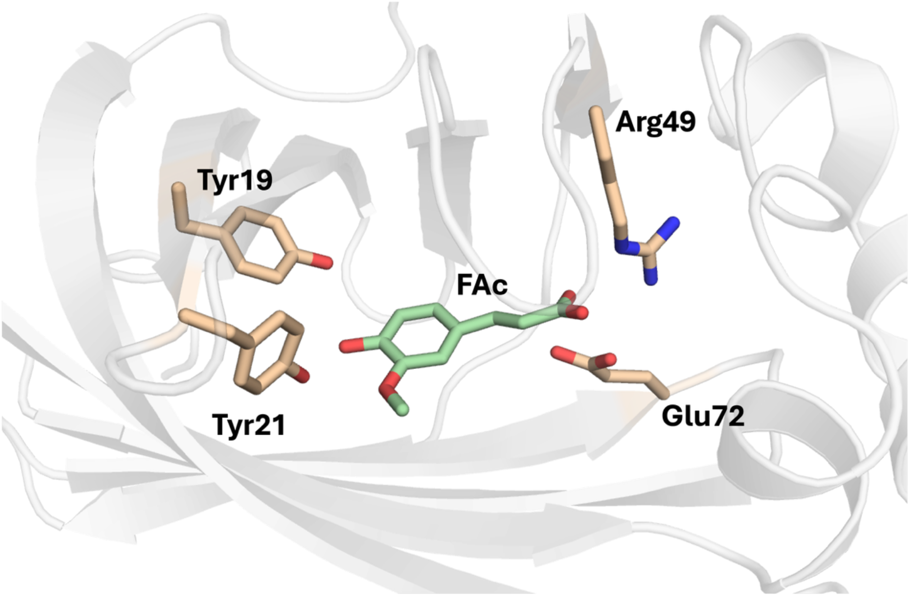

Figure S14: Most likely binding orientation of the substrate FAc in the phenolic acid decarboxylase enzyme (Sheng et al. 2015; Payer et al. 2017). The phenolic hydroxy group is assumed to be deprotonated upon binding to the PAD active site, and stabilized by an oxyanion hole formed by the side chains of Tyr19 and Tyr21, with Glu72 acting as a general acid-base catalyst.

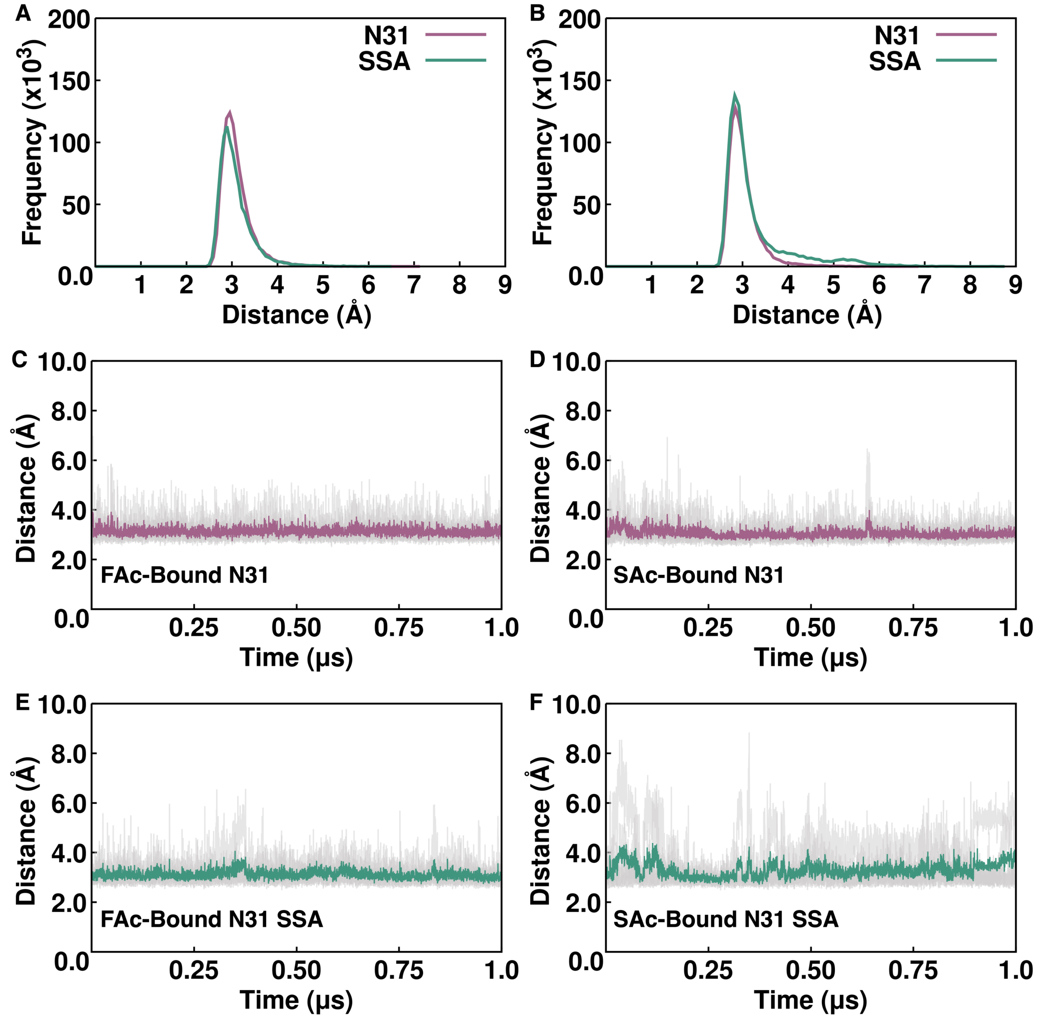

Figure S15: Histograms and time evolution of the distance between Tyr21 and the phenolic oxygen (FAc-Tyr19/SAc-Tyr19, Å) during 5 x 500 ns MD simulations of FAc- and SAc-bound N31 and N31 SSA PAD. Shown here are a comparison of histograms of (A) FAc-bound N31 and N31 SSA, (B) SAc bound N31 and N31 SSA, (C, E) time evolution of the FAc-Tyr19 distance in (C) N31 and (E) N31 SSA, and (D, F) time evolution of the SAc-Tyr19 distance in (D) N31 and (F) N31 SSA PAD, respectively. In panels (C-F), the shaded region shows the distances sampled by the individual trajectories, and the solid region shows the running average over all replicas. The corresponding data for the phenolic oxygen – Tyr21 distance is shown in Figure 4, and the averages and standard deviations for all catalytic distances to both substrates are shown in Table S7.

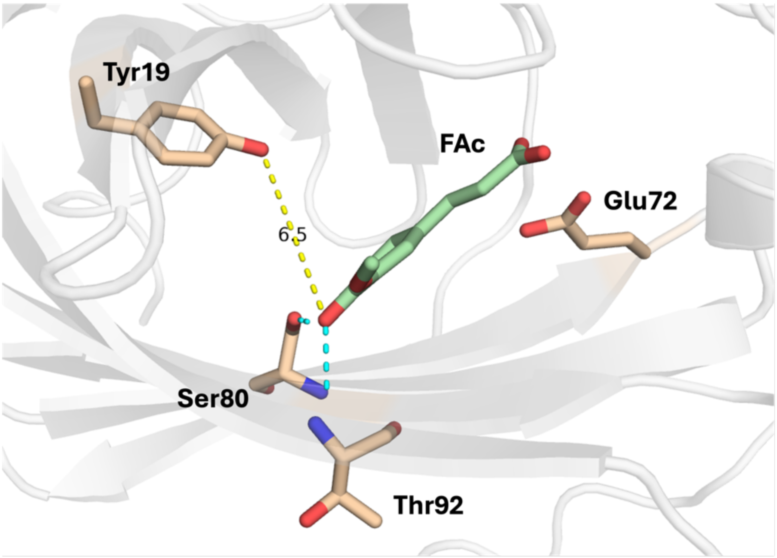

Figure S16: Illustration of the interactions between the ligand and Ser80 and Thr92 in FAc-bound SSA system. The interaction allows the stabilization of a non-reactive conformation of the substrate in the active site, which we observe for 55% of simulation time, based on conformational clustering analysis performed as described in the Supplementary Methods. The cyan dotted lines represent the polar interactions. The yellow dotted lines represent the distances in Å, taken from a representative snapshot collected from ligand clustering analysis.

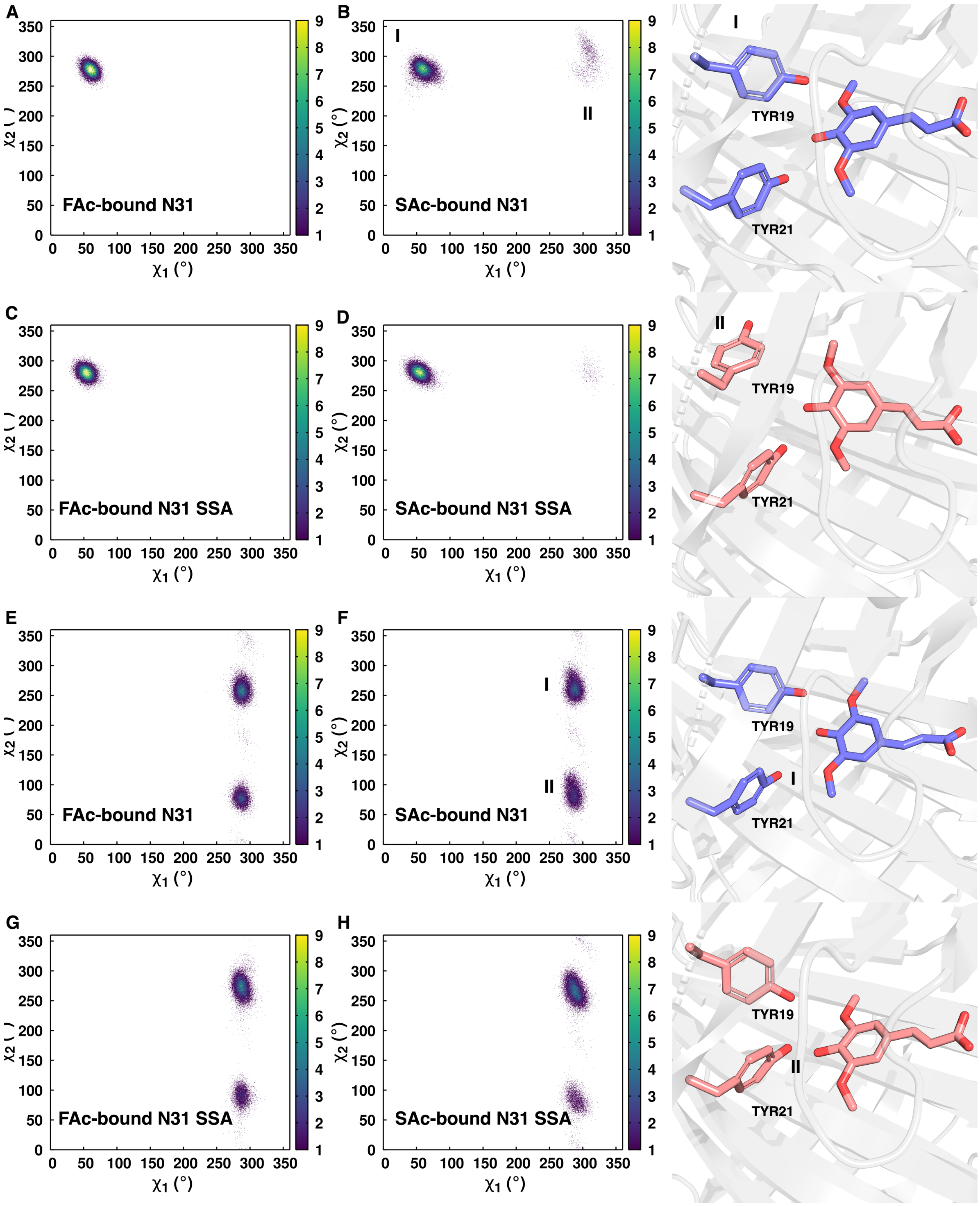

Figure S17: Joint distribution of the conformational space sampled by the χ_1 and χ_2 dihedral angles of the Tyr19 (A-D) and Tyr21 (E-H) side chains, during 5 x 100 μs simulations of the (A, E) FAc-bound N31, (B, F) SAc-bound N31, (C, G) FAc-bound N31 SSA and (D, H) SAc-bound N31 SSA. Data was collected every 200 ps.

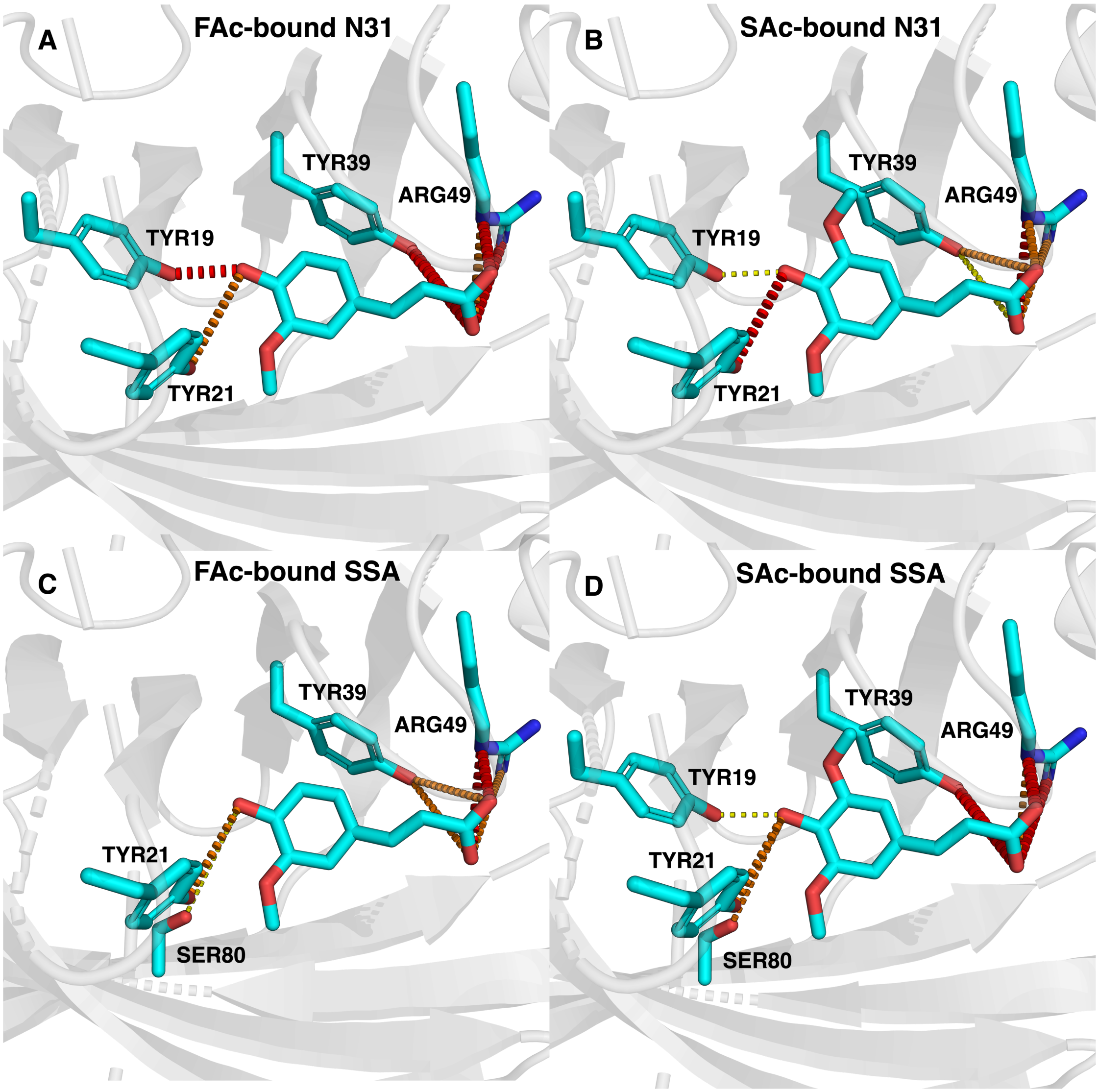

Figure S18: Protein-ligand hydrogen bonding interactions in FAc- and SAc-bound N31 and N31 SSA. Shown here are hydrogen bonds between key active site residues and the respective ligand that are present at ≥20% simulation time over 5 x 1 μs simulations of each system, calculated using CPPTRAJ (Roe and Cheatham 2013)and visualized with PyMOL (DeLano, 2021). Shown here are data for simulations of (A) FAc-bound N31, (B) SAc-bound N31, (C) FAc-bound SSA and (D) SAc-bound SSA, where line weight and color (yellow: lowest; red: strongest) are indicative of hydrogen bond persistence.

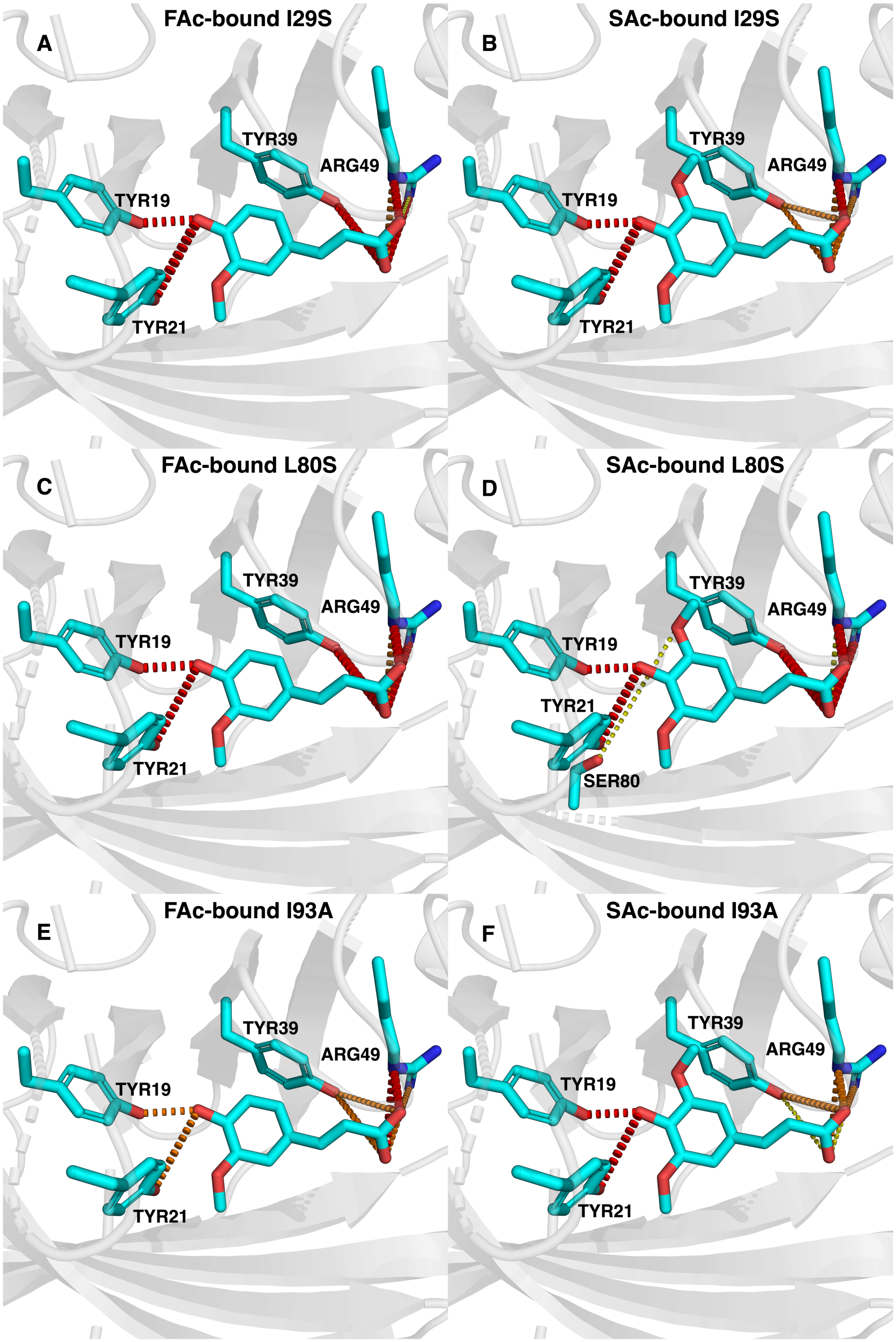

Figure S19: Protein-ligand hydrogen bonding interactions in FAc- and SAc-bound N31 single variants. Shown here are hydrogen bonds between key active site residues and the respective ligand that are present at ≥20% simulation time over 5 x 1 μs simulations of each system, calculated using CPPTRAJ (Roe and Cheatham 2013) and visualized with PyMOL (DeLano, 2021). Shown here are data for simulations of (A) FAc-bound Ile29Ser, (B) SAc-bound Ile29Ser, (C) FAc-bound Leu80Ser, (D) SAc-bound Leu80Ser, (E) FAc-bound Ile93Ala and (F) SAc-bound Ile93Ala, where line weight and color (yellow: lowest; red: strongest) are indicative of hydrogen bond persistence.

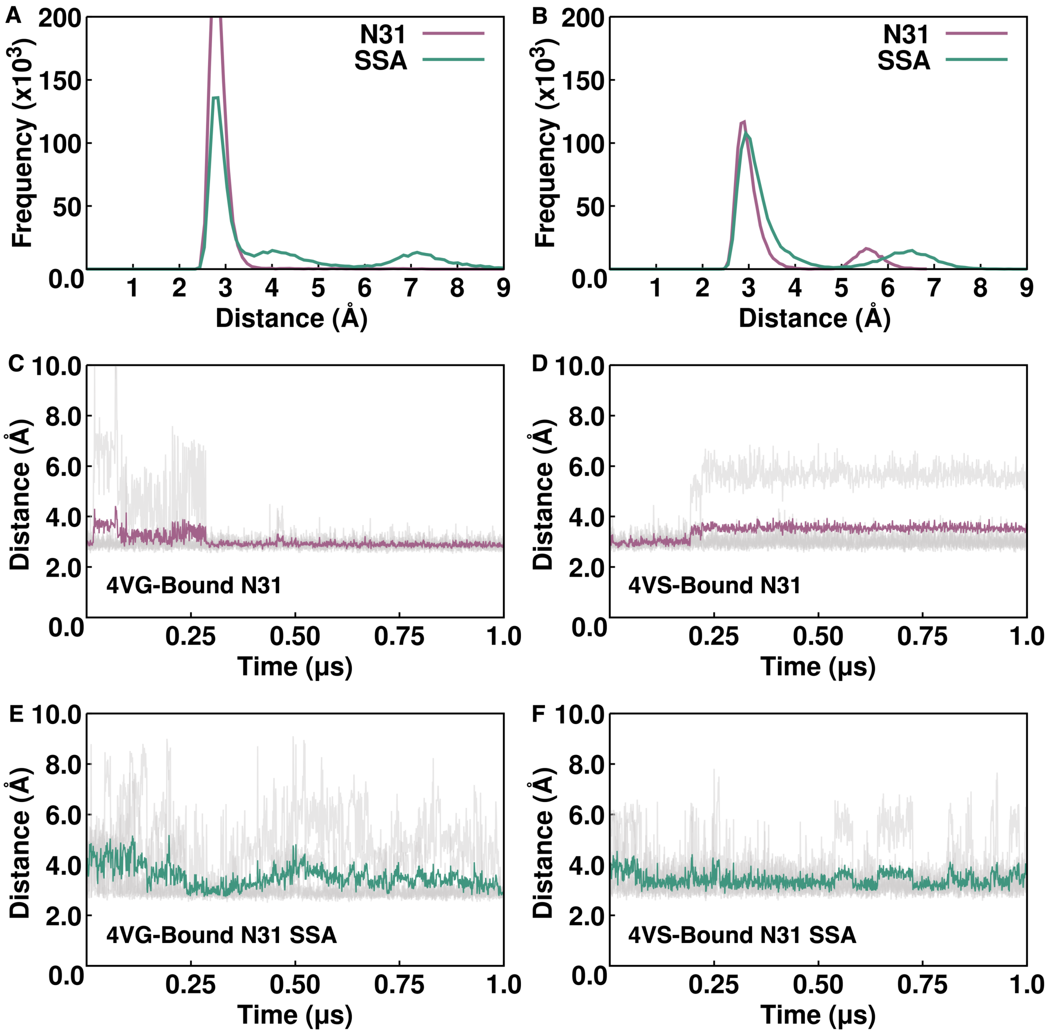

Figure S20: Histograms and time evolution of the distance between Tyr19 and the phenolic oxygen (4VG-Tyr19/4VS-Tyr19, Å) during 5 x 1 μs MD simulations of 4VG- and 4VS-bound N31 and N31 SSA PAD. Shown here are a comparison of histograms of (A) 4VG-bound N31 and N31 SSA, (B) 4VS bound N31 and N31 SSA, (C, E) time evolution of the 4VG-Tyr19 distance in (C) N31 and (E) N31 SSA, and (D, F) time evolution of the 4VS-Tyr19 distance in (D) N31 and (F) N31 SSA PAD, respectively. In panels (C-F), the shaded region shows the distances sampled by the individual trajectories, and the solid region shows the running average over all replicas. The corresponding data for the phenolic oxygen.

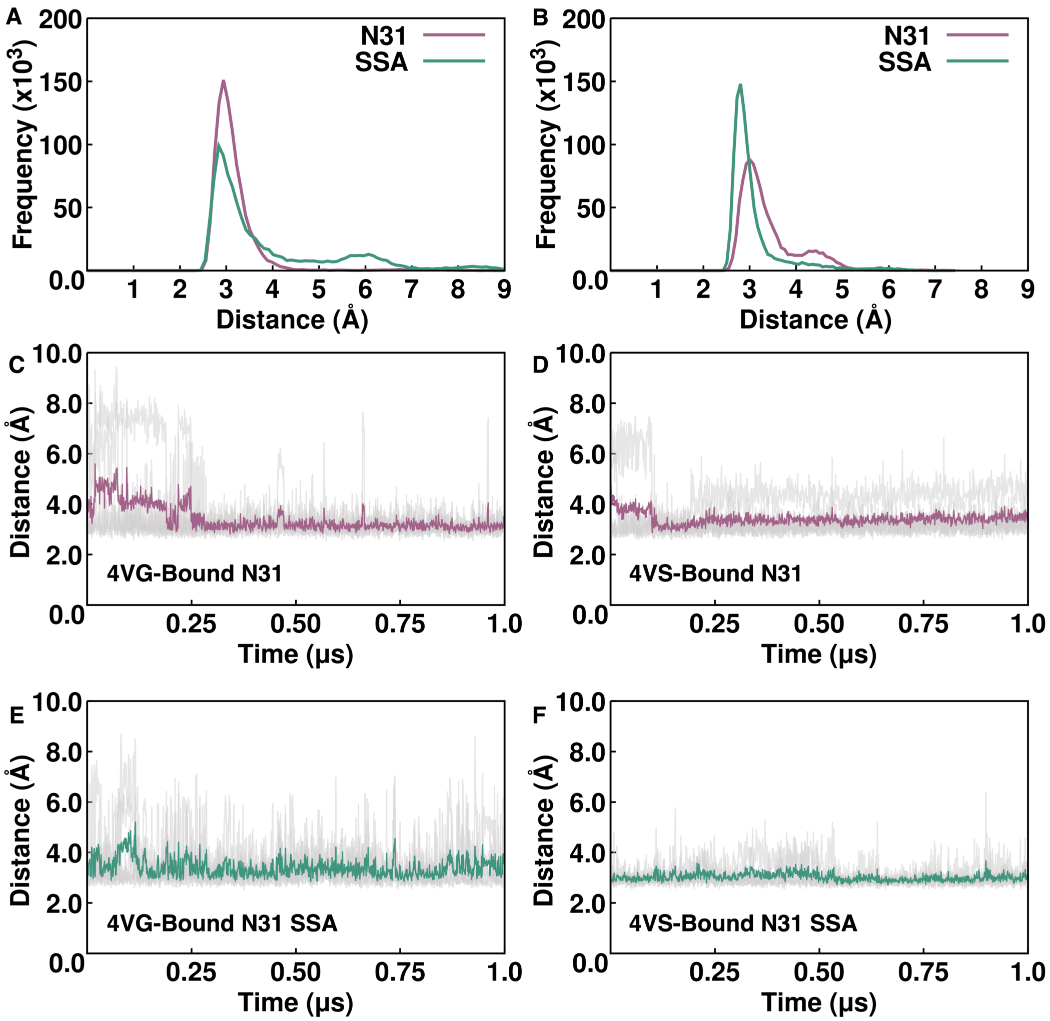

Figure S21: Histograms and time evolution of the distance between Tyr21 and the phenolic oxygen (4VG-Tyr21/4VS-Tyr21, Å) during 5 x 1 µs MD simulations of 4VG- and 4VS-bound N31 and N31 SSA PAD. Shown here are a comparison of histograms of (A) 4VG-bound N31 and N31 SSA, (B) 4VS bound N31 and N31 SSA, (C, E) time evolution of the 4VG-Tyr21 distance in (C) N31 and (E) N31 SSA, and (D, F) time evolution of the 4VS-Tyr21 distance in (D) N31 and (F) N31 SSA PAD, respectively. In panels (C-F), the shaded region shows the distances sampled by the individual trajectories, and the solid region shows the running average over all replicas. The corresponding data for the phenolic oxygen.

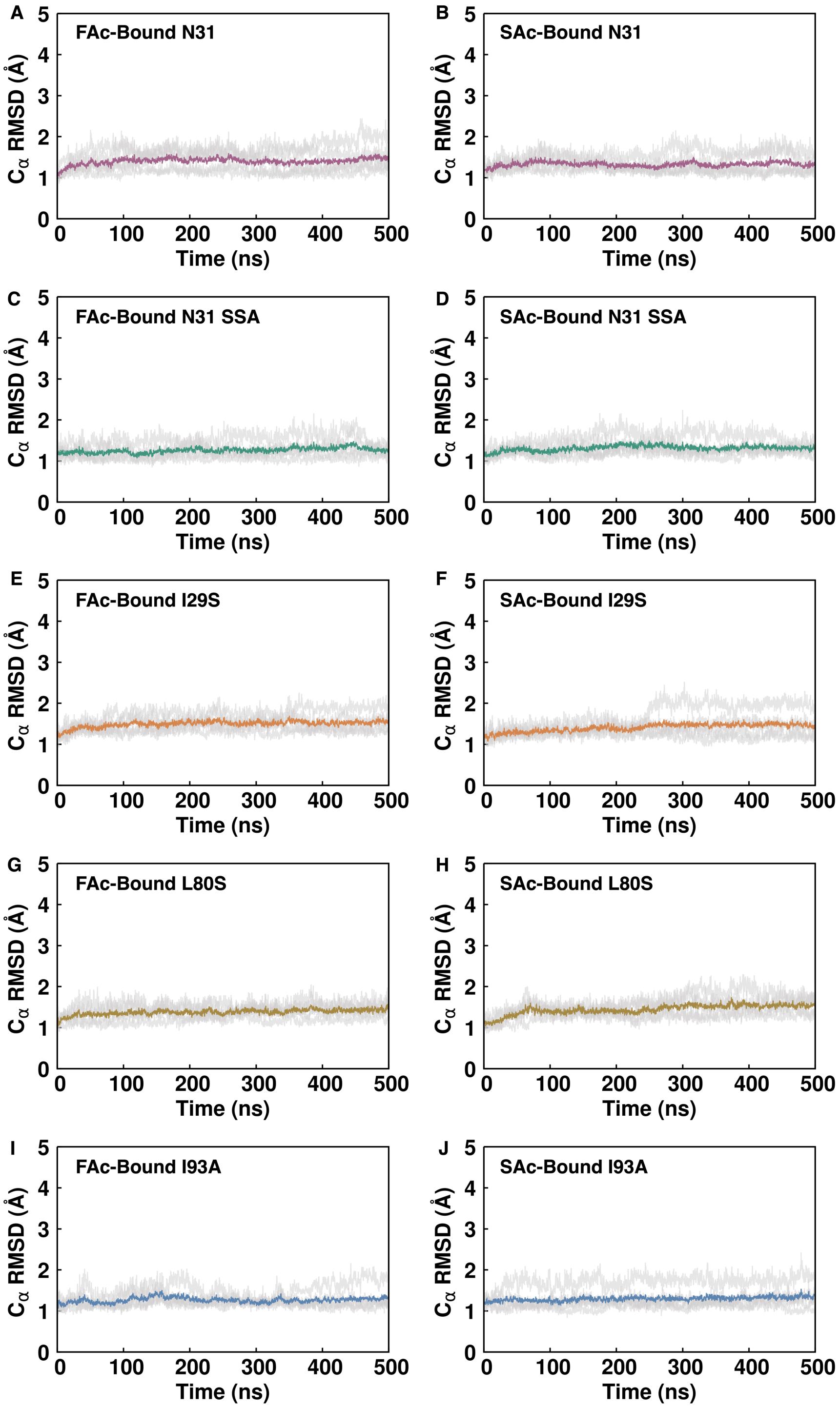

Figure S22: **Root mean square deviations (RMSD, Å)** of the Cα-atoms during MD simulations of wild-type (N31) and mutated (SSA) phenolic acid decarboxylases. (A) FAc-bound N31, (B) SAc-bound N31, (C) FAc-bound SSA, (D) SAc-bound SSA, (E) FAc-bound N31 I29S, (F) SAc-bound N31 I29S, (G) FAc-bound N31 L80S, (H) SAc-bound N31 L80S, (I) FAc-bound N31 I93A and (J) SAc-bound N31 I93A. Data was collected every 200 ps for 500 ns from 5 replicas of 1 μs length each to test convergence. The grey lines show the 5 individual runs, whilst the color solid line shows a rolling average RMSD from all 5 replicas for each system. Tail residues of the monomers (residues 151-157 and 309-317) were removed due to the high flexibility of those regions.

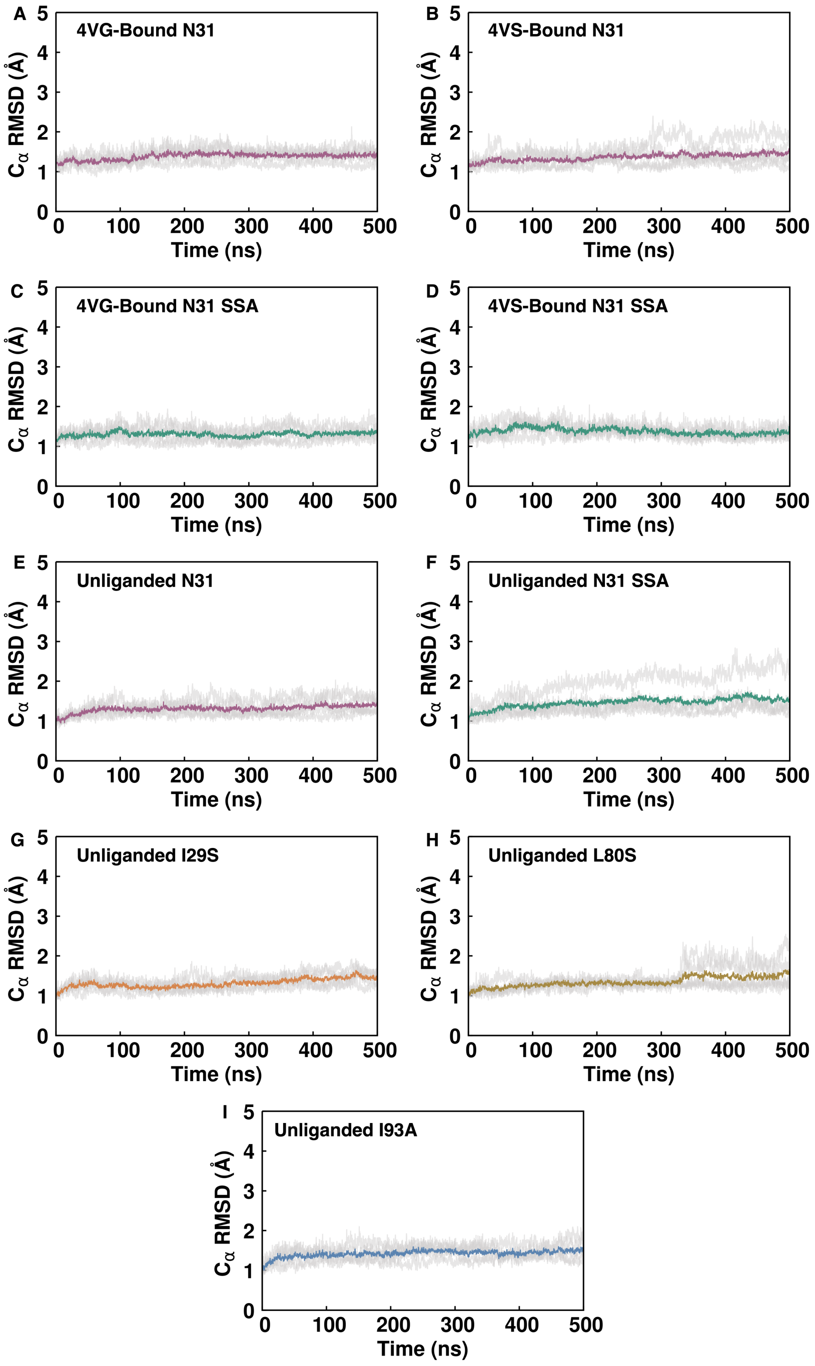

Figure S23: **Root mean square deviations (RMSD, Å)** of the Cα-atoms during MD simulations of wild-type (N31) and mutated (SSA) phenolic acid decarboxylases. (A) 4VG-bound N31, (B) 4VS-bound N31, (C) 4VG-bound SSA, (D) 4VS-bound SSA, (E) Unliganded N31, (F) Unliganded N31 SSA, (G) Unliganded N31 I29S, (H) Unliganded N31 L80S and (I) Unliganded N31 I93A. Data was collected every 200 ps for 500 ns from 5 replicas of 1 μs length each to test convergence. The grey lines show the 5 individual runs, whilst the color solid line shows a rolling average RMSD from all 5 replicas for each system. Tail residues of the monomers (residues 151-157 and 309-317) were removed due to the high flexibility of those regions.

Table S7: Average values and standard deviations of the key catalytic distances between the ligands FAc and SAc, and N31 and N31 SSA PAD.[a]

	Lig-Tyr19	Lig-Tyr21	Lig-Arg49	Lig-Glu72
**FAc-Bound N31**	3.06 ± 0.41	3.14 ± 0.34	3.97 ± 0.15	3.96 ± 0.23
**FAc-Bound SSA**	4.75 ± 1.72	3.05 ± 0.33	3.98 ± 0.17	4.03 ± 0.22
**SAc-Bound N31**	5.93 ± 1.58	3.11 ± 0.37	4.04 ± 0.15	4.04 ± 0.20
**SAc-Bound SSA**	4.80 ± 1.72	3.30 ± 0.75	4.00 ± 0.14	4.02 ± 0.20

[a] All distances are averages and standard deviations over 5 x 1 μs classical MD simulations of each system, with data collected every 200 ps. Lig-Tyr19 and Lig-Tyr21 measure the donor-acceptor distances between the Tyr19 and Tyr21 side chains and the phenolic oxygen of FAc/SAc bound to the protein in its deprotonated form, respectively, and Lig-Arg49 and Lig-Glu72 measure the donor-acceptor distance between the carboxylic carbon of FAc/SAc to the secondary amine carbon and carboxylic carbon, respectively.

Table S8: Number of solvent-ligand and solvent-Glu72 hydrogen bonds in simulations of N31, N31 SSA, and the N31 single variants.[a]

	FAc	SAc	Glu72 (FAc-bound)	Glu72 (SAc-bound)	Total (FAc-bound)	Total (SAc-bound)
**N31**	1.1 ± 1.2	2.2 ± 1.7	2.9 ± 1.3	2.8 ± 1.4	4.0 ± 1.6	5.0 ± 1.9
**N31 SSA**	2.3 ± 1.6	2.8 ± 2.0	3.0 ± 1.2	2.9 ± 1.3	5.3 ± 2.0	5.7 ± 2.2
**N31 I29S**	0.9 ± 1.1	1.9 ± 1.8	2.7 ± 1.4	2.3 ± 1.4	3.6 ± 1.2	4.2 ± 2.2
**N31 L80S**	1.2 ± 1.3	2.3 ± 1.6	2.8 ± 1.4	2.9 ± 1.3	4.0 ± 1.3	5.3 ± 1.6
**N31 I93A**	2.0 ± 1.7	1.9 ± 2.0	2.6 ± 14	2.1 ± 1.6	4.6 ± 1.7	4.0 ± 2.0

[a] All values are averages and standard deviations over 5 x 1 μs classical MD simulations of each system, with data collected every 1 ns. FAc-bound and SAc-bound indicate data analyzed in simulations of each variant with the respective ligand, and "Total" indicates the sum of solvent-ligand and solvent-Glu72 hydrogen bonds. Data calculated using VMD 2.0 hydrogen bond analysis.

Table S9: Average values and standard deviations of the center-of-mass (COM) of the ligands 4-VG and 4-VS distances to the center-of-mass N31 and N31 SSA PAD as well as twin-tyrosine distances between 4-VG and 4-VS, and N31 and N31 SSA PAD. [a]

	**COM-COM**	**Lig-Tyr19**	**Lig-Tyr21**
**4-VG-Bound N31**	2.55 ± 0.37	2.92 ± 0.35	3.22 ± 0.62
**4-VG-Bound SSA**	2.75 ± 0.71	4.02 ± 1.72	3.97 ± 1.48
**4-VS-Bound N31**	2.29 ± 0.44	3.46 ± 1.02	3.44 ± 0.63
**4-VS-Bound SSA**	2.81 ± 0.99	3.89 ± 1.39	3.13 ± 0.63

[a] All distances are averages and standard deviations over 5 x 1 μs classical MD simulations of each system, with data collected every 200 ps. Lig-Tyr19 and Lig-Tyr21 measure the donor-acceptor distances between the Tyr19 and Tyr21 side chains and the phenolic oxygen of FAc/SAc bound to the protein in its deprotonated form, respectively.

Table S10: Non-standard force field parameters used to describe the substrate ferulic acid (FAc) in our conventional molecular dynamics simulations.[a]

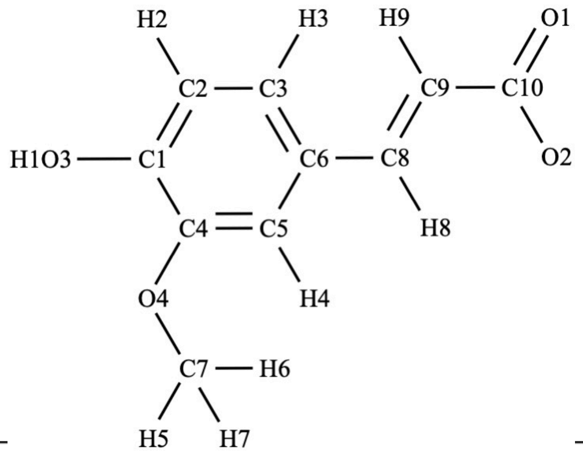

Atom Name	Atom Type	Charge
C1	ca	0.169825
O1	o	-0.702347
C2	ca	-0.209252
O2	o	-0.702347
C3	ca	-0.255175
O3	oh	-0.511439
C4	ca	0.153432
O4	os	-0.236401
C5	ca	-0.299281
C6	ca	0.199287
C7	c3	-0.150005
C8	ce	-0.255935
C9	cf	-0.147863
C10	c	0.677693
H1	ho	0.357388
H2	ha	0.149110
H3	ha	0.154880
H4	ha	0.144223
H5	h1	0.095620
H6	h1	0.095620
H7	h1	0.095620
H8	ha	0.115571
H9	ha	0.061777

[a] All parameters were obtained using the General AMBER Force Field 2 (GAFF2, (Wang et al. 2004)), as outlined in the Materials and Methods section of the main text.

Table S11: Non-standard force field parameters used to describe the substrate synaptic acid (SAc) in our conventional molecular dynamics simulations.[a]

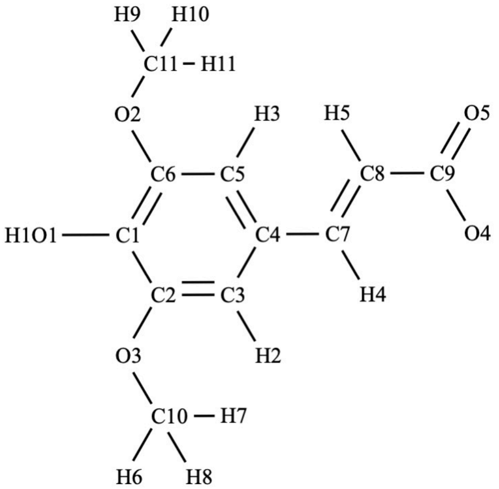

Atom Name	Atom Type	Charge
C1	ca	-0.006473
O1	oh	-0.596195
C2	ca	0.336737
O2	os	-0.281924
C3	ca	-0.460845
O3	os	-0.281924
C4	ca	0.208964
O4	o	-0.807526
C5	ca	-0.460845
O5	o	-0.807526
C6	ca	0.336737
C7	ce	-0.218172
C8	cf	-0.213703
C9	c	0.870954
C10	c3	-0.118110
C11	c3	-0.118110
H1	ho	0.420926
H2	ha	0.208734
H3	ha	0.208734
H4	ha	0.133920
H5	ha	0.096086
H6	h1	0.091593
H7	h1	0.091593
H8	h1	0.091593
H9	h1	0.091593
H10	h1	0.091593
H11	h1	0.091593

[a] All parameters were obtained using the General AMBER Force Field 2 (GAFF2, (Wang et al. 2004)), as outlined in the Materials and Methods section of the main text.

Table S12: Non-standard force field parameters used to describe the substrate synaptic acid (4-VG) in our conventional molecular dynamics simulations.[a]

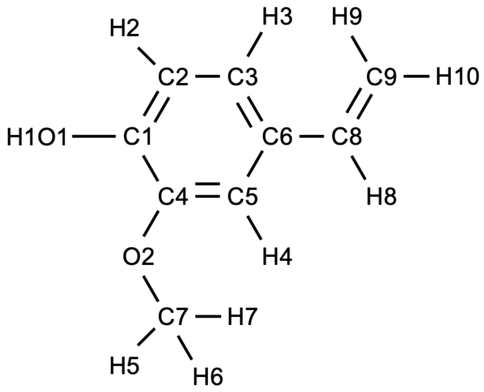

Atom Name	Atom Type	Charge
C1	ca	0.261796
O1	oh	-0.564048
C2	ca	-0.26546
O2	os	-0.272382
C3	ca	-0.237206
C4	ca	0.191305
C5	ca	-0.342146
C6	ca	0.163548
C7	c3	-0.147685
C8	ce	-0.136323
C9	c2	-0.454292
H1	ho	0.399129
H2	ha	0.202722
H3	ha	0.178874
H4	ha	0.175814
H5	h1	0.109431
H6	h1	0.109431
H7	h1	0.109431
H8	ha	0.140629
H9	ha	0.188716
H10	ha	0.188716

[a] All parameters were obtained using the General AMBER Force Field 2 (GAFF2, (Wang et al. 2004)), as outlined in the Materials and Methods section of the main text.

Table S13: Non-standard force field parameters used to describe the substrate synaptic acid (4-VS) in our conventional molecular dynamics simulations.[a]

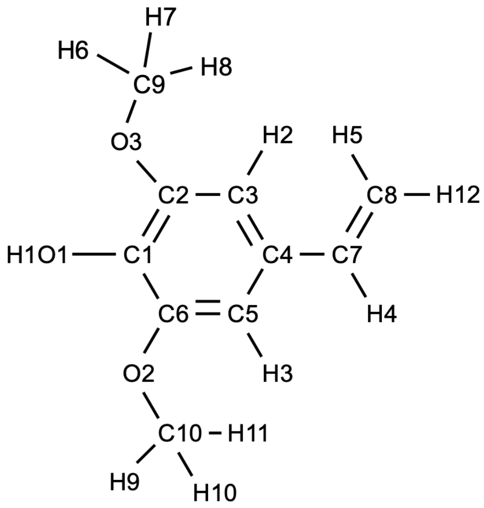

Atom Name	Atom Type	Charge
C1	ca	0.030926
O1	oh	-0.562202
C2	ca	0.319506
O2	os	-0.272258
C3	ca	-0.408326
O3	os	-0.272258
C4	ca	0.116761
C5	ca	-0.408326
C6	ca	0.319506
C7	ce	-0.044287
C8	c2	-0.483838
C9	c3	-0.127769
C10	c3	-0.127769
H1	ho	0.420464
H2	ha	0.197404
H3	ha	0.197404
H4	ha	0.114549
H5	ha	0.192408
H6	h1	0.100949
H7	h1	0.100949
H8	h1	0.100949
H9	h1	0.100949
H10	h1	0.100949
H11	h1	0.100949
H12	ha	0.192408

[a] All parameters were obtained using the General AMBER Force Field 2 (GAFF2, (Wang et al. 2004)), as outlined in the Materials and Methods section of the main text.

References

Case, D. A.; Cerutti, D. S.; Cruzeiro, V.; Darden, T. A.; Duke, R. E.; Ghazimirsaeed, M.; Giambasu, G. M.; Giese, T. J.; Götz, A. W.; Harris, J. A.; Kasavajhala, K.; Lee, T.; Li, Z.; Lin, C.; Liu, J.; Miao, Y.; Salomon-Ferrrer, R.; Shen, J.; Snyder, R.; Swails, J.; Walker, R. C.; Wang, J.; Wu, X.; Zeng, J.; Cheatham, T. E., III; Roe, D. R.; Roitberg, A.; Simmerling, C.; York, D. M.; Nagan, M. C.; Merz, K. M., JR. (2015) Recent Developments in Amber Biomolecular Simulations. J. Chem. Inf. Model. 65:7835–7843.

Case D. A.; Aktulga H. Metin; Belfon K.; Cerutti D. S.; Cisneros G. Andrés; Cruzeiro V. Wilian D.; Forouzesh N.; Giese T. J.; Götz A. W.; Gohlke H.; Izadi S.; Kasavajhala K.; Kaymak M. C.; King E.; Kurtzman T.; Lee T.-S.; Li P.; Liu J.; Luchko T.; Luo R.; Manathunga M.; Machado M. R.; Nguyen H. Minh; O'Hearn K. A.; Onufriev A. V.; Pan F.; Pantano S.; Qi R.; Rahnamoun A.; Risheh A.; Schott-Verdugo S.; Shajan A.; Swails J.; Wang J.; Wei H.; Wu X.; Wu Y.; Zhang S.; Zhao S.; Zhu Q.; Cheatham T. E.; Roe D. R.; Roitberg A.; Simmerling C.; York D. M.; Nagan M. C.; Merz K. M. (2023) AmberTools. J. Chem. Inf. Model. 63:6183–6191.

Corso G., Stärk H., Jing B., Barzilay R., Jaakkolaar T. (2022). DiffDock: Diffusion Steps, Twists, and Turns for Molecular Docking. Xiv:2210.01776v2 [q-bio.BM]

DeLano, W. L. (2021). The PyMOL Molecular Graphics System (Version 2.5.0). Schrödinger, LLC. Available at: https://pymol.org.

Dunbrack R. L., JR.; Cohen F. E. (1997) Bayesian statistical analysis of protein side-chain rotamer preferences. Protein Sci. 6, 1661–1681.

Frisch, M. J.; Trucks, G. W.; Schlegel, H. B.; Scuseria, G. E.; Robb, M. A.; Cheeseman, J. R.; Scalmani, G.; Barone, V.; Petersson, G. A.; Nakatsuji, H.; Li, X.; Caricato, M.; Marenich, A. V.; Bloino, J.; Janesko, B. G.; Gomperts, R.; Mennucci, B.; Hratchian, H. P.; Ortiz, J. V.; Izmaylov, A. F.; Sonnenberg, J. L.; Williams-Young, D.; Ding, F.; Lipparini, F.; Egidi, F.; Goings, J.; Peng, B.; Petrone, A.; Henderson, T.; Ranasinghe, D.; Zakrzewski, V. G.; Gao, J.; Rega, N.; Zheng, G.; Liang, W.; Hada, M.; Ehara, M.; Toyota, K.; Fukuda, R.; Hasegawa, J.; Ishida, M.; Nakajima, T.; Honda, Y.; Kitao, O.; Nakai, H.; Vreven, T.; Throssell, K.; Montgomery Jr., J. A.; Peralta, J. E.; Ogliaro, F.; Bearpark, M. J.; Heyd, J. J.; Brothers, E. N.; Kudin, K. N.; Staroverov, V. N.; Keith, T. A.; Kobayashi, R.; Normand, J.; Raghavachari, K.; Rendell, A. P.; Burant, J. C.; Iyengar, S. S.; Tomasi, J.; Cossi, M.; Millam, J. M.; Klene, M.; Adamo, C.; Cammi, R.; Ochterski, J. W.; Martin, R. L.; Morokuma, K.; Farkas, O.; Foresman, J. B.; Fox, D. J. (2016) Gaussian 16, Revision B.01.

Hopkins C. W.; Le Grand S.; Walker R. C.; Roitberg A. E. (2015) Long-Time-Step Molecular Dynamics through Hydrogen Mass Repartitioning. J. Chem. Theory Comput. 11:1864–1874.

Humphrey, W., Dalke, A. and Schulten, K. (1996) VMD - Visual Molecular Dynamics. Journal of Molecular Graphics 14:33–38.

Izadi S.; Anandakrishnan R.; Onufriev A. V. (2014) Building Water Models: A Different Approach. J. Phys. Chem. Lett. 5:3863–3871.

Myrtollari K.; Calderini E.; Kracher D.; Schöngaßner T.; Galušić S.; Slavica A.; Taden A.; Mokos D.; Schrüfer A.; Wirnsberger G.; Gruber K.; Daniel B.; Kourist R. (2024) Stability Increase of Phenolic Acid Decarboxylase by a Combination of Protein and Solvent Engineering Unlocks Applications at Elevated Temperatures. ACS Sustainable Chem. Eng., 12, 3575–3584.

Olsson M. H. M.; Søndergaard C. R.; Rostkowski M.; Jensen J. H. (2011) PROPKA3: Consistent Treatment of Internal and Surface Residues in Empirical pKa Predictions. J. Chem. Theory Comput. 7:525–537.

Payer, S. E.; Sheng, X.; Pollak, H.; Wuensch, C.; Steinkellner, G.; Himo, F.; Glueck, S. M.; Faber, K. (2017) Exploring the Catalytic Promiscuity of Phenolic Acid Decarboxylases: Asymmetric, 1,6-Conjugate Addition of Nucleophiles Across 4-Hydroxystyrene. Advanced Synthesis & Catalysis 359:2066–2075.

Rodríguez H.; Angulo I.; las Rivas B. de; Campillo N.; Páez J. A.; Munoz R.; Mancheno J. M. (2010) p-Coumaric acid decarboxylase from Lactobacillus plantarum: Structural insights into the active site and decarboxylation catalytic mechanism. Proteins 78:1662–1676.

Roe, D. R.; Cheatham, T. E. 3. PTRAJ and CPPTRAJ: Software for Processing and Analysis of Molecular Dynamics Trajectory Data. (2013) Journal of chemical theory and computation 9:3084–3095.

Ryckaert, J.-P.; Ciccotti, G.; Berendsen, H. J. (1977) Numerical integration of the cartesian equations of motion of a system with constraints: molecular dynamics of n-alkanes. Journal of Computational Physics 23:327–341.

Schmidtke, P.; Bidon-Chanal, A.; Luque, F. J.; Barril, X. (2011) MDpocket: open-source cavity detection and characterization on molecular dynamics trajectories. Bioinformatics 27:3276–3285.

Sheng X.; Lind M. E. S.; Himo F. (2015) Theoretical study of the reaction mechanism of phenolic acid decarboxylase. FEBS J. 24:4703–4713.

Tian C.; Kasavajhala K.; Belfon K.; Raguette L.; Huang H.; Migues A. N.; Bickel J.; Wang Y.; Pincay J.; Wu Q.; Simmerling C. (2020) ff19SB: Amino-Acid-Specific Protein Backbone Parameters Trained against Quantum Mechanics Energy Surfaces in Solution. J. Chem. Theory Comput. 1:528–552.

Wang J.; Wang W.; Kollman P. A.; Case D. A. (2006) Automatic atom type and bond type perception in molecular mechanical calculations. J. Mol. Graph. Model. 2:247–260.

Wang J.; Wolf R. W.; Caldwell J. W.; Kollman P. A.; Case D. A. (2004) Development and testing of a general amber force field. J. Comput. Chem. 25:1157–1174

Woods R. J.; Chappelle R. (2000) Restrained electrostatic potential atomic partial Restrained electrostatic potential atomic partial charges for condensed-phase simulations of carbohydrates. J. Mol. Struct. (Theochem), 527:149-156.
